# A High Density Consensus Genetic Map of Tetraploid Cotton That Integrates Multiple Component Maps through Molecular Marker Redundancy Check

**DOI:** 10.1371/journal.pone.0045739

**Published:** 2012-09-24

**Authors:** Anna Blenda, David D. Fang, Jean-François Rami, Olivier Garsmeur, Feng Luo, Jean-Marc Lacape

**Affiliations:** 1 Department of Genetics and Biochemistry, Clemson University, Clemson, South Carolina, United States of America; 2 Department of Biology, Erskine College, Due West, South Carolina, United States of America; 3 Cotton Fiber Bioscience Research Unit, USDA-ARS-SRRC, New Orleans, Louisiana, United States of America; 4 CIRAD, UMR AGAP, Montpellier, France; 5 School of Computing, Clemson University, Clemson, South Carolina, United States of America; New Mexico State University, United States of America

## Abstract

A consensus genetic map of tetraploid cotton was constructed using six high-density maps and after the integration of a sequence-based marker redundancy check. Public cotton SSR libraries (17,343 markers) were curated for sequence redundancy using 90% as a similarity cutoff. As a result, 20% of the markers (3,410) could be considered as redundant with some other markers. The marker redundancy information had been a crucial part of the map integration process, in which the six most informative interspecific *Gossypium hirsutum*×*G. barbadense* genetic maps were used for assembling a high density consensus (HDC) map for tetraploid cotton. With redundant markers being removed, the HDC map could be constructed thanks to the sufficient number of collinear non-redundant markers in common between the component maps. The HDC map consists of 8,254 loci, originating from 6,669 markers, and spans 4,070 cM, with an average of 2 loci per cM. The HDC map presents a high rate of locus duplications, as 1,292 markers among the 6,669 were mapped in more than one locus. Two thirds of the duplications are bridging homoeologous A_T_ and D_T_ chromosomes constitutive of allopolyploid cotton genome, with an average of 64 duplications per A_T_/D_T_ chromosome pair. Sequences of 4,744 mapped markers were used for a mutual blast alignment (BBMH) with the 13 major scaffolds of the recently released *Gossypium raimondii* genome indicating high level of homology between the diploid D genome and the tetraploid cotton genetic map, with only a few minor possible structural rearrangements. Overall, the HDC map will serve as a valuable resource for trait QTL comparative mapping, map-based cloning of important genes, and better understanding of the genome structure and evolution of tetraploid cotton.

## Introduction

Four cotton (*Gossypium*) species (i.e., two diploid species, *G. arboreum* L. and *G. herbaceum* L. (n = x = 13) of the A genome, and two allotetraploid species, *G. barbadense* L. and *G. hirsutum* L. (n = 2x = 26) of the AD genome) contribute to the production of natural fiber around the world [1]. Of these species, *G. hirsutum* (Upland cotton) is the most widely grown worldwide, accounting for about 95% of both acreage and fiber production (National Cotton Council, 2012, http://www.cotton.org/econ/cropinfo/index.cfm). *G. barbadense* (Pima cotton) produces fiber of premium quality, but has lower yield per hectare compared to the Upland cotton, and accounts for about 2–3% of acreage. The diploid species *G. arboreum* and *G. herbaceum* are grown only in very limited areas in a few countries, such as India and Pakistan.

Cultivated tetraploid cotton species *G. hirsutum* (*Gh*) and *G. barbadense* (*Gb*) have very low intra-specific molecular polymorphism, as revealed by a variety of molecular markers [2]–[7]. Consequently, the majority of saturated genetic maps had been constructed using interspecific *Gh*×*Gb* segregating populations [8]–[17]. Reinisch et al. [16] reported the first detailed RFLP genetic map in cotton using 57 F_2_ plants derived from a cross (*Gh* race palmeri and *Gb* acc. K101). Using the same population, Rong et al. [18] augmented the same map to 2,584 total loci. Majority of the loci were represented by the RFLP markers. This map provided one of the first insights into the genome structure and evolution of the allotetraploid cotton. Due to the limited portability of RFLP markers, researchers later turned to the PCR-based markers in genetic map construction. Nguyen et al. [11] constructed an 1,160 loci (AFLP, RFLP, and SSR) map using 75 BC_1_ plants from a cross [(*Gh* Guazuncho 2×*Gb* VH8-4602)×*Gh* Guazuncho 2]. Yu et al. [10] constructed a linkage map from a cross (*Gh* CRI 36×*Gb* Hai 7124) using 186 F_2_ plants. The map consisted of 1,097 loci including sequence-related amplified polymorphism (SRAP), target-region amplified polymorphism (TRAP), AFLP, and SSR markers. SSRs became the marker of choice in the recent cotton genetic map constructions due to their co-dominance, portability and abundance. Guo et al. [12] constructed the first comprehensive SSR map using 138 BC_1_ plants derived from a cross [(*Gh* TM-1×*Gb* Hai 7124)×*Gh* TM-1], with 1,790 loci further augmented to 2247 loci [8]. Lacape et al. [9] reported a consensus genetic map integrating 1,745 marker loci (AFLP, RFLP and SSR) from combined data from 2 populations (BC_1_ and RIL, totalling 215 individuals) involving *Gh* Guazuncho 2 and *Gb* VH8-4602. Yu et al. [13] used 141 BC_1_ plants derived from a cross [(*Gh* Emian 22×*Gb* 3–79)×*Gh* Emian 22] to construct a map containing exclusively SSR markers (2,316 loci). Recently, Yu et al. [14] reported a high-density SSR and SNP genetic map, with 2,072 loci, using 186 recombinant inbred lines (RILs) derived from a cross (*Gh* TM-1×*Gb* 3–79). Though less comprehensive in genome coverage, other maps have been constructed using *Gh* intraspecific populations [19]–[22], a cross between *G. hirsutum* and *G. tomentosum* [23], and lastly, different crosses involving different diploid *Gossypium* species [18], [24], [25].

An individual genetic map has certain limitations, such as large gaps due to the lack of polymorphism in particular genomic regions. Moreover, marker order errors might be present and unnoticed in a map constructed using a single population. Consensus map constructed utilizing the information from multiple segregating populations provides a very important reference resource. It gives an opportunity to map larger number of loci as compared to most single crosses, thus increasing the number of potentially useful markers across divergent genetic backgrounds. In addition to providing opportunities to validate the marker order, consensus map also provides greater genome coverage [26]–[28]. Different approaches and tools had been reported for consensus genetic map construction. For example, software program JoinMap was used in rye [28] and barley [29] employing all available information from each data set; MergeMap based on directed acyclic graphs (DAGs) was used in cowpea [30] and Brassica [31], and ILMap was used in a comparative approach [32]. Lastly, Mace et al. [27] used simple projection (“neighbours” approach) to integrate six component maps in barley using one map as a ‘base’ or reference map.

In cotton, the only major large-scale map integration effort [33] used the graph theory and algorithms utilized in the Travelling Salesman Problem (TSP). The authors integrated map data from 28 mapping reports published before 2009 and proposed a consensus order of markers for a tetraploid cotton genome reference map. However, the map has several limitations. First, the markers were not checked for redundancy; consequently, the unique loci might be over-inflated in the map. Second, the map provides only the order of the markers on chromosomes, but not the actual distances between the markers. Third, three new high-density maps were published since 2009 [9], [13], [14].

Because cotton SSRs had been developed by more than a dozen research groups without much coordination, SSR marker redundancy became an inevitable and common problem for efficient use of these markers. Thus, SSR redundancy elimination was one of the key steps in the construction of the proposed consensus genetic map of tetraploid cotton. Here we report the SSR redundancy elimination as the first step in the construction of a high density consensus genetic. Next, we constructed a consensus genetic map with 8,254 loci integrating six independent component maps including the most recently published. Finally, using mutual blast hits between the mapped markers and the 13 scaffolds of the *G. raimondii* genome (released on January 6, 2012, http://www.phytozome.net/cotton.php), we investigated the collinearity between the proposed consensus map and the cotton D genome. Overall, the high density consensus tetraploid cotton genetic map reported here will be very valuable for the studies of cotton genome structure and will further facilitate cotton breeding with molecular marker technology.

## Results

### Marker redundancy

The sequence similarity between primers was checked as the first indication of possible marker redundancy. The 17,448 pairs of SSR primers from the CMD collection were aligned to each other. At a 95% similarity cutoff, roughly corresponding to a single base difference between 2 primers, we obtained alignments between pairs of primers from 3,202 different markers (related either to both forward and reverse primers, or to only one of the two). At a 100% similarity cutoff, 2,515 different markers displayed exact similarity between their primers. Considering that sequence redundancy based on the approximate 20 nucleotides of primers was reductive, we used the complete SSR-containing clone sequences in the second series of sequence alignments. A fairly low sequence similarity threshold (80% of a short sequence) was used to align the 17,343 sequences available in CMD as of May 2012. For the subset of around 7,000 markers, which also had map information, we used their (similar) chromosome localizations on the component maps as an additional evidence of the suspected redundancy. Due to the existence of extensive homoeologous duplications of multi-copy markers in the polyploid genome, the localization on two homoeologous chromosomes was also considered as possible confirmation of redundancy.

**Figure 1:**
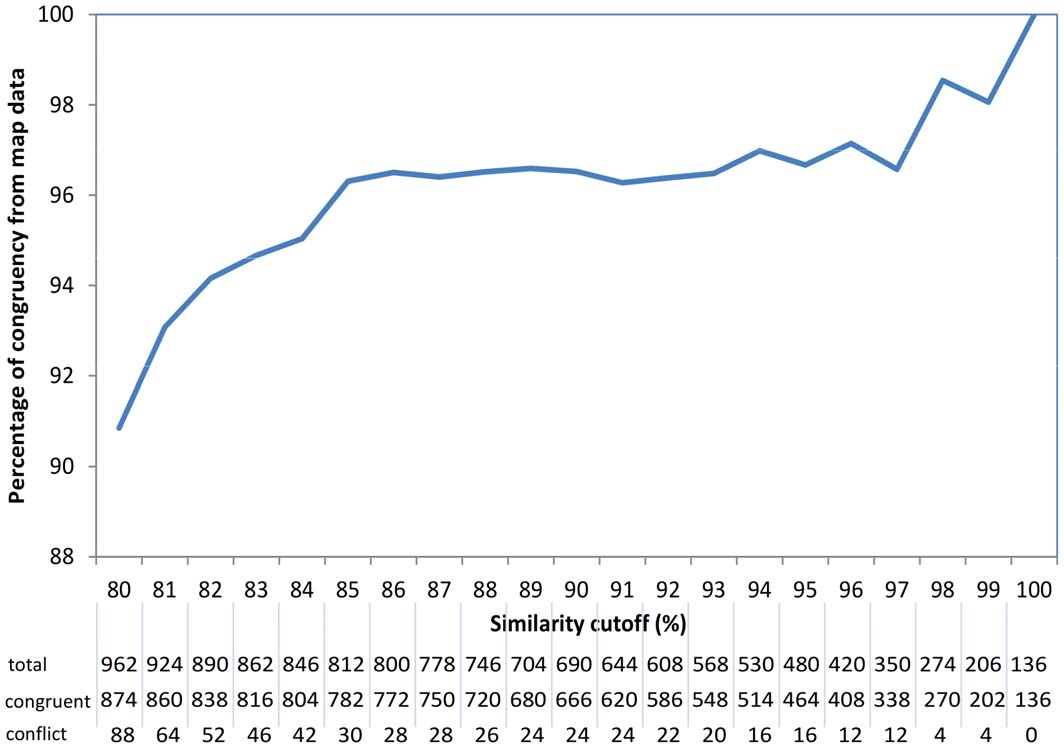
Supporting evidence of marker redundancy based on mapping information as a function of sequence similarity threshold. Number of pairs of suspected redundant markers associated with map information (whether map localization was congruent or conflicting between the 2 markers).

At a full sequence similarity threshold of 80%, 7,611 (44% of the total) markers were identified to have a hit to between 1 and 31 other markers. At this threshold, 962 pairs of suspected redundant markers comprised 2 markers mapped in at least one among the component maps, with 874 cases, or 90.9%, where the 2 markers were on the same chromosome, and 88 cases, or 9.1%, where map data were in conflict. The percentage of agreement with map data progressively increased to above 96% when the sequence similarity threshold was set at 85% and above (Figure 1); and finally using 100% sequence similarity, all of the 136 pairs of suspected redundant markers were corroborated by map data. We used the 90% cutoff as a conservative sequence similarity threshold to minimize false positives when assigning 2 markers to the same cluster of identical-by-sequence markers. At this threshold, 666 among 690 marker pairs (96.5%) with map information were congruent and 24, or 3.5%, were contradicted by their map position.

At this threshold of 90% sequence similarity, about one third (5,896 of the 17,343) of the markers were assigned to 2,357 different groups of similarity (further referred to as clusters, with the common name abbreviation CLU), each comprising between 2 and 14 markers (Table S1).

The largest cluster, CLU216, comprised 14 markers of pairwise sequence similarity ranging between 91% and 98%. This cluster included GA__Ea0004J17 (PGML marker), MUCS296, MUSS267, NAU1042, NAU1359, and 9 markers of the HAU series (HAU014, HAU0234, HAU046, HAU0878, HAU2570, HAU2811, HAU2812, HAU2969, and HAU3115). Of those markers, 2 were mapped, NAU1042 on 2 chromosomes, c5 (maps TH and T3) and its homoeolog c19 (maps TH, T3, GV and CH), and HAU0878 on c5 (map E3). Cluster CLU214 included 9 markers of pairwise similarity ranging between 93% and 98%, from the largest number (7) of different libraries: PGML (GA__Ea0004D22), HAU (HAU2758, HAU3090, HAU3136), MGHES (MGHES17), MUCS (MUCS150), MUSS (MUSS249 as only marker mapped on c12 on map T3), NAU (NAU1115), and NBRI (NBRI_Gh_E_1536).

Statistics of sequence similarity of markers within and between the 18 major SSR libraries (378 markers not considered) is presented in Table S2. Among the 5,363 markers pairs assigned to 2,357 clusters, the libraries with highest level of cross-redundancy were HAU and NAU libraries with 872 marker pairs with high sequence similarity. Overall, the redundancy hits of a single library with all others were higher in library HAU (1,776 hits), NAU (1,763), 3 libraries of the MU series (1,180, shared between 669 MUSS, 494 MUCS, and 17 MUSB), MON (647), and NBRI (400). Redundancy within a library was the highest for HAU (646 pairs or 19.1% of a total of 3,373 marker sequences) and NAU (593 pairs or 18.3% of 3,249 marker sequences), and to a lower extent for MON (183 pairs), MUSB (168 pairs), and NBRI (146 pairs) (Table S2).

It should be emphasized that in our project, marker redundancy check was aimed at recovering groups of identical-by-sequence markers even though (1) they may contain several microsatellite motifs (explaining why they may have different names), or (2) they may represent homoeolog copies from A and D subgenomes (with a sequence similarity above 90%, they would co-assemble). It was then expected that these markers would segregate and map at similar locations.

### Verification and merging of component maps

**Figure 2:**
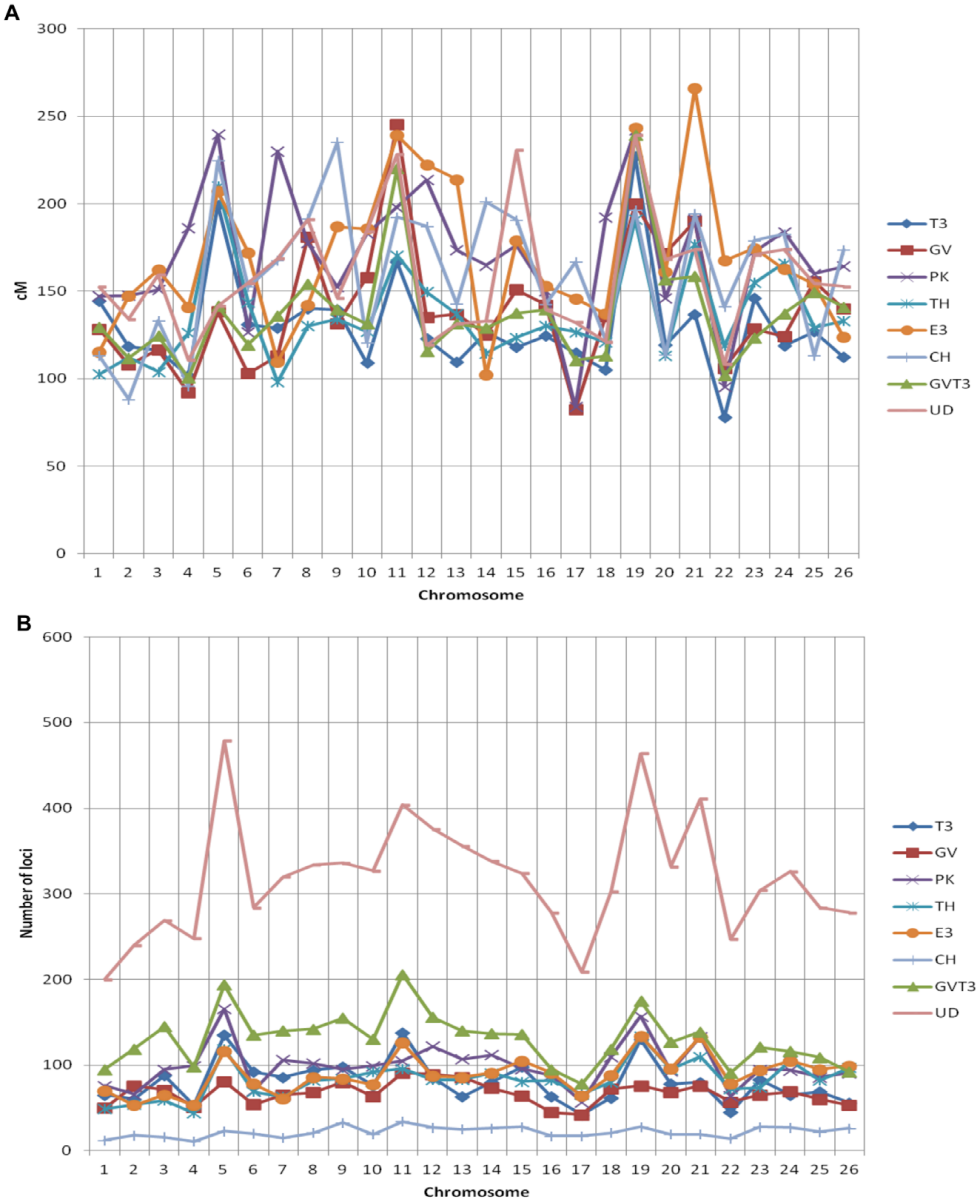
Comparison of six component maps and HDC map. The X axis indicates each individual chromosome. The Y axis represents genetic distance (A), or number of markers (B).

The component maps (Table 1) varied by the parental genotypes of the population, except for 3 parents in common: TM-1 (*Gh*) common to T3 and TH, Hai 7124 (*Gb*) common to TH and CH, and 3–79 (*Gb*) common to E3 and T3. The TH and E3 maps were derived from BC_1_ populations, PK and CH maps were from F_2_ populations, T3 map was from a RIL, and GV was a consensus map of BC_1_ and RIL populations. Total map length of the component maps varied between 3,380 cM (T3) and 4,448 (PK), and the loci number varied between 1,080 (CH) and 2,584 (PK) (Table 1). Distribution of genetic distances and number of loci among the 26 chromosomes are presented in Figure 2A and 2B, respectively. Except for the PK map, which was essentially covered with RFLP markers, other five maps contained a high proportion of SSR markers (Table 1). Altogether, these six component maps contained 12,044 loci. Of those, 606 non-informative markers (such as anonymous AFLPs, TRAPs, and SRAPs) were not considered in the map integration. Following curation of marker redundancy and map inversions that prevent map integration, 10,330 loci of the 6 component maps were actually used in the consensus map construction (Table 1).

**Table 1:** Summary of six component maps used to construct the cotton HDC map.

Map code	Parents1)	Generation	Pop size	Genetic distance (cM)2)	No. of mapped markers						No unique loci4)	No loci for map integration5)	References
					Total	RFLP	AFLP	SSR	SNP	Others3)			
**T3**	TM-1×3–79	RIL	186	3,380	2072			1,825	247		677	1,977	[14]
**GV**	(Gua×VH8)×Gua, and Gua×VH8	BC_1_-RIL6)	215	3,637	1,745	190	715	781		59	778	1,671	[9]
**PK**	Palmeri×K101	F_2_	57	4,448	2,584	2,459		124		1	1352	2,559	[18]
**TH**	(TM-1×Hai 7124)×TM-1	BC_1_	138	3,541	2,247		71	1,865	10	301	796	1,892	[8]
**E3**	(Emian22×3–79)×Emian22	BC_1_	141	4,419	2,316			2,311		5	480	1,814	[13]
**CH**	CRI 36×Hai 7124	F_2_	186	4,418	1,080		93	690		297	33	417	[10]
**Total**					12,044	2,649	879	7,596	257	663	4,116	10,330	

1) all female and recurrent parents belong to *G. hirsutum*, and all male parents belong to *G. barbadense*.

2) T3, GV, TH and E3 maps were constructed using JoinMap program. PK and CH maps were constructed using MapMaker program.

3) including target region amplification polymorphism (TRAP), sequence-related amplified polymorphism (SRAP), isozyme, gene-derived, and morphological markers.

4) the loci not present in any one of the other five maps.

5) TRAP, SRAP, morphological and some AFLP markers could not be easily traced back to their description, and were not included in consensus map integration.

6) a consensus map constructed with JoinMap (*combine data for map integration*) using 75 BC_1_ and 140 RIL populations.

There was a large discrepancy between the total number of markers in common between the maps (same marker on same chromosome) derived from the original published data (5,043 markers in common in Table 2A) and the number of markers in common after the data curation (2,789 duplicates in Table 2B). More specifically, the number of multi-copy markers in paralogy (2 or more loci mapped by a given marker on the same chromosome) decreased from 508 in published data to 168 in our report after the curation of marker redundancy (values along the diagonals of Table 2A and 2B). The number of bridge markers on the 6 component maps in terms of merging with any other maps was fairly good, except for PK map which had a sufficient level of connectivity only with the GV map (Table 2B). The 179 bridges between the GV and PK maps were essentially represented by RFLP loci. However, PK map contributed the highest number of the unique markers (Table 1), and helped to enrich the consensus map.

**Table 2:** Number of common loci (same chromosome) between map pairs.

Map	T3	GV	PK	TH	E3	CH	Total “between”
**A** 1)							
**T3**	105	422	63	408	837	351	2081
**GV**		37	221	350	323	329	1645
**PK**			76	69	64	79	496
**TH**				66	929	283	2039
**E3**					193	315	2468
**CH**						31	1357
**B** 1)							
**T3**	13	258	37	226	398	180	1,099
**GV**		17	179	196	161	178	972
**PK**			75	34	35	39	324
**TH**				19	583	142	1,181
**E3**					32	143	1,320
**CH**						12	682

Counts were made before (A) and after (B) marker redundancy curation.

1) values between maps were calculated differently from the values within map (diagonal), i.e. for counts between-maps and markers with paralogs loci, only a single locus was considered.

The most informative markers in terms of transferability between the component maps were SSR markers prefixed with the BNL name (the first published cotton SSR project). Thirty-five SSRs mapped on all 6 component maps were from the BNL SSR project, and 87 SSRs mapped on 5 of the 6 maps, included 69 BNL markers, 6 CIR markers and 12 JESPR markers. However, several of those markers appeared to be redundant.

### Construction of the high density consensus cotton map

**Figure 3:**
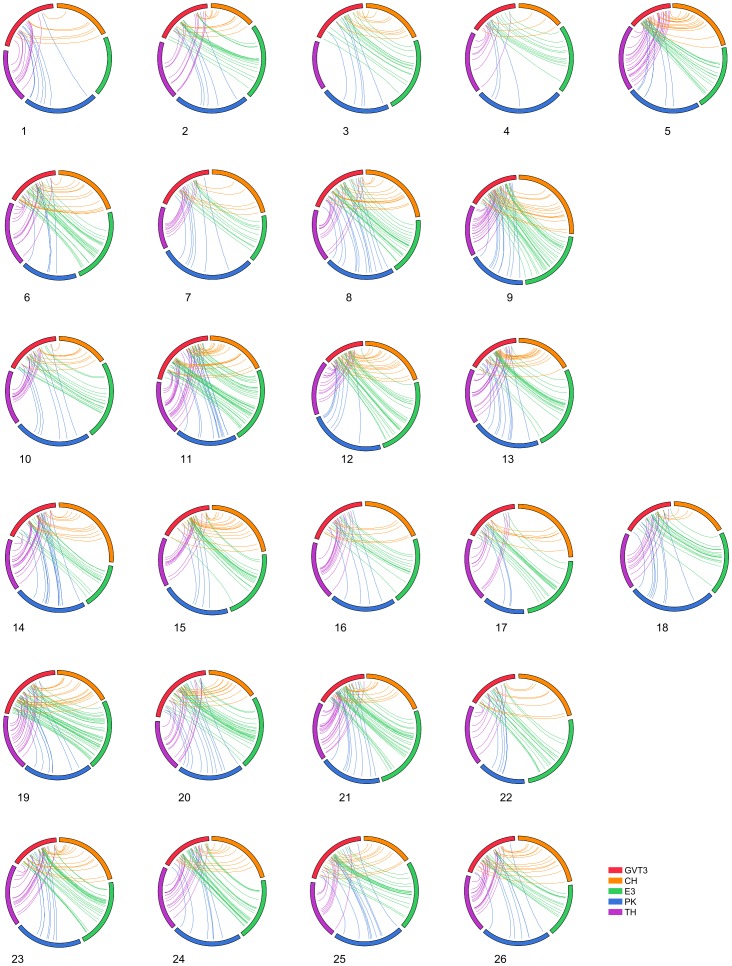
Collinearity of loci order in component maps. Loci that are common between pairs of maps are connected by lines. Connectivity between each one of the 4 components maps and GVT3 (consensus map GVT3 is in red colour and the 4 component maps are represented in blue for map PK, green for map E3, purple for map TH, orange for CH). Only collinear connectors used in the stage two of map integration (iterative projection of the 4 maps on the GVT3 map) are presented. Two chromosomes were not considered at this stage (no connectors with GVT3), c1 from E3 and c3 from TH, because of too many inconsistencies with GVT3. See text for population/map acronyms.

Various features of the two consensus maps, GVT3 map as an intermediate version integrating the component maps GV and T3, and final high density consensus map (further referred as HDC map) integrating GVT3 map with the 4 remaining component maps, are summarized in Table 3. Collinearity of loci order between the GV and T3 maps and their merging into the GVT3 map (step 1 of map integration) is presented in Figure S1. The overall merging connectivity of the GVT3 map (step 2 of integration) with each of the 4 additional component maps under consideration is presented in Figure 3.

**Table 3:** Comparison of the intermediate GVT3 map and the final HDC map.

		GVT3 map			HDC map		
Chromosome	No. loci	Genetic distance (cM)	Density (marker/cM)	No. loci	Genetic distance (cM)	Density (marker/cM)	No. gaps >10 cM
1	95	129.4	0.73	200	152.4	1.31	3
2	119	111.6	1.07	240	134.0	1.79	
3	145	124.4	1.17	269	159.4	1.69	1
4	98	100.4	0.98	248	110.7	2.24	2
5	194	141.5	1.37	479	141.4	3.39	1
6	135	119.3	1.13	284	154.6	1.84	
7	140	135.7	1.03	320	168.7	1.90	
8	142	154.2	0.92	334	191.0	1.75	1
9	155	139.3	1.11	336	146.1	2.30	
10	130	131.3	0.99	327	184.2	1.78	
11	206	220.2	0.94	404	228.2	1.77	
12	156	115.4	1.35	376	119.2	3.15	
13	140	131.4	1.07	356	131.4	2.71	
14	137	128.8	1.06	338	133.1	2.54	
15	136	137.4	0.99	324	230.8	1.40	2
16	95	139.6	0.68	278	139.6	1.99	
17	78	110.4	0.71	209	132.1	1.58	
18	119	113.2	1.05	303	121.0	2.50	
19	159	187.0	1.18	447	189.9	2.35	
20	127	156.5	0.81	332	168.1	1.98	2
21	139	158.4	0.88	411	173.9	2.36	
22	91	101.8	0.89	247	108.2	2.28	
23	121	123.1	0.98	304	170.9	1.78	1
24	116	137.2	0.85	326	173.9	1.87	2
25	109	149.4	0.73	284	154.5	1.84	
26	92	140.9	0.65	278	152.7	1.82	
**Total**	**3,374**	**3,538**	**0.95**	**8,254**	**4,069.7**	**2.03**	**15**

#### Integration of GV and T3 maps

The two selected “first order” component maps, GV and T3, showed overall excellent congruence in loci order. Among the 258 bridge loci, around 245 (95%) were collinear (Figure S1), representing on average 10 bridges per chromosome and thus permitting the map integration. Larger size differences for some chromosomes (c10, c11, c20 longer on GV; c5 and c17 longer on T3) between the GV and T3 maps (Figure 2A) were explained by the fact that some chromosome segments were not equally covered on the 2 maps, rather by a variation in the average distance ratio within a chromosome which was not significantly different (not shown). The intermediate consensus GVT3 map spanned 3,538 cM (3,637 and 3,380 cM for GV and T3, respectively) with 3,374 loci (1,745 and 2,072, respectively). Most chromosomes of GVT3 were intermediate in length between the two component maps. The number of unique loci in GV and T3 (not present in any of the 4 other map data sets) were 778 and 677, respectively, representing essentially AFLP markers for the GV map and a combination of SNPs (T3 was the only component map with SNPs) and SSRs of a specific origin (MON project) for the T3 map.

#### Integration of the 4 remaining component maps

The number of collinear bridge loci between each of the 4 remaining component maps and the GVT3 map was generally sufficient to allow map integration (Figure 3). Some map data were not considered because of too many inversions. These included a complete chromosome (c1 from the E3 map and c3 from the TH map) or a chromosome section (loci above 45 cM on c5 of the E3 map, below 78 cM on c18 of the TH and below 85 cM on c26 of the E3 map) (not shown). Apart from the above-mentioned cases, we also had to omit per chromosome around 10 loci in conflicting order, from either one of the 4 remaining component maps, in order to make the projection process possible. Respective contribution of the 4 component maps in terms of the locus enrichment (incremental addition of new unique loci) is illustrated in Figure S2. The figure illustrates a 4.3 cM interval on c24 containing 6 loci in GVT3 and progressively enriched to a final number of 14 loci in the HDC map.

The final HDC map, after the addition of unique loci from PK (1,352 loci), TH (796 loci), E3 (480 loci), and CH (33 loci), consisted of 8,254 loci (an increase of 4,880 loci, or 145%, compared to the intermediate GVT3 map). It spanned 4,070 cM (Table 3), or a 15% increase over the GVT3 map. Tabulated data for the consensus HDC map is available from the Table S3, and graphical display is presented in Figure S3 and also available through the CMap tool from TropGene database (http://tropgenedb.cirad.fr/tropgene/JSP/index.jsp). Due to a high proportion of unique (RFLP) loci, the PK map was the only map contributing to a few terminal chromosome segments in the HDC map (Table S3), including the upper 30 cM of c1 (covered with 13 markers), the upper 22 cM of c7 (with 10 markers), the upper 20 cM of c15 (with 10 markers), and the bottom 60 cM of c15 (with 17 markers). These segments in the HDC map may be considered with caution.

Individual chromosomes in the HDC map showed a more than 2-fold variation in size (largest for c15 with 231 cM and c11 with 228 cM, and shortest for c4 and c22, with 111 cM and 108 cM, respectively), as well as loci number (largest for c5 with 479 loci and smallest for c1 with 200). Chromosomes of the A_T_ (c1–c13) and D_T_ (c14–c26) subgenomes represented similar cumulative map distances (2,021 cM and 2,049 cM) and numbers of loci (4,173 and 4,081, respectively). Average marker density on the HDC map reached 2 loci per cM (one marker every 0.5 cM), highest for c5 and c12 (3.4 and 3.2 loci per cM, respectively), and lowest for c1 and c15 (1.4 loci per cM) (Table 3).

**Figure 4:**
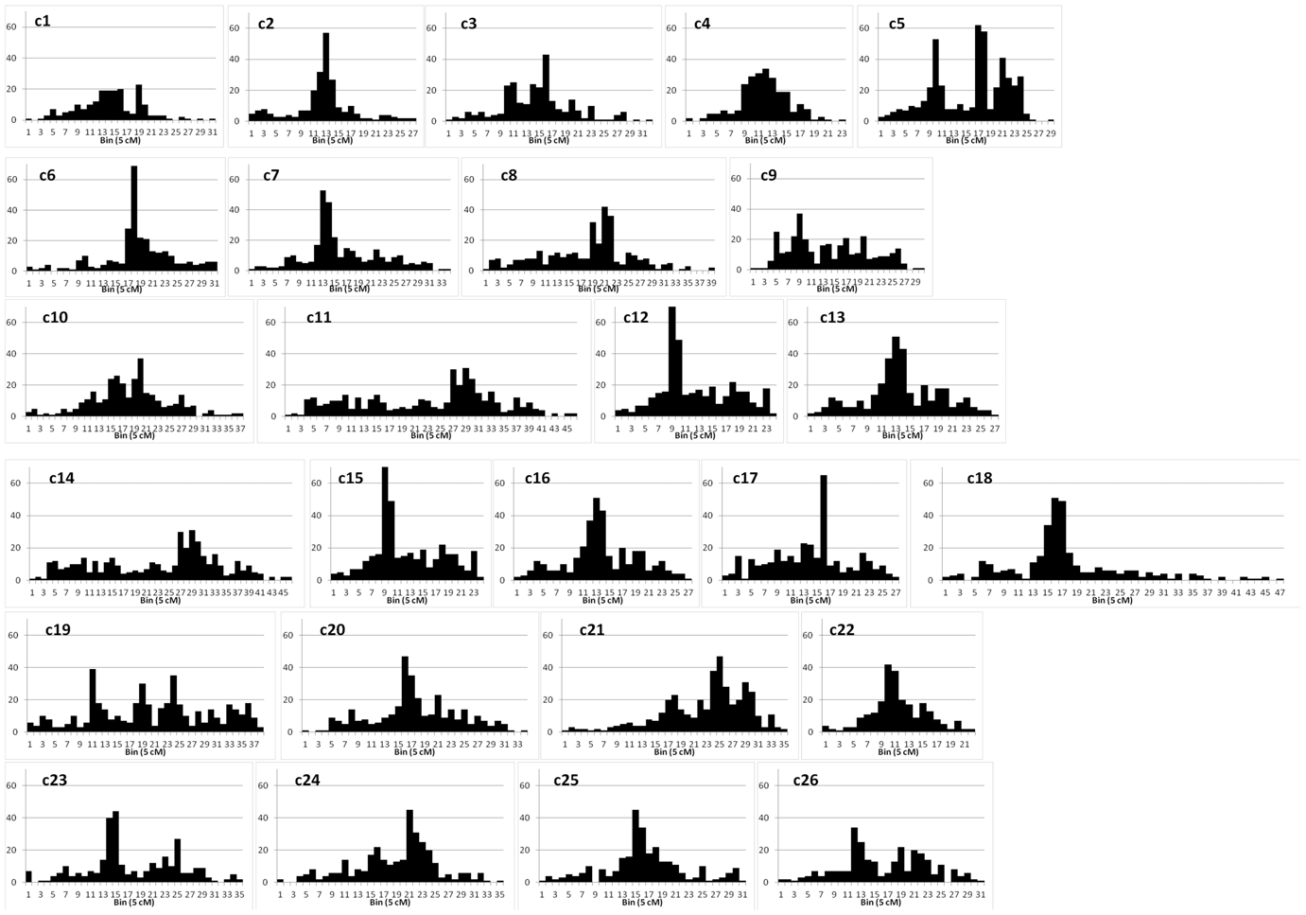
Distribution of marker density on the HDC map. The number of loci were counted in bins of length 5 cM.

**Figure 5:**
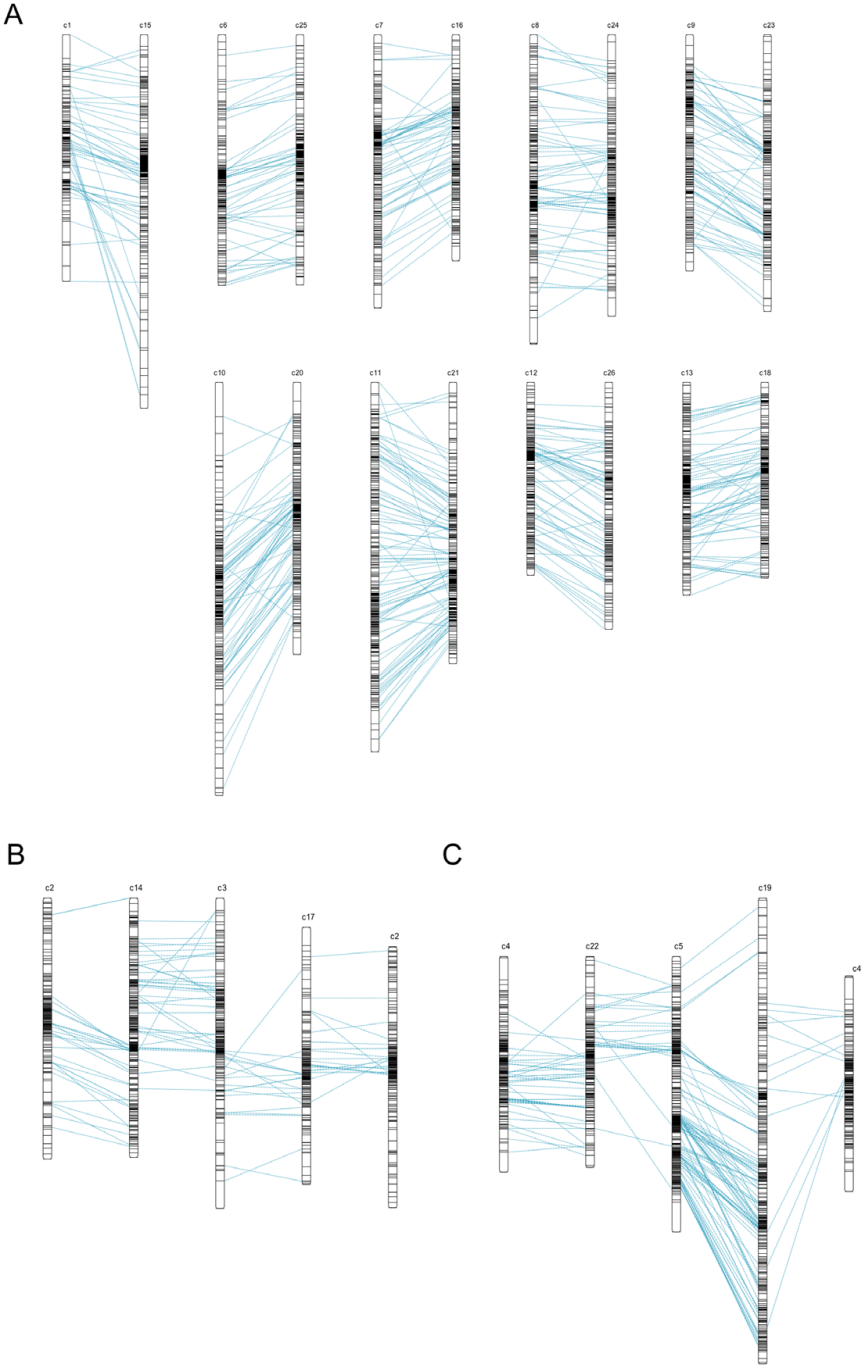
Marker duplication between homoeologous chromosomes of the HDC map. Duplications are represented (A) for 9 pairs of homoeologous pairs, (B) for groups of chromosomes c2–c14/c3–c17 and (C) for c4–c22/c5–c19. Cases B and C are illustrative of the reciprocal translocations between two A_T_ chromosomes, i.e. c2/c3 (case B) and c4/c5 (case C).

The distribution of loci along chromosomes was clearly uneven (Figure 4). Regions of higher density inferring presence of centromeric regions, were clearly delineated as a single region on most chromosomes. Their positions fairly corresponded between the homoeologs (also visible, as darker regions along chromosome bars in Figure 5 showing homoeologous duplications). In some cases it was not possible to clearly isolate a single hotspot, like for the 2 pairs of homoeologs c5 and c19, and c9 and c23 (Figure 4). There were 15 gaps larger than 10 cM, with the largest gap being 15.6 cM. All of those gaps were within or close to distal parts of chromosomes (among the first three or the last three loci). Although existence of regions of lower marker density is not unexpected (the lower density areas may indicate the homozygosity (fixation) between *Gh* and *Gb*), they may also be caused by possible misplacement of some unique loci and thus may be artifacts. Examination of the raw data in the mapping populations (not considered in this study due to unavailability of original mapping data for each of the component maps) would have possibly allowed pointing out the missing data, known to cause artificial inflation of map distances.

The 8,254 loci of the HDC map originated from 6,669 different (non-redundant) markers, including 3,450 SSRs (of those 727 represented by a CLU collective name), 1,894 RFLPs, 770 AFLPs, 205 SNPs; the others representing 3 morpholological markers (linter color locus, noted LTCOL, on c21, petal color locus *P1* on c5, and petal spot locus *R2* on c7), various gene markers of proprietary origin (73) and markers of unknown origin (274). Among all the markers on the HDC map, 71% (4,744/6,669) had sequence information and originated either from genomic sequences, such as SSRs derived from enriched libraries (series BNL, CIR, etc..) or from the expressed sequences, such as cDNA-derived RFLPs and EST-SSRs (Table S4). Among the 6,669 markers, 5,377 mapped to a single locus and 1,292 were multi-copy markers mapped to multiple positions (between 2 positions and a maximum of 9 positions for MUSB0641). Multi-copy markers bridged either two homoeologous A_T_ and D_T_ chromosomes (64% of all duplications), two non-homoeologous chromosomes from the same or from the 2 subgenomes (22%), or lastly represented paralogous duplications on the same chromosome (14%). Figure S4 presents the pattern of all the duplications over the 26 chromosomes of the HDC map.

### Homology of the HDC genetic map with the D genome

**Figure 6:**
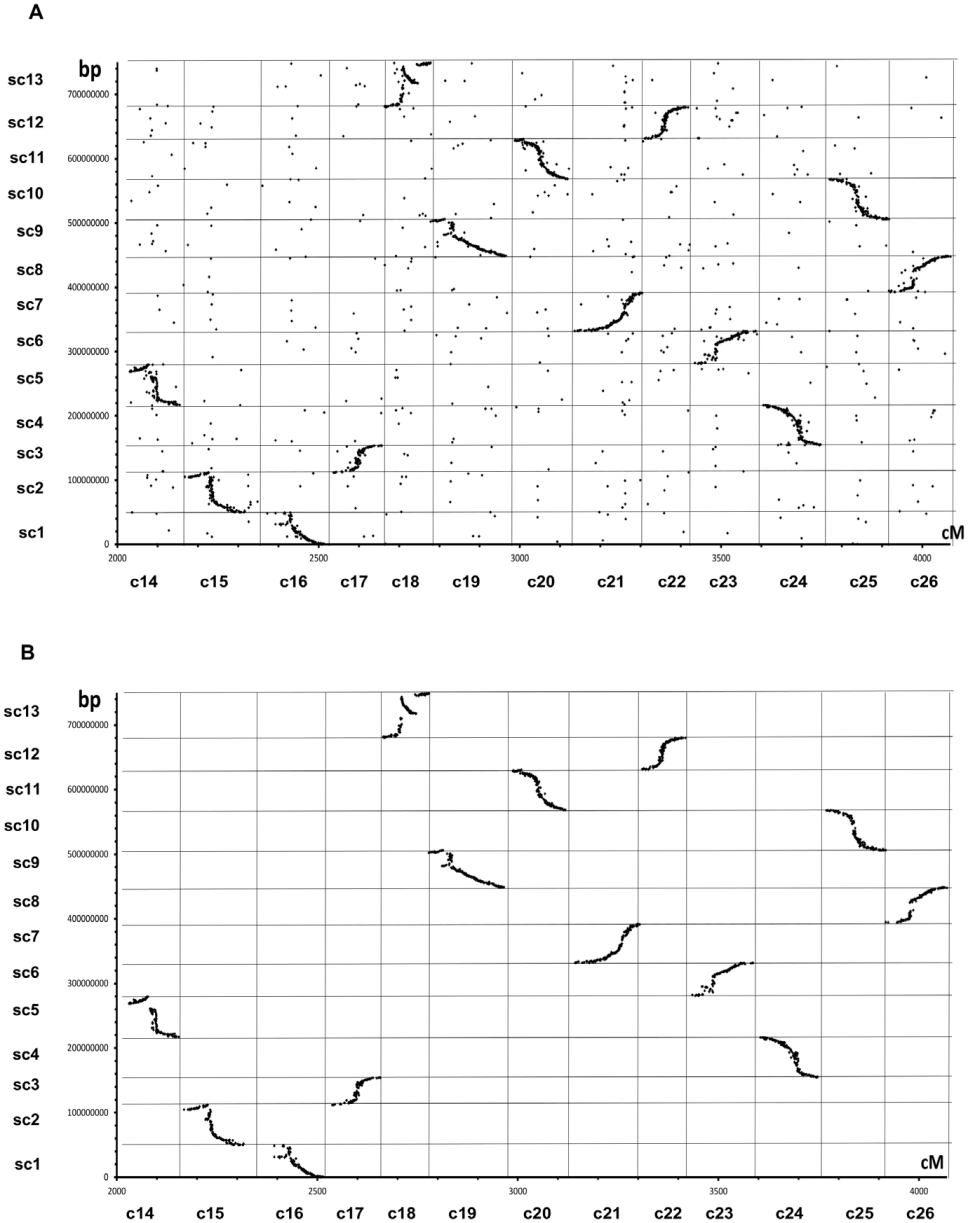
Relationship between marker order on the HDC map and physical distance for the 13 chromosomes or scaffolds of the D genome of *G. raimondii*. The D chromosomes of the consensus map correspond to 2,049 cM of chromosomes 14 through 26 (units in cumulated cM), and physical map corresponded to concatenated 750 Mb of the 13 first scaffolds (sc1 to sc13) of the *G. raimondii* genome (units in cumulated bp). The correspondences were derived from sequence-based mutual best hits homology (BBMH) between marker and genome scaffolds. A. All BBMH results (include 2,377 points). B. BBMH followed by clustering represented at least by 5 gene-pairs (include 1,780 points).

The search for matches between the mapped markers with available sequences and the 13 major scaffolds of the *G. raimondii* genome using Best Blast Mutual Hits (BBMH) generated 4,590 hit pairs (3,606 different markers, due to duplications in the HDC map). The number of hits was reduced to 2,377 (2,189 different markers) after considering only the markers mapped on D_T_ chromosomes of the genetic map. The other 2,213 markers specific to A_T_ chromosomes were not considered. Figure 6A presents for the 2,377 hits the relationship between marker position on the HDC map and physical position along genome scaffolds. This number of hits was further reduced to 1,780 after clustering (average of 137 hits per chromosome among the 13 D_T_ chromosomes) (Figure 6B). The clustering eliminated all single outlier hits. Forty six clusters contained between 6 and 164 hit pairs.

Global orientation was reversed in 7 of the 13 chromosomes, because of incorrect long-to-short arm assignment. Interestingly, the order of markers in the HDC map remained essentially the same as that of their hits along scaffolds of the *G. raimondii* physical map; however, the S-shape aspect indicated that the relationship between genetic distances in cM and physical distances in base pairs varied greatly. Globally, the 750 Mb of the 13 scaffolds corresponded to 1,836 cM, or 409 kb per cM. Marker-dense regions of the genetic map (see Figures 4 and 5) inferring to correspond to less recombination-active peri-centromeric regions, had a high kilobase pair to centimorgan ratio, while the same ratio in distal (telomeric) regions was very low. In Arabidopsis, this ratio varied from 30 to 550 kb per cM for chromosome 4 [34]. In wheat [35], [36], the variation was even more extreme, with 1 cM corresponding to the range from 118 to 22,000 kb.

Some discrepancies (interruption and inversion of respective orders) occurred locally in some terminal segments, such as c14 (scaffold 5) and c15 (scaffold 2). In the case of c18 (scaffold 13) an inverted segment representing nearly one third of the overall distance was located internally (Figure 6).

## Discussion

### Redundancy in cotton marker databases

Tetraploid cotton was derived ∼1–2 million years ago from a naturally occurring cross between two diploid genomes, A and D [37]. The two genomes have retained some level of sequence similarity, resulting in a high transferability of markers among the *Gossypium* spp. [38], [39], as well as in the fact that, whenever both genomes are expressed in tetraploid, the two homoeolog cDNAs are co-assembled in EST assemblies [40] (Lacape et al, submitted). The presence of homoeolog A_T_ and D_T_ copies translates into the existence of multi-copy banding patterns for the markers like RFLPs and SSRs, and the possibility for a single marker to segregate for the 2 homoeologous loci, as frequently reported [14], [15], [18].

Since the mid-1990s, cotton SSRs had been developed either from enriched libraries of the genomic DNA or from cDNA (EST) collections. The majority of the libraries originated from accessions of the polyploid species *Gh*. As the number of published cotton markers increased, reaching nearly twenty thousands, some marker redundancy had been noticed by molecular geneticists, but no serious effort had been made to clarify this situation. Redundancy was expected not only because of the fact that the same resource (genome, ESTs) had been browsed by different groups using different filters (parameters), but also because of the existence of real sequence copies in the cotton genome, with the most obvious type of copies being represented by homoeologs.

The CMD database of cotton SSR markers comprised 17,343 identifiers characterized by a sequence (as of May 2012) shared among 18 major series (Table S2). Following sequence-based redundancy using a fairly stringent similarity threshold (90%), it was found that at least 3,539 markers were to be considered as redundants, while 2,357 unique sequence clusters were sufficient to represent 5,896 markers (Table S1). Of these 5,896 markers, 4,416 (75%) were derived from EST DNA libraries. It is expected that our sequence-based marker redundancy check partly identified homoeologs (identical by state) as being redundant, although the two sequences in this particular case may be considered as different. Imposing a similarity threshold higher than 90% would have possibly separated the copies, but the aim of our marker redundancy curation was related to the use of these markers in genetic mapping.

### Component maps and their roles in HDC map construction

The markers redundancy information had been integral part of the map integration process. We based our map integration on an empirical approach implying curation of each individual component map for the presence of artificial duplications derived from un-intentional mapping of redundant markers, as well as on the comparison of component maps based on the shared loci to solve conflicts in the loci order. Component maps were ranked qualitatively relative to their intrinsic value (rate of redundant and unique markers) and mutual informativeness (connections with other maps and rate of loci order conflicts).

The existence of redundant markers mapped un-intentionally twice in the same mapping population also served as an indicator of the relative quality of the mapping data (i.e. the shorter the distance between the two redundant markers, the better). Average distances between loci from redundant markers mapped as duplicates in paralogy on each map (same marker on the same chromosome) were 1.7 cM for T3 (72 pairs of loci), 2.1 cM for GV (21 pairs), 2.2 cM for CH (16 pairs), 2.6 cM for TH (40 pairs), and 2.8 cM for E3 (125 pairs). The PK map was not amenable to this calculation, as it had only few SSR loci. The two recently published maps, GV and T3, were ranked as the “first order” maps, because the direct access to the original mapping data allowed the re-computation of the maps and slight local revisions of some chromosomes (not shown). In addition, these two maps had minimum number of loci order conflicts with other maps (not shown). The four remaining “second order” component maps were then iteratively projected in the map order PK, TH, E3, and CH.

The HDC map is enriched with sequenced markers such as RFLPs, SSRs, and SNPs. These markers collectively represent 4,744 unique sequences that we used further to compare genetic and physical maps. As compared to the existing mapping data, the HDC map (8,254 loci) has a considerable increase in the number of loci. The density of loci (2 loci per cM on average) has been increased more than three folds as compared to the most dense component map, PK [18].

The HDC map represents the second comprehensive effort of map integration in cotton. The comparison of our map with the earlier reported Comprehensive Reference Map, CRM, by Yu et al. [33] (7,242 markers, 14,868 loci, no distance calculated), was however difficult for different reasons. The CRM map did not include a marker redundancy check, meaning that the number of connections with our map was over-estimated and generated a number of artefactual inversions. Secondly the approach followed for the construction of CRM was not aimed at calculating distances, meaning again that local discrepancies in locus order between HDC and CRM would have been merely impossible to assess qualitatively.

### Loci duplications in the HDC map

The HDC map also has considerably increased the number of duplicate markers bridging homoeologous chromosomes in the tetraploid cotton map: 1,660 loci (830 connectors) are duplicated among all 13 homoeologous pairs on the HDC map (Table 4 and Figure S4). This count took into consideration the reciprocal translocations involving 2 pairs of A_T_ chromosomes, c2/c3 and c4/c5 (Table 4). These two previously described reciprocal translocations involving c2/c3 and c4/c5 as illustrated in Figure 5B and 5C (Venn diagram of shared loci in Figure S5) are shown by the cross duplications between a single A_T_ chromosome and 2 chromosomes of the D_T_ genome. For example, duplications of c2 are pointing towards both c14 for the bottom of c2 and c17 for the upper part of c2 (Figure 5B). However, the exact position of translocation breakpoints on c2/c3 and c4/c5 remains ambiguous due to the fact that the groups of cross-duplications are overlapping (Figure 5B and 5C, respectively). It may be assumed that those translocations involved complete chromosome arms, and that breakpoints should be located in the vicinity of centromeres. This remains to be verified when centromere-specific markers will be positioned physically on the genome, such as Ty3-gypsy-like LTR retroelement [41].

**Table 4:** Summary data of marker duplications between chromosomes on the HDC map.

Chromo-some	1	2	3	4	5	6	7	8	9	10	11	12	13	14	15	16	17	18	19	20	21	22	23	24	25	26
**1**	5	2	2	2	3	1		4	3	6	1	1	2	2	**56** 1)	3	4	4	5	3	3	4	1	2		
**2**		1	1	3	3	1		8	1	4	4	1	2	**31**	4	1	**20**		2	2	1	1	1	2	2	1
**3**			4	1	3		6	1	1	2	2	6	8	**45**		1	**21**	5	5	4	3	1	2	5	1	3
**4**				5	7	5	2	7	3	2	4	7	3	3	1	2		2	**12**	3	5	**46**	4	6	1	4
**5**					9	6	4	4	2	3	6	4	4	3		1	2	4	**89**	4	4	**29**	3	4	10	2
**6**						2	1	4	6	4	3	3	7	1		1	2		3	1		1	2		**49**	1
**7**							8	4	2	4	8	3	6	2	3	**60**	1	2	3	2	7	5	3	1	3	5
**8**								6	4	6	4	1	9		2	4	1	5	1	4	1	2		**63**	2	
**9**									8	6		5	7	2	2	2		1	2	1	6	7	**67**	4	1	2
**10**										6	1	2	4	3		2		5	2	**64**	3	2	1	3	5	2
**11**											13	5	5	2	1	2	4		5	6	**105**	1	3	4	8	6
**12**												6	7	3	2	3	3	6	5	2	2	4	1	1	3	**64**
**13**													2	1	4	5	3	**75**	2	6	7	4	3		1	2
**14**														10	3	3	4	5	2	3	5	5	4	1	1	1
**15**															8	7	4		3	2	8	5		2		4
**16**																6	2	2	4	1	4	2	3	4	2	3
**17**																	5	2	3	1	3	3	1	1	3	1
**18**																		9	7	5	2	2	3	1	1	2
**19**																			7	1	5	7	3	3	8	1
**20**																				6	8	2	1		3	2
**21**																					11	6	4	11	4	4
**22**																						7	6	2	4	5
**23**																							9	8	4	
**24**																								12	3	1
**25**																									10	3
**26**																										5

1) Counts involving homoeologous chromosomes are indicated in bold (c1–c13 from A_T_ subgenome vs. c14–c26 from D_T_ subgenome).

In most cases the linear order of loci along homoeologous chromosomes (or chromosome arms in the cases of c2/c3–c14/c17 and c4/c5–c19/c22) was conserved, as illustrated in Figure 5 by the abundance of connections fairly parallel relative to the isolated cases of conflicting connections. Such collinearity along virtually all chromosomes was also prevalent in *Brassica* genomes [42]. The conflicting connections may be interpreted as mapping errors, as remnant paralogous duplications, or else as signs of structural re-arrangements.

An exception to the conservation in loci order between homoeologous chromosomes was observed in pair c1–c15 (Figure 5A). A group of loci of c1 duplicated on c15 was not respecting global collinearity and apparently delineated a region at the bottom of c15 that may have undergone structural rearrangement, i.e. proximal duplication (between 150 and 200 cM) accompanied by an inversion (Figure 5A). However, the bottom 60 cM of c15 (also being the longest chromosome) in the HDC map was covered with 17 markers unique to PK map (Table S3), suggesting that the deviation from collinearity between c1 and c15 in this region, may also be interpreted by a mapping discrepancy in the component map PK.

Besides duplications between homoeologous chromosomes, the HDC map also documented a series of duplications between non-homoeologous chromosomes of the 2 subgenomes as well as within the same subgenome (intra-A_T_ and intra-D_T_) (Table 4). The intra-subgenomic duplications have been suggested as supporting the hypothesis of an ancient chromosomal duplication (paleo-polyploidization) predating divergence of modern *Gossypium* diploid genomes, an ancestor of 6 or 7 chromosomes giving rise to the 13 chromosomes number of diploids [18]. Such inference was made in Rong et al. [18] for example in the case of c7 and c11 (see their Figure 7 with c11 named A03). In the HDC map, these intra-subgenomic duplications seem to be scattered over many chromosomes (Figure S4), as a given chromosome had duplicates with at least 22 different other chromosomes, and when duplicates are in sufficient number (such as for c7–c11, c8–c13, or c21–c24) they do not show collinearity (Figure S4), thus providing only limited support to the hypothesis for an ancient polyploidization.

Lastly, all chromosomes of the HDC map presented intra-chromosomal duplications (paralogous) (diagonal in Table 4). The total number of paralogous markers reached 182 (364 loci), with the highest numbers for c11 (13 loci), c24 (12), c21 (11) and c14 and c25 (10). Although some groups of loci are in collinear order (Figure S4) the arrangements of these duplications are not providing clear evidence of proximal duplications or of relics of structural rearrangements.

### Collinearity of the HDC map with the D genome

The overall marker order on the HDC map agreed well with the order of corresponding homolog sequences on the 13 major scaffolds of the *G. raimondii* genome (Figure 6). This result is paving the way for many important applications, such as multi-data integration, projection of QTL and eQTL data, QTL fine mapping, identification of candidate genes, map-based gene cloning, translational genomics, etc.

Local discrepancies in the orders along scaffolds and linkage groups were located in a few regions (in particular c14, c15, and c18 for the most obvious cases), and may be interpreted either as errors in the construction of the consensus map (although locus order was corroborated by several if not all 6 component maps) or in the genome assembly, or else may be indicative of some structural re-arrangements between homologous chromosomes in the diploid (*G. raimondii*) and in the tetraploid context (13 D_T_ chromosomes of the tetraploid genome genetic map).

Earlier studies by comparative linkage mapping between diploid and tetraploid genetic maps involved, as diploid genomes, species of the A genome (*G. herbaceum*×*G. arboreum*) in Desai et al. [25] and of the D genome (*G. trilobum*×*G. raimondii*) in Rong et al. [18]. In the case of the A genome (*viz.* diploid A and tetraploid A_T_), 2 translocations, corresponding to the well described pairs c2/c3 and c4/c5, and 7 inversions were observed between the A and the A_T_ genome [25], while in the case of the D genome (*viz.* diploid D and tetraploid D_T_) Rong et al. [18] reported a virtual collinearity between all D and D_T_ chromosomes.

## Conclusion

In conclusion, the high density consensus, HDC map is a highly valuable resource for different applications, including the integration of new map data from the same populations (such as by D Fang for new SSR markers and by JM Lacape for new SNP markers, papers submitted) and from different mapping populations, both inter- or intra-specific (a saturated intra-specific *G. hirsutum* map is still lacking); the optimized choice of panels of well distributed markers for construction and assembly of new maps, association mapping in cotton (facilitating estimation of linkage disequilibrium), or fine-mapping around chromosomal regions of interest; integration of QTL map data (e.g. project QTL LOD peaks and QTL confidence intervals) as many of the component maps hereby used to construct the HDC map have also served for mapping QTLs for phenotypic traits [43], [44] or for gene expression QTLs, eQTL [45].

## Materials and Methods

### Marker redundancy curation

#### Sequence comparison of SSR primers and SSR-containing sequences

As a preliminary step in the marker redundancy curation, we performed the sequence alignment of the available SSR primers; 17,448 SSRs with both forward and reverse primers were used from the CMD database (http://www.cottonmarker.org/). For each SSR, its forward and reverse primers were checked for redundancy against the forward and reverse primers of all the other SSRs using local pairwise alignment based on the Smith-Waterman algorithm [46]. The parameters for the alignment were set as following: match -7, replace 7, insert 7, delete 7, gapExtend 14, and NUC.4.4 matrix was used as a substitution matrix. As a cutoff, we used the 90% similarity for a short primer sequence, but results will be reported at the 95% and 100% similarity cutoff values. Full SSR-containing sequences were then searched for sequence similarity. In total, 17,343 SSR-containing sequences were checked for redundancy using local pairwise alignment based on the same algorithm and following parameters: match -1, replace 3, insert 2, delete 2, gapExtend 4, and NUC.4.4 matrix was used as substitution matrix and 80% sequence similarity cutoff.

#### Additional marker redundancy curation using map data

Whenever map data were available for a pair of suspected (sequence-based) redundant markers, it was used as an additional evidence confirming or infirming marker redundancy, depending on the chromosome localization of the 2 suspected redundant markers (i.e., occurrence (or lack of it) of 2 loci on the same chromosome in the corresponding population). The integration of available map information helped us to increase the sequence similarity threshold to a cutoff value above 80%, which would minimize the number of false positives. Following the sequence-based check of redundancy among SSR markers, the clusters of markers identical by sequence were assigned a common name (all markers are further referred-to with ‘CLU’ acronym). In numerous cases it was observed that redundant markers had been non-intentionally mapped twice on the same map or on 2 different maps, and consequently mapped at the same or very close positions (though expected to map at exact similar position), implying the need for curation of fortuitious duplications.

### Component genetic maps used for the consensus map construction

Among the different published genetic maps, we chose six interspecific *Gh*×*Gb* maps [8]–[10], [13], [14], [18], based on the fact that they were saturated (26 linkage groups), provided the highest density of loci, and offered a sufficient level of connecting bridge markers in a collinear order.

The following names (CH, E3, GV, T3, TH, and PK) were given to the six maps used in the consensus map construction (summary data are given in Table 1). **CH** **-** the linkage map from a cross (*Gh* CRI 36×*Gb* Hai 7124) using F_2_ plants and consisting of SRAP, TRAP, AFLP, and SSR loci) [10]. **E3** **-** the map based on population of BC_1_ plants derived from a cross [(*Gh* Emian 22×*Gb* 3–79)×*Gh* Emian 22] and containing exclusively SSR markers [13]. **GV** **-** the consensus genetic map integrating loci (AFLP, RFLP and SSR) from 2 populations (BC_1_ and RIL) of a cross (*Gh* Guazuncho 2×*Gb* VH8-4602) [9]. **T3** **–** the recently reported SSR and SNP genetic map based on RILs derived from a cross (*Gh* TM-1×*Gb* 3–79) [14]. **TH** **-** the SSR map based on BC_1_ plants derived from a cross [*Gh* TM-1×*Gb* Hai 7124)×*Gh* TM-1] [12]. **PK** – the RFLP-based genetic map derived from a cross (*Gh* race palmeri and *Gb* acc. K101) [18]. Since the raw mapping data used for constructing the component maps were not available and would have been difficult to individually curate, we based our map integration process on the order and distances of loci along the linkage groups as previously published, as well as on the existence of bridges between the maps.

Some other interspecific maps, though saturated or nearly saturated [17], [47] were not considered for the consensus map construction. For example, Handan 208×Pima 90 map [47] had low genome coverage, high number of inversions with GV and T3 maps, as well as low rate of new markers. In the case of DP 33B×3–79 map [17], which was highly informative in terms of the presence of unique markers, no actual map distances were available, but rather positions along bins of 20 cM.

The selected 6 maps were individually verified for the standardized chromosome nomenclature, and the c1–c26 system was adopted. Marker nomenclature was also verified, since the same marker may have been reported under different locus names, including synonyms for the same marker, such as for example BNL119 or BNL0119. Previous reports assigned SSR markers to chromosomes and chromosome arms in tetraploid cotton using various cytogenetic deficient stocks [48], [49]. An arm orientation was thus possible for some chromosomes, and a short-arm (top) to long-arm (bottom) orientation was usually preferred. Since parallel loci arrangement on homoeologous A/D chromosomes was expected, an agreement in orientation between the 2 homoeologous chromosomes was given priority whenever a contradiction existed between the homoeologs, and chromosomes on the component map were oriented accordingly.

### Consensus map construction

The existence of redundancy among the markers was considered during the identification of bridges between the component maps, e.g. collective CLU naming was used, thus improving map connectivity (integration). When 2 redundant markers on 2 different genetic maps were located on the same chromosome, this pair of markers would constitute an additional bridge between the 2 maps. For example, NAU2083 and NAU3104 were redundant markers (96% sequence similarity), assigned the same name (CLU1349); NAU2083 was mapped on c1 from map T3, and NAU3104 was mapped on c1 from map TH, thus both markers created a new bridge between the 2 maps, T3 and TH.

The construction of the consensus map was possible due to the presence of a sufficient number of loci bridging the component maps in a collinear order. The “neighbours” approach was applied [50], where the position of a unique locus was extrapolated from the positions of its 2 closest shared flanking loci. We used the Biomercator V3 software to perform the map integration [51]. Compared to its earlier versions, Biomercator V3 includes several new map compilation and meta-analysis algorithms from the MetaQTL package [52]. Regarding the consensus map construction, Biomercator V3 proposes two compilation algorithms, *iterative compilation*, or map to map homothetic projection; and *regression loci compilation*, an algorithm formerly implemented in MetaQTL and based on a Weighted Least Square strategy.

The selected 6 maps were merged in 2 steps, by considering (i) the two maps, GV and T3, as the maps of higher confidence for integration, and then (ii) the 4 other maps, PK, TH, E3, and CH, to be projected iteratively. The GV and T3 maps were integrated by the *regression loci compilation* with no reference, i.e. with recalculation of distances, and the resulting map was coded GVT3. The maps PK, TH, E3, and CH were then iteratively projected (*iterative compilation*) one after another in the following order: PK was projected on the consensus GVT3 map used as a reference, then TH was projected on the GVT3+PK map used as a reference, and so on. During the second step, the distances remained unchanged compared to the distances on the GVT3 map.

Positions of distal unique loci were computed by Biomercator from the global distance ratio of all shared intervals. During all the steps, map integration implied that all inversions in the order of bridge markers between the 2 given maps had to be examined and resolved. The majority of inversions occurred within short map distances (<10 cM) and were attributed to mapping errors. In such cases, the locus to-be-projected was simply discarded, and its counterpart locus on the reference map was kept. Inversions over longer distances (≥10 cM) were interpreted as indicative of possible paralogous duplications. In such cases, both loci we conserved and one of the two was temporarily renamed in order to be excluded as a bridge. Such paralogous duplications were present within a single map, or between pairs of maps. All pairwise map combinations were visually checked before compilation to resolve as many as possible of the cases of inverted loci. In case of a missed and unresolved inversion, the 2 loci would be discarded by Biomercator.

### Sequence homology between markers and *G. raimondii* genome

Following the recent release of a draft sequence assembly (not annotated) of the diploid D genome species *G. raimondii* (http://www.phytozome.net/cotton.php), we downloaded the sequence fasta files of the 13 first larger scaffolds (13 chromosomes) representing more than 750 Mb. The sequences of the 4,744 different markers mapped on the HDC map were aligned to the scaffold sequences using BLASTN algorithm with an e-value cutoff of 1e-5. In order to decrease the number of non-specific matches between the two set of sequences (mainly due to repetitive sequences), a Best Bidirectional Mutual Hit approach (BBMH) was used to filter the BLASTN results. For 2 given sequences, A (scaffold) and B (marker), the BLAST-Hit was considered if A was the best match for B and B was the best match for A. Among the 4,744 marker sequences, 3,663 gave a unique BBMH hit. Following the dot-plot representation of scaffold base pair units versus cM units on the D genome chromosomes of the consensus map (chromosomes of the A genome were not considered), a single linkage clustering with an euclidian distance (based on the index order in each scaffolds) was then used to build clusters of collinear sequence pairs between the genetic map and the sequence assembly. In-house perl scripts were used to build clusters containing at least 5 collinear gene pairs, and a new dot-plot representation was built excluding the non-clustered hits.

## Supporting Information

Figure S1 **Collinearity of locus order between GV and T3 maps and integration of these 2 maps into consensus GVT3 map.**
(TIF)

Figure S2 **Construction of HDC map. Chromosome 24 shown as an example.** Two-stage process for map integration of c24 and progressive enrichment of markers: 1^st^ stage in upper panel showing T3, GV and GVT3 connections and a zoom over interval 61.3–65.6 cM, or 4.3 cM, between CIR061-DPL0534 (4 loci, 4.3 cM); and 2^nd^ stage in lower panel with same interval enriched iteratively with 2 loci from PK, 4 loci from TH, 1 locus from E3 and 1 locus from CH, for a final density of 14 loci in the same interval distance.
(TIF)

Figure S3 **The HDC genetic map of tetraploid cotton.** Bridge markers (mapped in more than one component map) are highlighted in red (actual marker name) or in green (cluster of markers). Chromosomes of the A subgenome, c1 through c13 are presented in Figure S3A and of the D subgenome, c14 through c26, are presented in Figure S3B.
(PDF)

Figure S4 **Dotplot showing duplications of loci in the HDC map.** Each dot represents a marker mapped multiple times in the HDC map. X- and Y- axes represent chromosomal locations (units in cumulated cM), with c1–c13 representing chromosomes of the A_T_ subgenome and c14–c26 chromosomes of the D_T_ subgenome
(TIF)

Figure S5 **Venn diagrams of the number of duplications among c2, c3, c14, and c17, and among c4, c5, c19, and c22, in the HDC map.**
(DOC)

Table S1 **The components of each marker cluster based on sequence redundancy check and map localization on HDC map.**
(XLS)

Table S2 **Number of sequence-based redundant (90% similarity threshold) marker pairs within and between major SSR libraries.**
(XLS)

Table S3 **The final HDC cotton genetic map constructed from six component maps.** For each marker locus of the HDC map (including redundant markers with “CLU” collective name), the original localization and name(s) on the corresponding component map(s) are also indicated.
(XLS)

Table S4 **Summary of molecular marker types, and identities, used in the construction of the HDC cotton map.**
(XLS)
