# Correction: A High Density Consensus Genetic Map of Tetraploid Cotton That Integrates Multiple Component Maps through Molecular Marker Redundancy Check

**DOI:** 10.1371/annotation/ab8211a0-53a5-4765-a7cb-4b613ffb1b09

**Published:** 2013-03-11

**Authors:** Anna Blenda, David D. Fang, Jean-François Rami, Olivier Garsmeur, Feng Luo, Jean-Marc Lacape

The authors wish to provide a small correction in the section about "convergence of the HDC map with the D genome". During the review and production process of our article, an updated version (v2.1) of the genome of Gossypium raimondii ( http://www.phytozome.net/), has been released which has "corrected several orientation issues within scaffolds" (as mentioned from phytozome website). A new BBMH-based BLAST of the markers of the HDC map against the genome of G raimondii was undertaken. This new comparison does not modify the total number of hits that was reported. However, the former Figure 6 should be disregarded. A corrected version of Figure 6 can be viewed here: 

**Figure:**
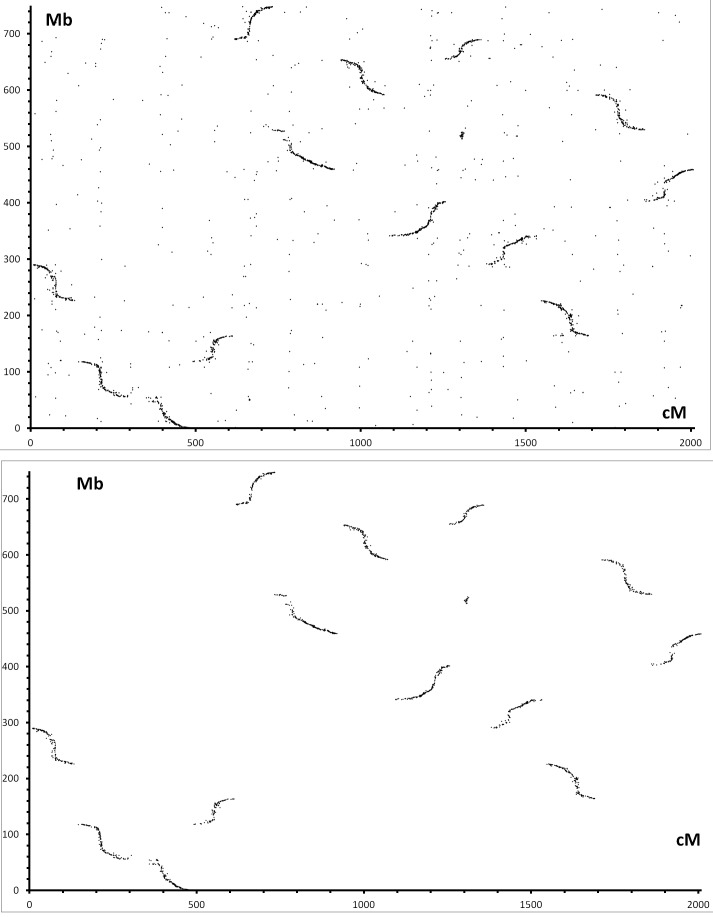


The authors wish then to simplify their comments on this aspect, and emphasize the overall excellent convergence (no real discrepancies unlike previously written) between the alignments of markers on the HDC map and their hits along the scaffolds of the genome of G raimondii. The authors apologize for any inconvenience this may cause. 

