# Correction: A High Density Consensus Genetic Map of Tetraploid Cotton That Integrates Multiple Component Maps through Molecular Marker Redundancy Check

**DOI:** 10.1371/annotation/d2b10915-daa3-4ed7-ad95-3a6d50b26fea

**Published:** 2014-01-22

**Authors:** Anna Blenda, David D. Fang, Jean-François Rami, Olivier Garsmeur, Feng Luo, Jean-Marc Lacape

There was an error in the Correction posted on March 11th, 2013. Please view the corrected Figure 6 here: http://www.plosone.org/corrections/pone.0045739.g006.1.cn.tif

