# Supplementary figures and images for: A High Density Consensus Genetic Map of Tetraploid Cotton That Integrates Multiple Component Maps through Molecular Marker Redundancy Check
Source: PLoS One. 2012 Sep 24;7(9):e45739. doi: 10.1371/journal.pone.0045739 (PMC3454346; doi:10.1371/journal.pone.0045739)

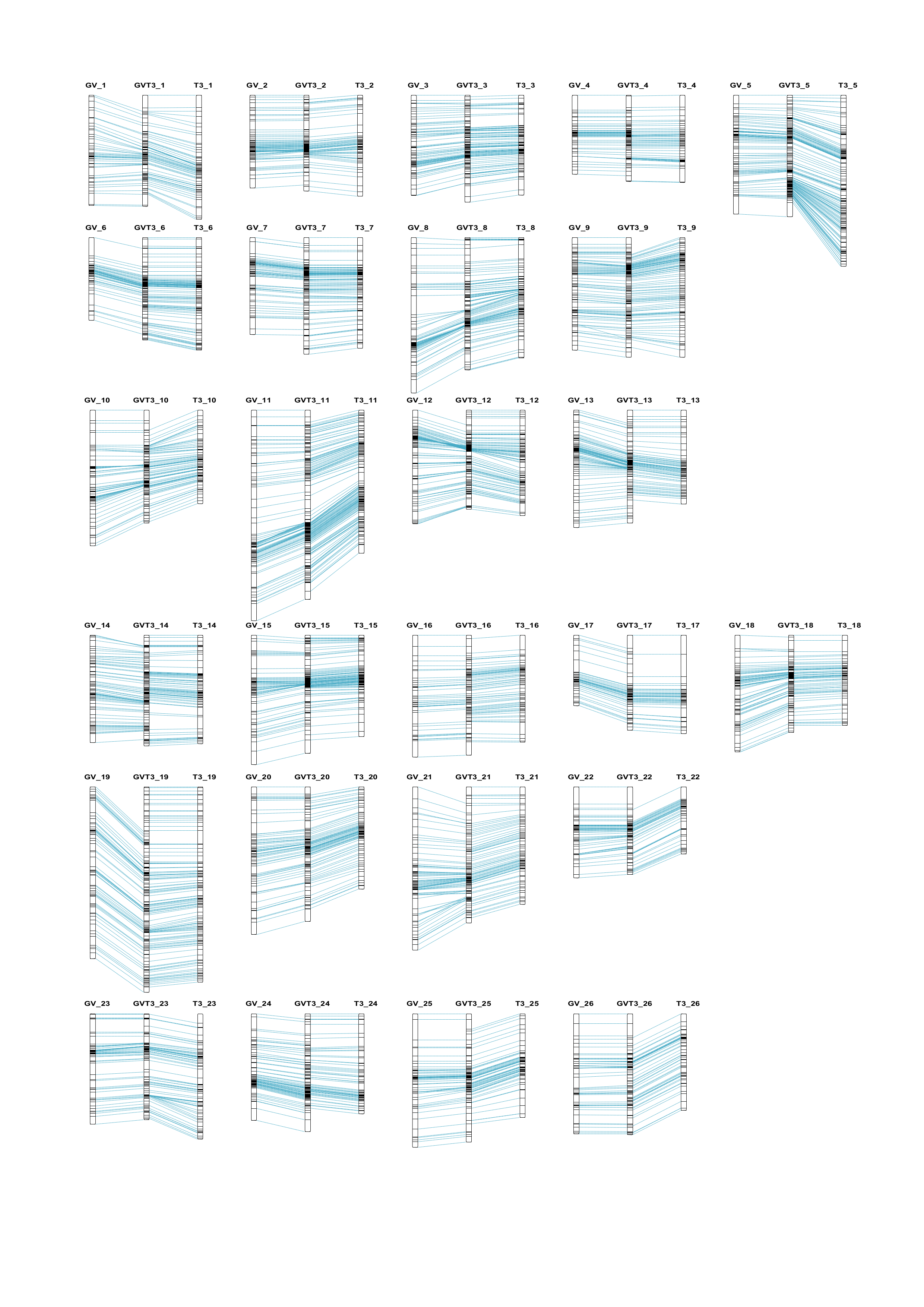

Supplement: Figure S1 — Collinearity of locus order between GV and T3 maps and integration of these 2 maps into consensus GVT3 map. (TIF) [file pone.0045739.s001.tif]

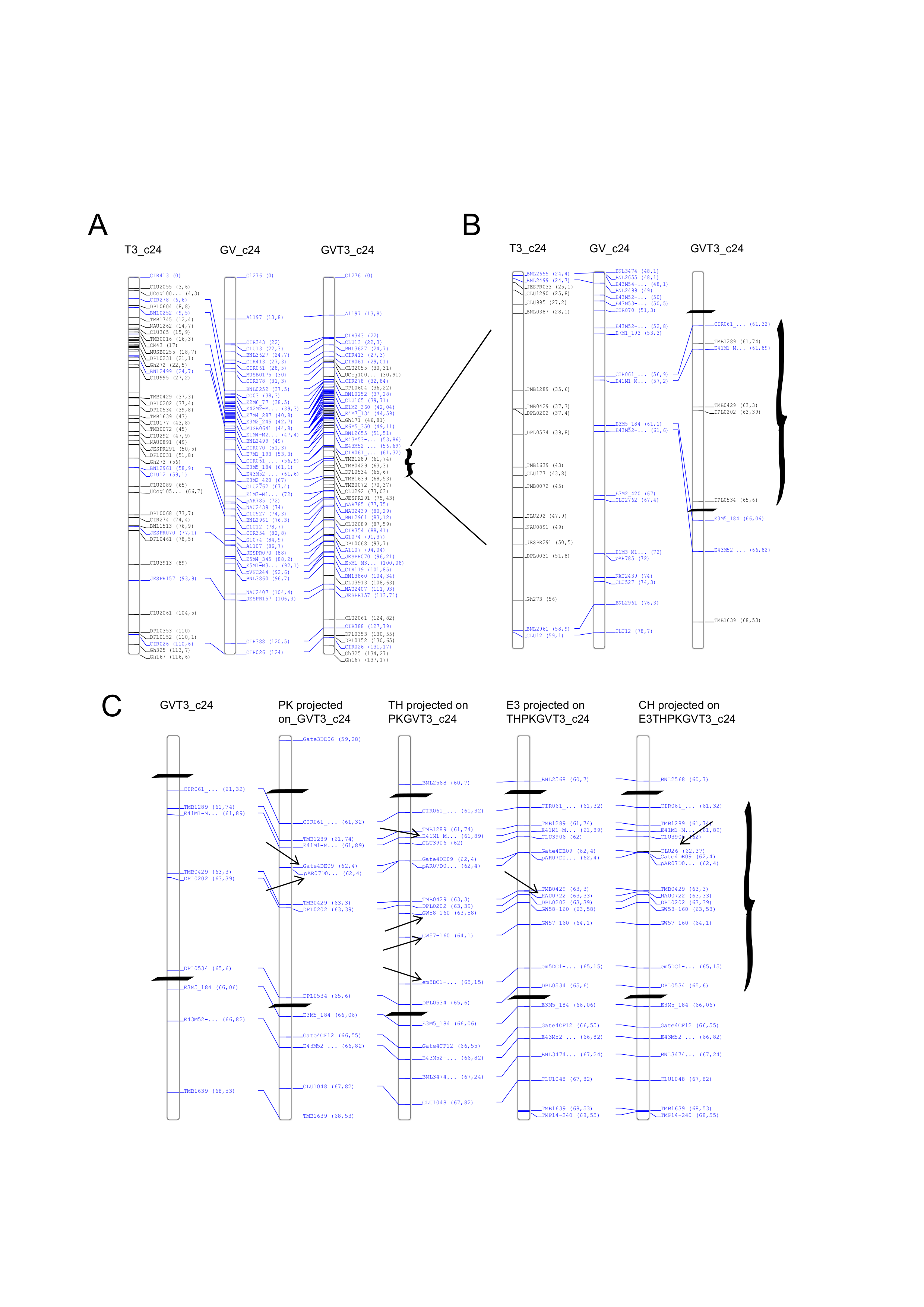

Supplement: Figure S2 — Construction of HDC map. Chromosome 24 shown as an example. Two-stage process for map integration of c24 and progressive enrichment of markers: 1st stage in upper panel showing T3, GV and GVT3 connections and a zoom over interval 61.3–65.6 cM, or 4.3 cM, between CIR061-DPL0534 (4 loci, 4.3 cM); and 2nd stage in lower panel with same interval enriched iteratively with 2 loci from PK, 4 loci from TH, 1 locus from E3 and 1 locus from CH, for a final density of 14 loci in the same interval distance. (TIF) [file pone.0045739.s002.tif]
