# Supplementary material for: A High Density Consensus Genetic Map of Tetraploid Cotton That Integrates Multiple Component Maps through Molecular Marker Redundancy Check
Source: PLoS One. 2012 Sep 24;7(9):e45739. doi: 10.1371/journal.pone.0045739 (PMC3454346; doi:10.1371/journal.pone.0045739)
Supplement: Figure S3 — The HDC genetic map of tetraploid cotton. Bridge markers (mapped in more than one component map) are highlighted in red (actual marker name) or in green (cluster of markers). Chromosomes of the A subgenome, c1 through c13 are presented in Figure S3A and of the D subgenome, c14 through c26, are presented in Figure S3B. (PDF) [file pone.0045739.s003.pdf]

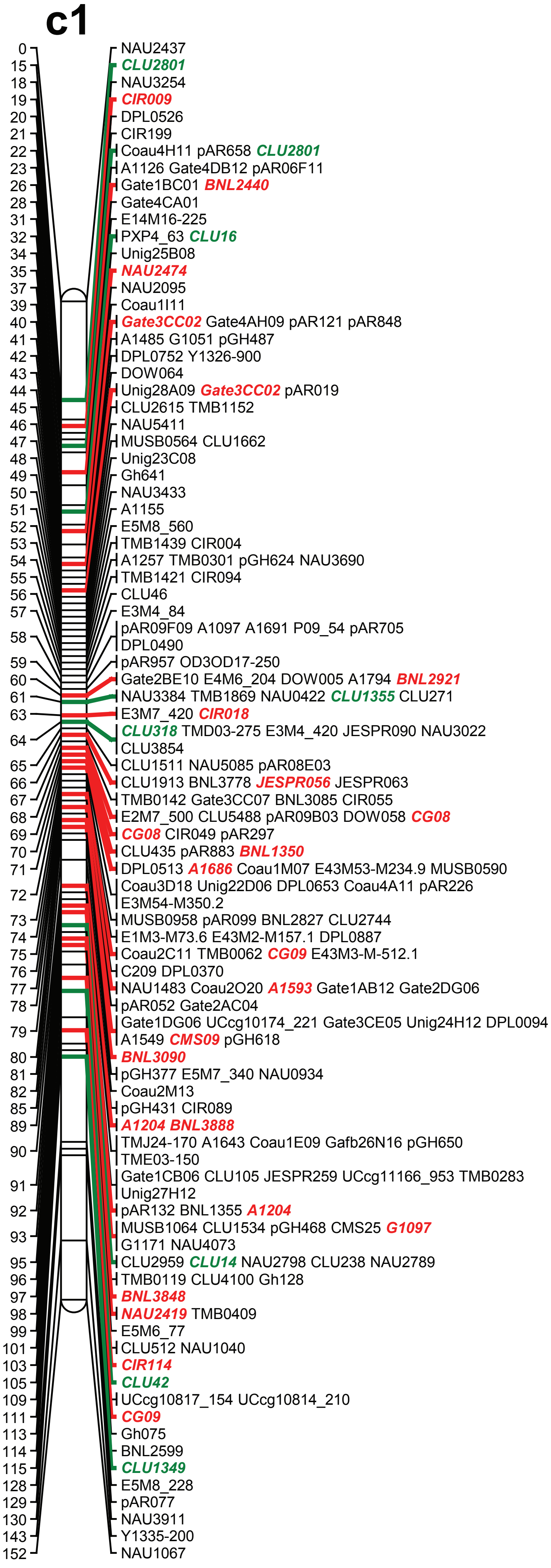

c1
0 NAU2437
15 CLU2801
18 NAU3254
19 CIR009
20 DPL0526
21 CIR199
22 Coau4H11 pAR658 CLU2801
23 A1126 Gate4DB12 pAR06F11
26 Gate1BC01 BNL2440
28 Gate4CA01
31 E14M16-225
32 PXP4_63 CLU16
34 Unig25B08
35 NAU2474
37 NAU2095
39 Coau1I11
40 Gate3CC02 Gate4AH09 pAR121 pAR848
41 A1485 G1051 pGH487
42 DPL0752 Y1326-900
43 DOW064
44 Unig28A09 Gate3CC02 pAR019
45 CLU2615 TMB1152
46 NAU5411
47 MUSB0564 CLU1662
48 Unig23C08
49 Gh641
50 NAU3433
51 A1155
52 E5M8_560
53 TMB1439 CIR004
54 A1257 TMB0301 pGH624 NAU3690
55 TMB1421 CIR094
56 CLU46
57 E3M4_84
58 pAR09F09 A1097 A1691 P09_54 pAR705
DPL0490
59 pAR957 OD3OD17-250
60 Gate2BE10 E4M6_204 DOW005 A1794 BNL2921
61 NAU3384 TMB1869 NAU0422 CLU1355 CLU271
63 E3M7_420 CIR018
64 CLU318 TMD03-275 E3M4_420 JESPR090 NAU3022
CLU3854
65 CLU1511 NAU5085 pAR08E03
66 CLU1913 BNL3778 JESPR056 JESPR063
67 TMB0142 Gate3CC07 BNL3085 CIR055
68 E2M7_500 CLU5488 pAR09B03 DOW058 CG08
69 CG08 CIR049 pAR297
70 CLU435 pAR883 BNL1350
71 DPL0513 A1686 Coau1M07 E43M53-M234.9 MUSB0590
72 Coau3D18 Unig22D06 DPL0653 Coau4A11 pAR226
E3M54-M350.2
73 MUSB0958 pAR099 BNL2827 CLU2744
74 E1M3-M73.6 E43M2-M157.1 DPL0887
75 Coau2C11 TMB0062 CG09 E43M3-M-512.1
76 C209 DPL0370
77 NAU1483 Coau2O20 A1593 Gate1AB12 Gate2DG06
78 pAR052 Gate2AC04
79 Gate1DG06 UCcg10174_221 Gate3CE05 Unig24H12 DPL0094
A1549 CMS09 pGH618
80 BNL3090
81 pGH377 E5M7_340 NAU0934
82 Coau2M13
85 pGH431 CIR089
89 A1204 BNL3888
90 TMJ24-170 A1643 Coau1E09 Gafb26N16 pGH650
TME03-150
91 Gate1CB06 CLU105 JESPR259 UCcg11166_953 TMB0283
Unig27H12
92 pAR132 BNL1355 A1204
93 MUSB1064 CLU1534 pGH468 CMS25 G1097
G1171 NAU4073
95 CLU2959 CLU14 NAU2798 CLU238 NAU2789
96 TMB0119 CLU4100 Gh128
97 BNL3848
98 NAU2419 TMB0409
99 E5M6_77
101 CLU512 NAU1040
103 CIR114
105 CLU42
109 UCcg10817_154 UCcg10814_210
111 CG09
113 Gh075
114 BNL2599
115 CLU1349
128 E5M8_228
129 pAR077
130 NAU3911
143 Y1335-200
152 NAU1067

c2

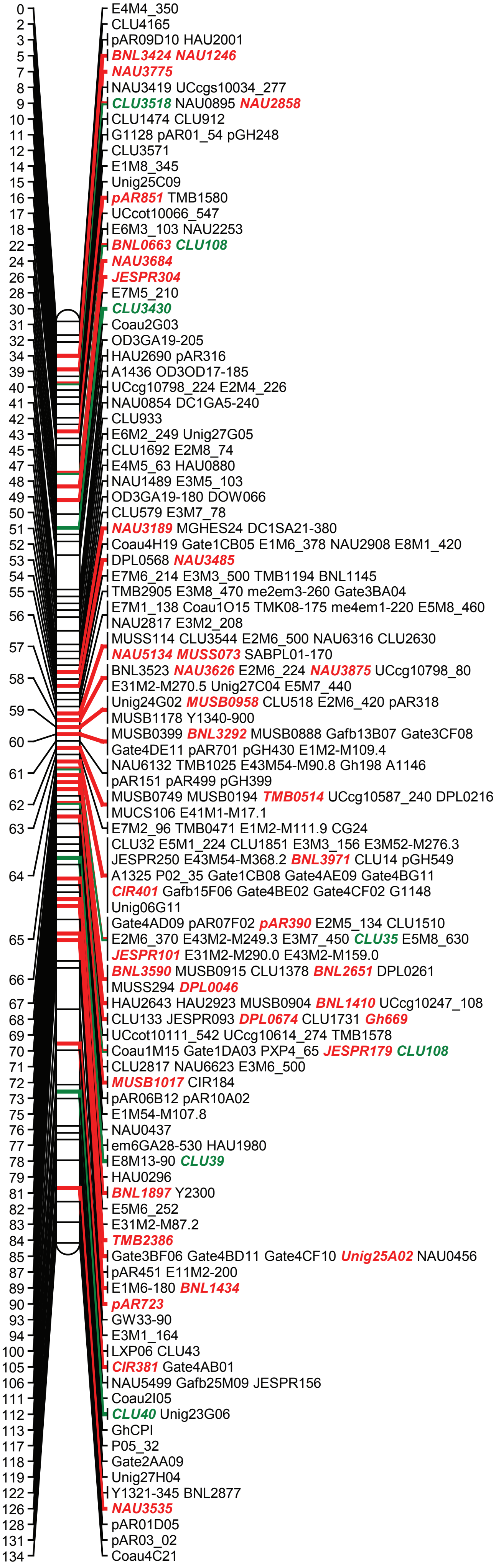
0
2
3
5
7
8
9
10
11
12
14
15
16
17
18
22
24
26
28
30
31
32
34
39
40
41
42
43
45
47
48
49
50
51
52
53
54
55
56
57
58
59
60
61
62
63
64
65
66
67
68
69
70
71
72
73
75
76
77
78
79
81
82
83
84
85
87
89
90
93
94
100
105
106
111
112
113
117
118
119
122
126
128
131
134
E4M4_350
CLU4165
pAR09D10 HAU2001
BNL3424 NAU1246
NAU3775
NAU3419 UCcgs10034_277
CLU3518 NAU0895 NAU2858
CLU1474 CLU912
G1128 pAR01_54 pGH248
CLU3571
E1M8_345
Unig25C09
pAR851 TMB1580
UCcot10066_547
E6M3_103 NAU2253
BNL0663 CLU108
NAU3684
JESPR304
E7M5_210
CLU3430
Coau2G03
OD3GA19-205
HAU2690 pAR316
A1436 OD3OD17-185
UCcg10798_224 E2M4_226
NAU0854 DC1GA5-240
CLU933
E6M2_249 Unig27G05
CLU1692 E2M8_74
E4M5_63 HAU0880
NAU1489 E3M5_103
OD3GA19-180 DOW066
CLU579 E3M7_78
NAU3189 MGHES24 DC1SA21-380
Coau4H19 Gate1CB05 E1M6_378 NAU2908 E8M1_420
DPL0568 NAU3485
E7M6_214 E3M3_500 TMB1194 BNL1145
TMB2905 E3M8_470 me2em3-260 Gate3BA04
E7M1_138 Coau1O15 TMK08-175 me4em1-220 E5M8_460
NAU2817 E3M2_208
MUSS114 CLU3544 E2M6_500 NAU6316 CLU2630
NAU5134 MUSS073 SABPL01-170
BNL3523 NAU3626 E2M6_224 NAU3875 UCcg10798_80
E31M2-M270.5 Unig27C04 E5M7_440
Unig24G02 MUSB0958 CLU518 E2M6_420 pAR318
MUSB1178 Y1340-900
MUSB0399 BNL3292 MUSB0888 Gafb13B07 Gate3CF08
Gate4DE11 pAR701 pGH430 E1M2-M109.4
NAU6132 TMB1025 E43M54-M90.8 Gh198 A1146
pAR151 pAR499 pGH399
MUSB0749 MUSB0194 TMB0514 UCcg10587_240 DPL0216
MUCS106 E41M1-M17.1
E7M2_96 TMB0471 E1M2-M111.9 CG24
CLU32 E5M1_224 CLU1851 E3M3_156 E3M52-M276.3
JESPR250 E43M54-M368.2 BNL3971 CLU14 pGH549
A1325 P02_35 Gate1CB08 Gate4AE09 Gate4BG11
CIR401 Gafb15F06 Gate4BE02 Gate4CF02 G1148
Unig06G11
Gate4AD09 pAR07F02 pAR390 E2M5_134 CLU1510
E2M6_370 E43M2-M249.3 E3M7_450 CLU35 E5M8_630
JESPR101 E31M2-M290.0 E43M2-M159.0
BNL3590 MUSB0915 CLU1378 BNL2651 DPL0261
MUSS294 DPL0046
HAU2643 HAU2923 MUSB0904 BNL1410 UCcg10247_108
CLU133 JESPR093 DPL0674 CLU1731 Gh669
UCcot10111_542 UCcg10614_274 TMB1578
Coau1M15 Gate1DA03 PXP4_65 JESPR179 CLU108
CLU2817 NAU6623 E3M6_500
MUSB1017 CIR184
pAR06B12 pAR10A02
E1M54-M107.8
NAU0437
em6GA28-530 HAU1980
E8M13-90 CLU39
HAU0296
BNL1897 Y2300
E5M6_252
E31M2-M87.2
TMB2386
Gate3BF06 Gate4BD11 Gate4CF10 Unig25A02 NAU0456
pAR451 E11M2-200
E1M6-180 BNL1434
pAR723
GW33-90
E3M1_164
LXP06 CLU43
CIR381 Gate4AB01
NAU5499 Gafb25M09 JESPR156
Coau2I05
CLU40 Unig23G06
GhCPI
P05_32
Gate2AA09
Unig27H04
Y1321-345 BNL2877
NAU3535
pAR01D05
pAR03_02
Coau4C21

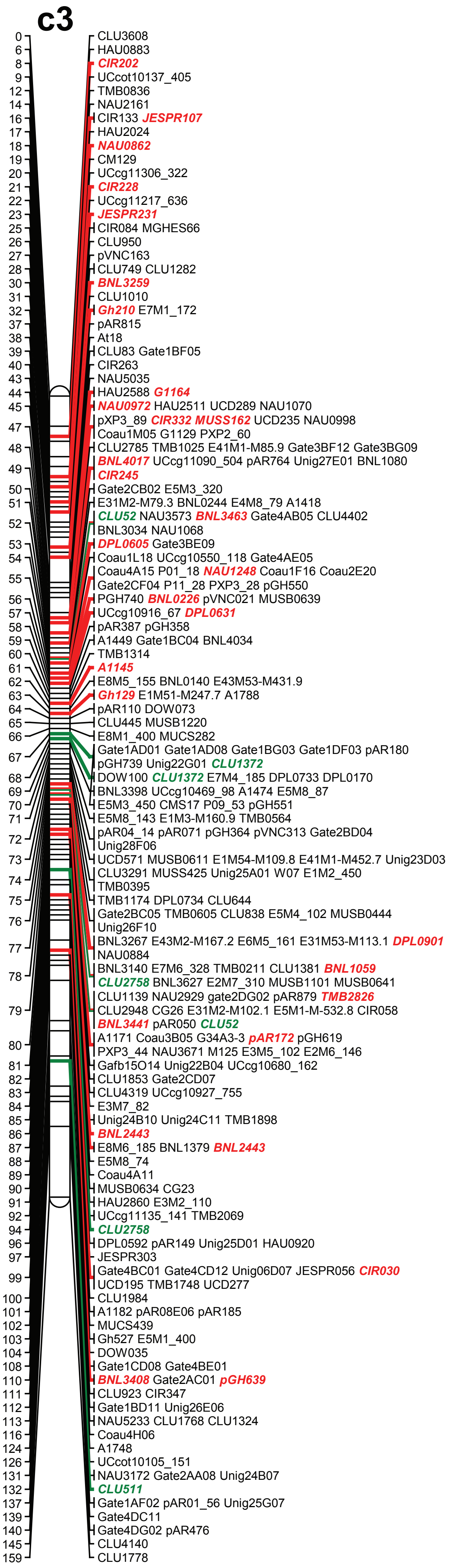
c3
0
6
8
9
12
14
16
17
18
19
20
21
22
23
25
26
27
28
30
31
32
37
38
39
40
43
44
45
47
48
49
50
51
52
53
54
55
56
57
58
59
60
61
62
63
64
65
66
67
68
69
70
71
72
73
74
75
76
77
78
79
80
81
82
83
84
85
86
87
88
89
90
91
92
94
96
97
99
100
101
102
103
104
108
110
111
112
113
116
124
126
131
132
137
139
140
145
159
CLU3608
HAU0883
CIR202
UCcot10137_405
TMB0836
NAU2161
CIR133 JESPR107
HAU2024
NAU0862
CM129
UCcg11306_322
CIR228
UCcg11217_636
JESPR231
CIR084 MGHES66
CLU950
pVNC163
CLU749 CLU1282
BNL3259
CLU1010
Gh210 E7M1_172
pAR815
At18
CLU83 Gate1BF05
CIR263
NAU5035
HAU2588 G1164
NAU0972 HAU2511 UCD289 NAU1070
pXP3_89 CIR332 MUSS162 UCD235 NAU0998
Coau1M05 G1129 PXP2_60
CLU2785 TMB1025 E41M1-M85.9 Gate3BF12 Gate3BG09
BNL4017 UCcg11090_504 pAR764 Unig27E01 BNL1080
CIR245
Gate2CB02 E5M3_320
E31M2-M79.3 BNL0244 E4M8_79 A1418
CLU52 NAU3573 BNL3463 Gate4AB05 CLU4402
BNL3034 NAU1068
DPL0605 Gate3BE09
Coau1L18 UCcg10550_118 Gate4AE05
Coau4A15 P01_18 NAU1248 Coau1F16 Coau2E20
Gate2CF04 P11_28 PXP3_28 pGH550
PGH740 BNL0226 pVNC021 MUSB0639
UCcg10916_67 DPL0631
pAR387 pGH358
A1449 Gate1BC04 BNL4034
TMB1314
A1145
E8M5_155 BNL0140 E43M53-M431.9
Gh129 E1M51-M247.7 A1788
pAR110 DOW073
CLU445 MUSB1220
E8M1_400 MUCS282
Gate1AD01 Gate1AD08 Gate1BG03 Gate1DF03 pAR180
pGH739 Unig22G01 CLU1372
DOW100 CLU1372 E7M4_185 DPL0733 DPL0170
BNL3398 UCcg10469_98 A1474 E5M8_87
E5M3_450 CMS17 P09_53 pGH551
E5M8_143 E1M3-M160.9 TMB0564
pAR04_14 pAR071 pGH364 pVNC313 Gate2BD04
Unig28F06
UCD571 MUSB0611 E1M54-M109.8 E41M1-M452.7 Unig23D03
CLU3291 MUSS425 Unig25A01 W07 E1M2_450
TMB0395
TMB1174 DPL0734 CLU644
Gate2BC05 TMB0605 CLU838 E5M4_102 MUSB0444
Unig26F10
BNL3267 E43M2-M167.2 E6M5_161 E31M53-M113.1 DPL0901
NAU0884
BNL3140 E7M6_328 TMB0211 CLU1381 BNL1059
CLU2758 BNL3627 E2M7_310 MUSB1101 MUSB0641
CLU1139 NAU2929 gate2DG02 pAR879 TMB2826
CLU2948 CG26 E31M2-M102.1 E5M1-M-532.8 CIR058
BNL3441 pAR050 CLU52
A1171 Coau3B05 G34A3-3 pAR172 pGH619
PXP3_44 NAU3671 M125 E3M5_102 E2M6_146
Gafb15O14 Unig22B04 UCcg10680_162
CLU1853 Gate2CD07
CLU4319 UCcg10927_755
E3M7_82
Unig24B10 Unig24C11 TMB1898
BNL2443
E8M6_185 BNL1379 BNL2443
E5M8_74
Coau4A11
MUSB0634 CG23
HAU2860 E3M2_110
UCcg11135_141 TMB2069
CLU2758
DPL0592 pAR149 Unig25D01 HAU0920
JESPR303
Gate4BC01 Gate4CD12 Unig06D07 JESPR056 CIR030
UCD195 TMB1748 UCD277
CLU1984
A1182 pAR08E06 pAR185
MUCS439
Gh527 E5M1_400
DOW035
Gate1CD08 Gate4BE01
BNL3408 Gate2AC01 pGH639
CLU923 CIR347
Gate1BD11 Unig26E06
NAU5233 CLU1768 CLU1324
Coau4H06
A1748
UCcot10105_151
NAU3172 Gate2AA08 Unig24B07
CLU511
Gate1AF02 pAR01_56 Unig25G07
Gate4DC11
Gate4DG02 pAR476
CLU4140
CLU1778

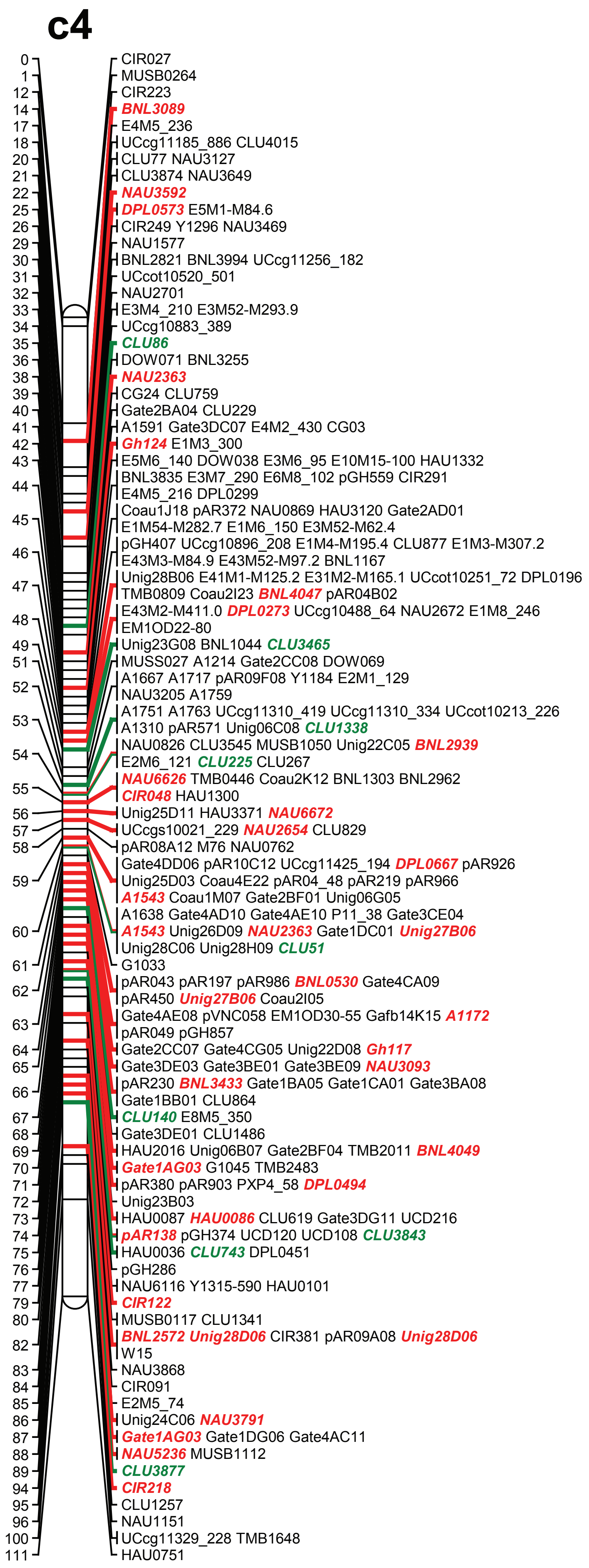
c4
0
1
12
14
17
18
20
21
22
25
26
29
30
31
32
33
34
35
36
38
39
40
41
42
43
44
45
46
47
48
49
51
52
53
54
55
56
57
58
59
60
61
62
63
64
65
66
67
68
69
70
71
72
73
74
75
76
77
79
80
82
83
84
85
86
87
88
89
94
95
96
100
111
CIR027
MUSB0264
CIR223
BNL3089
E4M5_236
UCcg11185_886 CLU4015
CLU77 NAU3127
CLU3874 NAU3649
NAU3592
DPL0573 E5M1-M84.6
CIR249 Y1296 NAU3469
NAU1577
BNL2821 BNL3994 UCcg11256_182
UCcot10520_501
NAU2701
E3M4_210 E3M52-M293.9
UCcg10883_389
CLU86
DOW071 BNL3255
NAU2363
CG24 CLU759
Gate2BA04 CLU229
A1591 Gate3DC07 E4M2_430 CG03
Gh124 E1M3_300
E5M6_140 DOW038 E3M6_95 E10M15-100 HAU1332
BNL3835 E3M7_290 E6M8_102 pGH559 CIR291
E4M5_216 DPL0299
Coau1J18 pAR372 NAU0869 HAU3120 Gate2AD01
E1M54-M282.7 E1M6_150 E3M52-M62.4
pGH407 UCcg10896_208 E1M4-M195.4 CLU877 E1M3-M307.2
E43M3-M84.9 E43M52-M97.2 BNL1167
Unig28B06 E41M1-M125.2 E31M2-M165.1 UCcot10251_72 DPL0196
TMB0809 Coau2I23 BNL4047 pAR04B02
E43M2-M411.0 DPL0273 UCcg10488_64 NAU2672 E1M8_246
EM1OD22-80
Unig23G08 BNL1044 CLU3465
MUSS027 A1214 Gate2CC08 DOW069
A1667 A1717 pAR09F08 Y1184 E2M1_129
NAU3205 A1759
A1751 A1763 UCcg11310_419 UCcg11310_334 UCcot10213_226
A1310 pAR571 Unig06C08 CLU1338
NAU0826 CLU3545 MUSB1050 Unig22C05 BNL2939
E2M6_121 CLU225 CLU267
NAU6626 TMB0446 Coau2K12 BNL1303 BNL2962
CIR048 HAU1300
Unig25D11 HAU3371 NAU6672
UCcgs10021_229 NAU2654 CLU829
pAR08A12 M76 NAU0762
Gate4DD06 pAR10C12 UCcg11425_194 DPL0667 pAR926
Unig25D03 Coau4E22 pAR04_48 pAR219 pAR966
A1543 Coau1M07 Gate2BF01 Unig06G05
A1638 Gate4AD10 Gate4AE10 P11_38 Gate3CE04
A1543 Unig26D09 NAU2363 Gate1DC01 Unig27B06
Unig28C06 Unig28H09 CLU51
G1033
pAR043 pAR197 pAR986 BNL0530 Gate4CA09
pAR450 Unig27B06 Coau2I05
Gate4AE08 pVNC058 EM1OD30-55 Gafb14K15 A1172
pAR049 pGH857
Gate2CC07 Gate4CG05 Unig22D08 Gh117
Gate3DE03 Gate3BE01 Gate3BE09 NAU3093
pAR230 BNL3433 Gate1BA05 Gate1CA01 Gate3BA08
Gate1BB01 CLU864
CLU140 E8M5_350
Gate3DE01 CLU1486
HAU2016 Unig06B07 Gate2BF04 TMB2011 BNL4049
Gate1AG03 G1045 TMB2483
pAR380 pAR903 PXP4_58 DPL0494
Unig23B03
HAU0087 HAU0086 CLU619 Gate3DG11 UCD216
pAR138 pGH374 UCD120 UCD108 CLU3843
HAU0036 CLU743 DPL0451
pGH286
NAU6116 Y1315-590 HAU0101
CIR122
MUSB0117 CLU1341
BNL2572 Unig28D06 CIR381 pAR09A08 Unig28D06
W15
NAU3868
CIR091
E2M5_74
Unig24C06 NAU3791
Gate1AG03 Gate1DG06 Gate4AC11
NAU5236 MUSB1112
CLU3877
CIR218
CLU1257
NAU1151
UCcg11329_228 TMB1648
HAU0751

c5

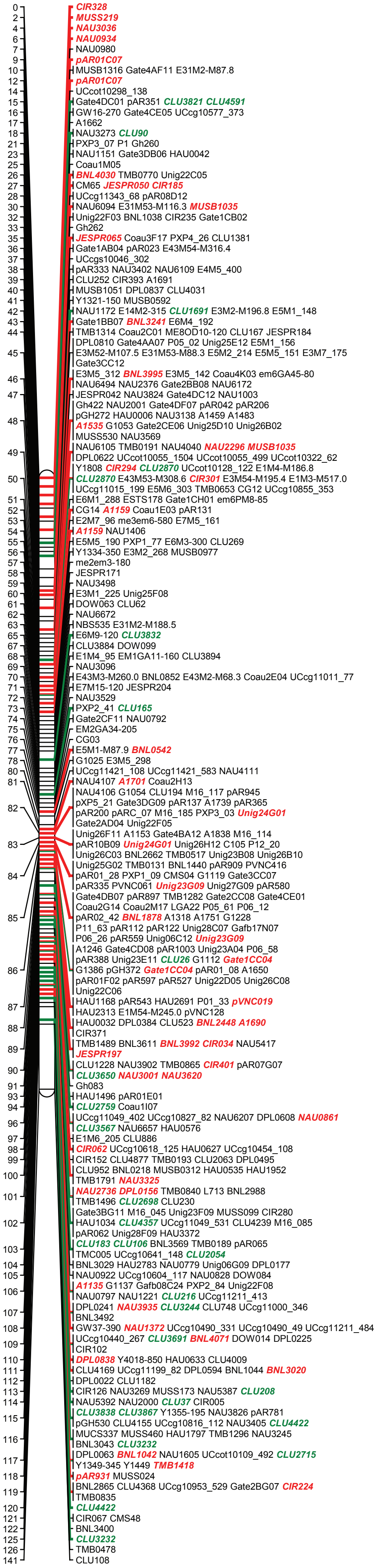
0 2 4 6 7 9 10 12 14 15 16 17 18 21 23 25 26 27 28 30 32 33 35 36 37 38 39 40 41 42 43 44 45 46 47 48 49 50 51 52 53 54 55 56 57 58 59 60 61 62 63 65 67 68 69 70 71 72 73 74 75 76 77 78 80 81 82 83 84 85 86 87 88 89 90 91 93 94 96 97 98 99 100 101 102 103 104 105 106 107 108 109 110 111 112 113 114 115 116 117 118 119 120 121 122 125 126 141
CIR328
MUSS219
NAU3036
NAU0934
NAU0980
pAR01C07
MUSB1316 Gate4AF11 E31M2-M87.8
pAR01C07
UCcot10298_138
Gate4DC01 pAR351 CLU3821 CLU4591
GW16-270 Gate4CE05 UCcg10577_373
A1662
NAU3273 CLU90
PXP3_07 P1 Gh260
NAU1151 Gate3DB06 HAU0042
Coau1M05
BNL4030 TMB0770 Unig22C05
CM65 JESPR050 CIR185
UCcg11343_68 pAR08D12
NAU6094 E31M53-M116.3 MUSB1035
Unig22F03 BNL1038 CIR235 Gate1CB02
Gh262
JESPR065 Coau3F17 PXP4_26 CLU1381
Gate1AB04 pAR023 E43M54-M316.4
UCcgs10046_302
pAR333 NAU3402 NAU6109 E4M5_400
CLU252 CIR393 A1691
MUSB1051 DPL0837 CLU4031
Y1321-150 MUSB0592
NAU1172 E14M2-315 CLU1691 E3M2-M196.8 E5M1_148
Gate1BB07 BNL3241 E6M4_192
TMB1314 Coau2C01 ME8OD10-120 CLU167 JESPR184
DPL0810 Gate4AA07 P05_02 Unig25E12 E5M1_156
E3M52-M107.5 E31M53-M88.3 E5M2_214 E5M5_151 E3M7_175
Gate3CC12
E3M5_312 BNL3995 E3M5_142 Coau4K03 em6GA45-80
NAU6494 NAU2376 Gate2BB08 NAU6172
JESPR042 NAU3824 Gate4DC12 NAU1003
Gh422 NAU2001 Gate4DF07 pAR042 pAR206
pGH272 HAU0006 NAU3138 A1459 A1483
A1535 G1053 Gate2CE06 Unig25D10 Unig26B02
MUSS530 NAU3569
NAU6105 TMB0191 NAU4040 NAU2296 MUSB1035
DPL0622 UCcot10055_1504 UCcot10055_499 UCcot10322_62
Y1808 CIR294 CLU2870 UCcot10128_122 E1M4-M186.8
CLU2870 E43M53-M308.6 CIR301 E3M54-M195.4 E1M3-M517.0
UCcg11015_199 E5M6_303 TMB0653 CG12 UCcg10855_353
E6M1_288 ESTS178 Gate1CH01 em6PM8-85
CG14 A1159 Coau1E03 pAR131
E2M7_96 me3em6-580 E7M5_161
A1159 NAU1406
E5M5_190 PXP1_77 E6M3-300 CLU269
Y1334-350 E3M2_268 MUSB0977
me2em3-180
JESPR171
NAU3498
E3M1_225 Unig25F08
DOW063 CLU62
NAU6672
NBS535 E31M2-M188.5
E6M9-120 CLU3832
CLU3884 DOW099
E1M4_95 EM1GA11-160 CLU3894
NAU3096
E43M3-M260.0 BNL0852 E43M2-M68.3 Coau2E04 UCcg11011_77
E7M15-120 JESPR204
NAU3529
PXP2_41 CLU165
Gate2CF11 NAU0792
EM2GA34-205
CG03
E5M1-M87.9 BNL0542
G1025 E3M5_298
UCcg11421_108 UCcg11421_583 NAU4111
NAU4107 A1701 Coau2H13
NAU4106 G1054 CLU194 M16_117 pAR945
pXP5_21 Gate3DG09 pAR137 A1739 pAR365
pAR200 pARC_07 M16_185 PXP3_03 Unig24G01
Gate2AD04 Unig22F05
Unig26F11 A1153 Gate4BA12 A1838 M16_114
pAR10B09 Unig24G01 Unig26H12 C105 P12_20
Unig26C03 BNL2662 TMB0517 Unig23B08 Unig26B10
Unig25G02 TMB0131 BNL1440 pAR909 PVNC416
pAR01_28 PXP1_09 CMS04 G1119 Gate3CC07
pAR335 PVNC061 Unig23G09 Unig27G09 pAR580
Gate4DB07 pAR897 TMB1282 Gate2CC08 Gate4CE01
Coau2G14 Coau2M17 LGA22 P05_61 P06_12
pAR02_42 BNL1878 A1318 A1751 G1228
P11_63 pAR112 pAR122 Unig28C07 Gafb17N07
P06_26 pAR559 Unig06C12 Unig23G09
A1246 Gate4CD08 pAR1003 Unig23A04 P06_58
pAR388 Unig23E11 CLU26 G1112 Gate1CC04
G1386 pGH372 Gate1CC04 pAR01_08 A1650
pAR01F02 pAR597 pAR527 Unig22D05 Unig26C08
Unig22C06
HAU1168 pAR543 HAU2691 P01_33 pVNC019
HAU2313 E1M54-M245.0 pVNC128
HAU0032 DPL0384 CLU523 BNL2448 A1690
CIR371
TMB1489 BNL3611 BNL3992 CIR034 NAU5417
JESPR197
CLU1228 NAU3902 TMB0865 CIR401 pAR07G07
CLU3650 NAU3001 NAU3620
Gh083
HAU1496 pAR01E01
CLU2759 Coau1I07
UCcg11049_402 UCcg10827_82 NAU6207 DPL0608 NAU0861
CLU3567 NAU6657 HAU0576
E1M6_205 CLU886
CIR062 UCcg10618_125 HAU0627 UCcg10454_108
CIR152 CLU4877 TMB0193 CLU2063 DPL0495
CLU952 BNL0218 MUSB0312 HAU0535 HAU1952
TMB1791 NAU3325
NAU2736 DPL0156 TMB0840 L713 BNL2988
TMB1496 CLU2698 CLU230
Gate3BG11 M16_045 Unig23F09 MUSS099 CIR280
HAU1034 CLU4357 UCcg11049_531 CLU4239 M16_085
pAR062 Unig28F09 HAU3372
CLU183 CLU106 BNL3569 TMB0189 pAR065
TMC005 UCcg10641_148 CLU2054
BNL3029 HAU2783 NAU0779 Unig06G09 DPL0177
NAU0922 UCcg10604_117 NAU0828 DOW084
A1135 G1137 Gafb08C24 PXP2_84 Unig22F08
NAU0797 NAU1221 CLU216 UCcg11211_413
DPL0241 NAU3935 CLU3244 CLU748 UCcg11000_346
BNL3492
GW37-390 NAU1372 UCcg10490_331 UCcg10490_49 UCcg11211_484
UCcg10440_267 CLU3691 BNL4071 DOW014 DPL0225
CIR102
DPL0838 Y4018-850 HAU0633 CLU4009
CLU4169 UCcg11199_82 DPL0594 BNL1044 BNL3020
DPL0022 CLU1182
CIR126 NAU3269 MUSS173 NAU5387 CLU208
NAU5392 NAU2000 CLU37 CIR005
CLU3838 CLU3867 Y1355-195 NAU3826 pAR781
pGH530 CLU4155 UCcg10816_112 NAU3405 CLU4422
MUCS337 MUSS460 HAU1797 TMB1296 NAU3245
BNL3043 CLU3232
DPL0063 BNL1042 NAU1605 UCcot10109_492 CLU2715
Y1349-345 Y1449 TMB1418
pAR931 MUSS024
BNL2865 CLU4368 UCcg10953_529 Gate2BG07 CIR224
TMB0835
CLU4422
CIR067 CMS48
BNL3400
CLU3232
TMB0478
CLU108

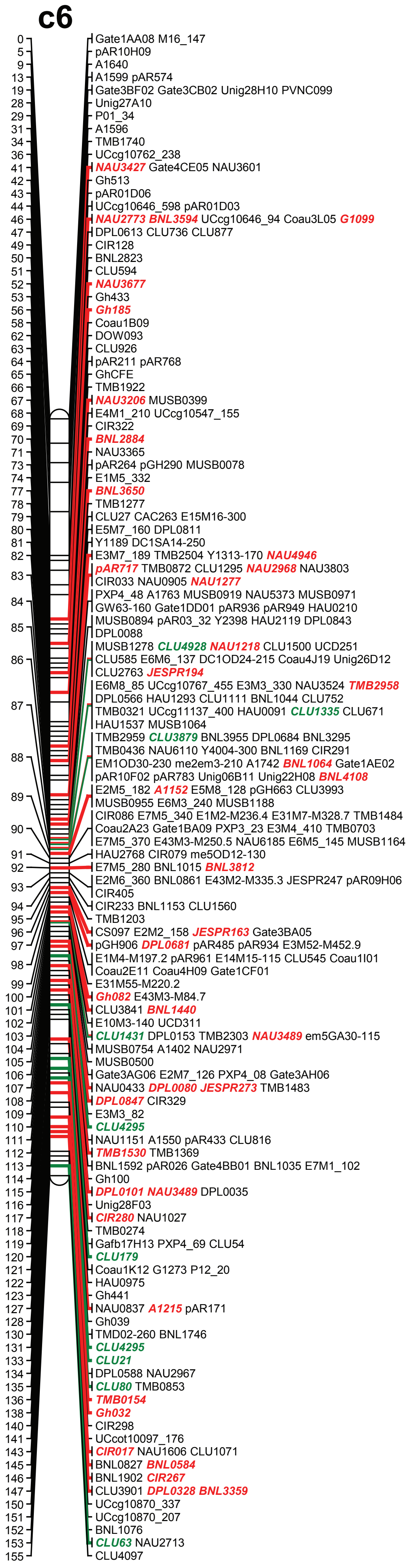
c6
Gate1AA08 M16_147
pAR10H09
A1640
A1599 pAR574
Gate3BF02 Gate3CB02 Unig28H10 PVNC099
Unig27A10
P01_34
A1596
TMB1740
UCcg10762_238
NAU3427 Gate4CE05 NAU3601
Gh513
pAR01D06
UCcg10646_598 pAR01D03
NAU2773 BNL3594 UCcg10646_94 Coau3L05 G1099
DPL0613 CLU736 CLU877
CIR128
BNL2823
CLU594
NAU3677
Gh433
Gh185
Coau1B09
DOW093
CLU926
pAR211 pAR768
GhCFE
TMB1922
NAU3206 MUSB0399
E4M1_210 UCcg10547_155
CIR322
BNL2884
NAU3365
pAR264 pGH290 MUSB0078
E1M5_332
BNL3650
TMB1277
CLU27 CAC263 E15M16-300
E5M7_160 DPL0811
Y1189 DC1SA14-250
E3M7_189 TMB2504 Y1313-170 NAU4946
pAR717 TMB0872 CLU1295 NAU2968 NAU3803
CIR033 NAU0905 NAU1277
PXP4_48 A1763 MUSB0919 NAU5373 MUSB0971
GW63-160 Gate1DD01 pAR936 pAR949 HAU0210
MUSB0894 pAR03_32 Y2398 HAU2119 DPL0843
DPL0088
MUSB1278 CLU4928 NAU1218 CLU1500 UCD251
CLU585 E6M6_137 DC1OD24-215 Coau4J19 Unig26D12
CLU2763 JESPR194
E6M8_85 UCcg10767_455 E3M3_330 NAU3524 TMB2958
DPL0566 HAU1293 CLU1111 BNL1044 CLU752
TMB0321 UCcg11137_400 HAU0091 CLU1335 CLU671
HAU1537 MUSB1064
TMB2959 CLU3879 BNL3955 DPL0684 BNL3295
TMB0436 NAU6110 Y4004-300 BNL1169 CIR291
EM1OD30-230 me2em3-210 A1742 BNL1064 Gate1AE02
pAR10F02 pAR783 Unig06B11 Unig22H08 BNL4108
E2M5_182 A1152 E5M8_128 pGH663 CLU3993
MUSB0955 E6M3_240 MUSB1188
CIR086 E7M5_340 E1M2-M236.4 E31M7-M328.7 TMB1484
Coau2A23 Gate1BA09 PXP3_23 E3M4_410 TMB0703
E7M5_370 E43M3-M250.5 NAU6185 E6M5_145 MUSB1164
HAU2768 CIR079 me5OD12-130
E7M5_280 BNL1015 BNL3812
E2M6_360 BNL0861 E43M2-M335.3 JESPR247 pAR09H06
CIR405
CIR233 BNL1153 CLU1560
TMB1203
CS097 E2M2_158 JESPR163 Gate3BA05
pGH906 DPL0681 pAR485 pAR934 E3M52-M452.9
E1M4-M197.2 pAR961 E14M15-115 CLU545 Coau1I01
Coau2E11 Coau4H09 Gate1CF01
E31M55-M220.2
Gh082 E43M3-M84.7
CLU3841 BNL1440
E10M3-140 UCD311
CLU1431 DPL0153 TMB2303 NAU3489 em5GA30-115
MUSB0754 A1402 NAU2971
MUSB0500
Gate3AG06 E2M7_126 PXP4_08 Gate3AH06
NAU0433 DPL0080 JESPR273 TMB1483
DPL0847 CIR329
E3M3_82
CLU4295
NAU1151 A1550 pAR433 CLU816
TMB1530 TMB1369
BNL1592 pAR026 Gate4BB01 BNL1035 E7M1_102
Gh100
DPL0101 NAU3489 DPL0035
Unig28F03
CIR280 NAU1027
TMB0274
Gafb17H13 PXP4_69 CLU54
CLU179
Coau1K12 G1273 P12_20
HAU0975
Gh441
NAU0837 A1215 pAR171
Gh039
TMD02-260 BNL1746
CLU4295
CLU21
DPL0588 NAU2967
CLU80 TMB0853
TMB0154
Gh032
CIR298
UCcot10097_176
CIR017 NAU1606 CLU1071
BNL0827 BNL0584
BNL1902 CIR267
CLU3901 DPL0328 BNL3359
UCcg10870_337
UCcg10870_207
BNL1076
CLU63 NAU2713
CLU4097

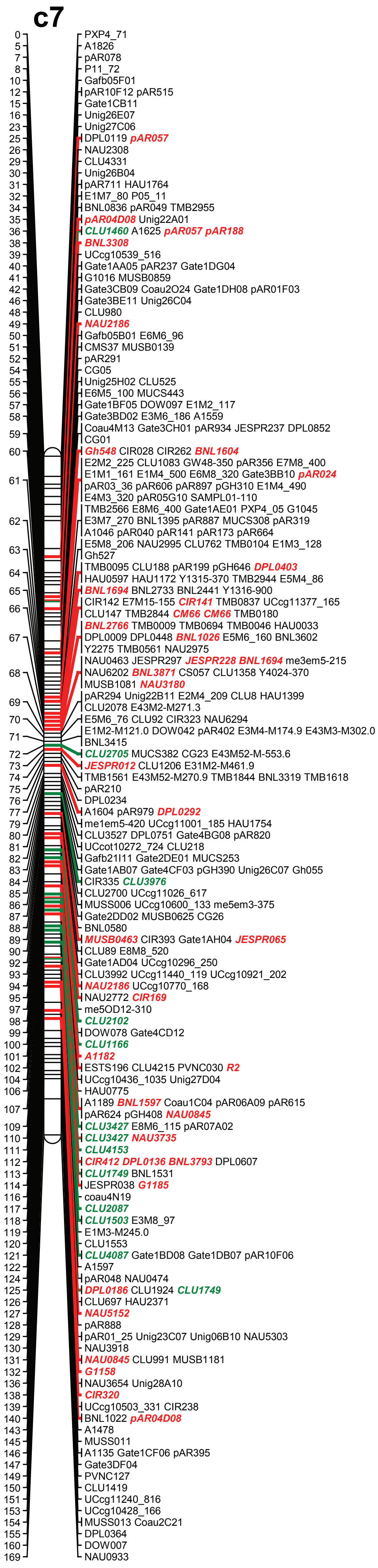
c7
0
5
7
8
10
12
15
16
23
25
26
29
30
31
32
34
35
36
38
39
40
41
42
46
48
49
50
51
52
54
55
56
57
58
59
60
61
62
63
64
65
66
67
68
69
70
71
72
73
74
75
76
77
79
80
81
82
83
84
85
86
87
88
89
90
92
93
94
95
97
98
99
100
101
102
104
106
107
109
110
111
112
113
114
116
117
118
119
120
121
122
124
125
126
127
128
129
130
131
132
136
138
139
140
143
145
146
147
149
150
151
153
154
155
160
169
PXP4_71
A1826
pAR078
P11_72
Gafb05F01
pAR10F12 pAR515
Gate1CB11
Unig26E07
Unig27C06
DPL0119 pAR057
NAU2308
CLU4331
Unig26B04
pAR711 HAU1764
E1M7_80 P05_11
BNL0836 pAR049 TMB2955
pAR04D08 Unig22A01
CLU1460 A1625 pAR057 pAR188
BNL3308
UCcg10539_516
Gate1AA05 pAR237 Gate1DG04
G1016 MUSB0859
Gate3CB09 Coau2O24 Gate1DH08 pAR01F03
Gate3BE11 Unig26C04
CLU980
NAU2186
Gafb05B01 E6M6_96
CMS37 MUSB0139
pAR291
CG05
Unig25H02 CLU525
E6M5_100 MUCS443
Gate1BF05 DOW097 E1M2_117
Gate3BD02 E3M6_186 A1559
Coau4M13 Gate3CH01 pAR934 JESPR237 DPL0852
CG01
Gh548 CIR028 CIR262 BNL1604
E2M2_225 CLU1083 GW48-350 pAR356 E7M8_400
E1M1_161 E1M4_500 E6M8_320 Gate3BB10 pAR024
pAR03_36 pAR606 pAR897 pGH310 E1M4_490
E4M3_320 pAR05G10 SAMPL01-110
TMB2566 E8M6_400 Gate1AE01 PXP4_05 G1045
E3M7_270 BNL1395 pAR887 MUCS308 pAR319
A1046 pAR040 pAR141 pAR173 pAR664
E5M8_206 NAU2995 CLU762 TMB0104 E1M3_128
Gh527
TMB0095 CLU188 pAR199 pGH646 DPL0403
HAU0597 HAU1172 Y1315-370 TMB2944 E5M4_86
BNL1694 BNL2733 BNL2441 Y1316-900
CIR142 E7M15-155 CIR141 TMB0837 UCcg11377_165
CLU147 TMB2844 CM66 CM66 TMB0180
BNL2766 TMB0009 TMB0694 TMB0046 HAU0033
DPL0009 DPL0448 BNL1026 E5M6_160 BNL3602
Y2275 TMB0561 NAU2975
NAU0463 JESPR297 JESPR228 BNL1694 me3em5-215
NAU6202 BNL3871 CS057 CLU1358 Y4024-370
MUSB1081 NAU3180
pAR294 Unig22B11 E2M4_209 CLU8 HAU1399
CLU2078 E43M2-M271.3
E5M6_76 CLU92 CIR323 NAU6294
E1M2-M121.0 DOW042 pAR402 E3M4-M174.9 E43M3-M302.0
BNL3415
CLU2705 MUCS382 CG23 E43M52-M-553.6
JESPR012 CLU1206 E31M2-M461.9
TMB1561 E43M52-M270.9 TMB1844 BNL3319 TMB1618
pAR210
DPL0234
A1604 pAR979 DPL0292
me1em5-420 UCcg11001_185 HAU1754
CLU3527 DPL0751 Gate4BG08 pAR820
UCcot10272_724 CLU218
Gafb21I11 Gate2DE01 MUCS253
Gate1AB07 Gate4CF03 pGH390 Unig26C07 Gh055
CIR335 CLU3976
CLU2700 UCcg11026_617
MUSS006 UCcg10600_133 me5em3-375
Gate2DD02 MUSB0625 CG26
BNL0580
MUSB0463 CIR393 Gate1AH04 JESPR065
CLU89 E8M8_520
Gate1AD04 UCcg10296_250
CLU3992 UCcg11440_119 UCcg10921_202
NAU2186 UCcg10770_168
NAU2772 CIR169
me5OD12-310
CLU2102
DOW078 Gate4CD12
CLU1166
A1182
ESTS196 CLU4215 PVNC030 R2
UCcg10436_1035 Unig27D04
HAU0775
A1189 BNL1597 Coau1C04 pAR06A09 pAR615
pAR624 pGH408 NAU0845
CLU3427 E8M6_115 pAR07A02
CLU3427 NAU3735
CLU4153
CIR412 DPL0136 BNL3793 DPL0607
CLU1749 BNL1531
JESPR038 G1185
coau4N19
CLU2087
CLU1503 E3M8_97
E1M3-M245.0
CLU1553
CLU4087 Gate1BD08 Gate1DB07 pAR10F06
A1597
pAR048 NAU0474
DPL0186 CLU1924 CLU1749
CLU697 HAU2371
NAU5152
pAR888
pAR01_25 Unig23C07 Unig06B10 NAU5303
NAU3918
NAU0845 CLU991 MUSB1181
G1158
NAU3654 Unig28A10
CIR320
UCcg10503_331 CIR238
BNL1022 pAR04D08
A1478
MUSS011
A1135 Gate1CF06 pAR395
Gate3DF04
PVNC127
CLU1419
UCcg11240_816
UCcg10428_166
MUSS013 Coau2C21
DPL0364
DOW007
NAU0933

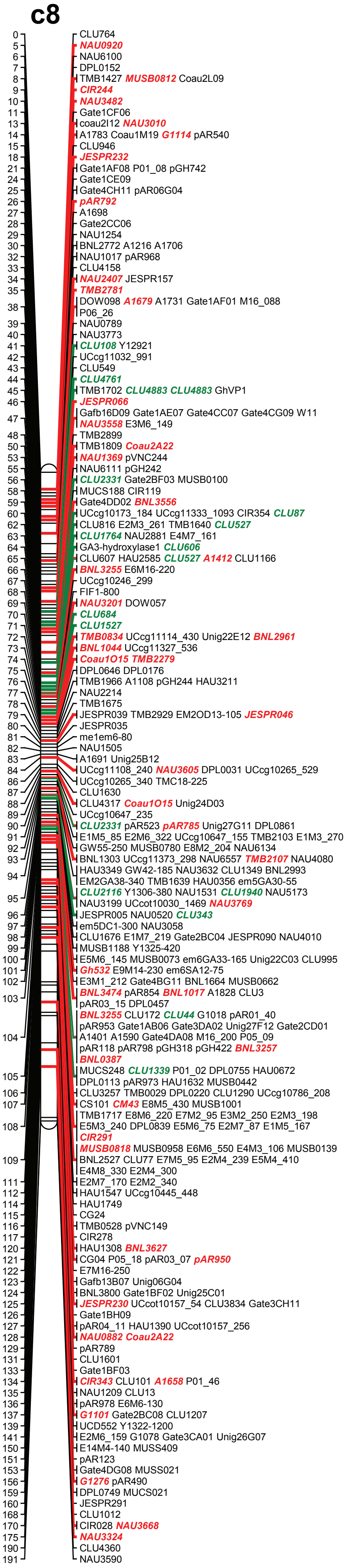
c8
0 5 6 7 8 9 10 11 13 14 15 18 21 24 25 26 27 28 29 30 32 33 34 35 38 39 40 41 42 43 44 45 46 47 48 50 53 55 56 58 59 60 62 63 64 65 66 67 68 69 70 71 72 73 74 75 76 77 78 79 80 81 82 83 84 86 87 88 89 90 91 92 93 94 95 96 97 98 99 100 101 102 103 104 105 106 107 108 109 111 112 114 115 116 117 120 121 122 123 124 125 126 127 128 129 131 133 134 135 136 137 139 141 150 151 153 156 159 160 168 170 175 190 191
CLU764
NAU0920
NAU6100
DPL0152
TMB1427 MUSB0812 Coau2L09
CIR244
NAU3482
Gate1CF06
coau2I12 NAU3010
A1783 Coau1M19 G1114 pAR540
CLU946
JESPR232
Gate1AF08 P01_08 pGH742
Gate1CE09
Gate4CH11 pAR06G04
pAR792
A1698
Gate2CC06
NAU1254
BNL2772 A1216 A1706
NAU1017 pAR968
CLU4158
NAU2407 JESPR157
TMB2781
DOW098 A1679 A1731 Gate1AF01 M16_088
P06_26
NAU0789
NAU3773
CLU108 Y12921
UCcg11032_991
CLU549
CLU4761
TMB1702 CLU4883 CLU4883 GhVP1
JESPR066
Gafb16D09 Gate1AE07 Gate4CC07 Gate4CG09 W11
NAU3558 E3M6_149
TMB2899
TMB1809 Coau2A22
NAU1369 pVNC244
NAU6111 pGH242
CLU2331 Gate2BF03 MUSB0100
MUCS188 CIR119
Gate4DD02 BNL3556
UCcg10173_184 UCcg11333_1093 CIR354 CLU87
CLU816 E2M3_261 TMB1640 CLU527
CLU1764 NAU2881 E4M7_161
GA3-hydroxylase1 CLU606
CLU607 HAU2585 CLU527 A1412 CLU1166
BNL3255 E6M16-220
UCcg10246_299
FIF1-800
NAU3201 DOW057
CLU684
CLU1527
TMB0834 UCcg11114_430 Unig22E12 BNL2961
BNL1044 UCcg11327_536
Coau1O15 TMB2279
DPL0646 DPL0176
TMB1966 A1108 pGH244 HAU3211
NAU2214
TMB1675
JESPR039 TMB2929 EM2OD13-105 JESPR046
JESPR035
me1em6-80
NAU1505
A1691 Unig25B12
UCcg11108_240 NAU3605 DPL0031 UCcg10265_529
UCcg10265_340 TMC18-225
CLU1630
CLU4317 Coau1O15 Unig24D03
UCcg10647_235
CLU2331 pAR523 pAR785 Unig27G11 DPL0861
E1M5_85 E2M6_322 UCcg10647_155 TMB2103 E1M3_270
GW55-250 MUSB0780 E8M2_204 NAU6134
BNL1303 UCcg11373_298 NAU6557 TMB2107 NAU4080
HAU3349 GW42-185 NAU3632 CLU1349 BNL2993
EM2GA38-340 TMB1639 HAU0356 em5GA30-55
CLU2116 Y1306-380 NAU1531 CLU1940 NAU5173
NAU3199 UCcot10030_1469 NAU3769
JESPR005 NAU0520 CLU343
em5DC1-300 NAU3058
CLU1676 E1M7_219 Gate2BC04 JESPR090 NAU4010
MUSB1188 Y1325-420
E5M6_145 MUSB0073 em6GA33-165 Unig22C03 CLU995
Gh532 E9M14-230 em6SA12-75
E3M1_212 Gate4BG11 BNL1664 MUSB0662
BNL3474 pAR854 BNL1017 A1828 CLU3
pAR03_15 DPL0457
BNL3255 CLU172 CLU44 G1018 pAR01_40
pAR953 Gate1AB06 Gate3DA02 Unig27F12 Gate2CD01
A1401 A1590 Gate4DA08 M16_200 P05_09
pAR118 pAR798 pGH318 pGH422 BNL3257
BNL0387
MUCS248 CLU1339 P01_02 DPL0755 HAU0672
DPL0113 pAR973 HAU1632 MUSB0442
CLU3257 TMB0029 DPL0220 CLU1290 UCcg10786_208
CS101 CM43 E8M5_430 MUSB1001
TMB1717 E8M6_220 E7M2_95 E3M2_250 E2M3_198
E5M3_240 DPL0839 E5M6_75 E2M7_87 E1M5_167
CIR291
MUSB0818 MUSB0958 E6M6_550 E4M3_106 MUSB0139
BNL2527 CLU77 E7M5_95 E2M4_239 E5M4_410
E4M8_330 E2M4_300
E2M7_170 E2M2_340
HAU1547 UCcg10445_448
HAU1749
CG24
TMB0528 pVNC149
CIR278
HAU1308 BNL3627
CG04 P05_18 pAR03_07 pAR950
E7M16-250
Gafb13B07 Unig06G04
BNL3800 Gate1BF02 Unig25C01
JESPR230 UCcot10157_54 CLU3834 Gate3CH11
Gate1BH09
pAR04_11 HAU1390 UCcot10157_256
NAU0882 Coau2A22
pAR789
CLU1601
Gate1BF03
CIR343 CLU101 A1658 P01_46
NAU1209 CLU13
pAR978 E6M6-130
G1101 Gate2BC08 CLU1207
UCD552 Y1322-1200
E2M6_159 G1078 Gate3CA01 Unig26G07
E14M4-140 MUSS409
pAR123
Gate4DG08 MUSS021
G1276 pAR490
DPL0749 MUCS021
JESPR291
CLU1012
CIR028 NAU3668
NAU3324
CLU4360
NAU3590

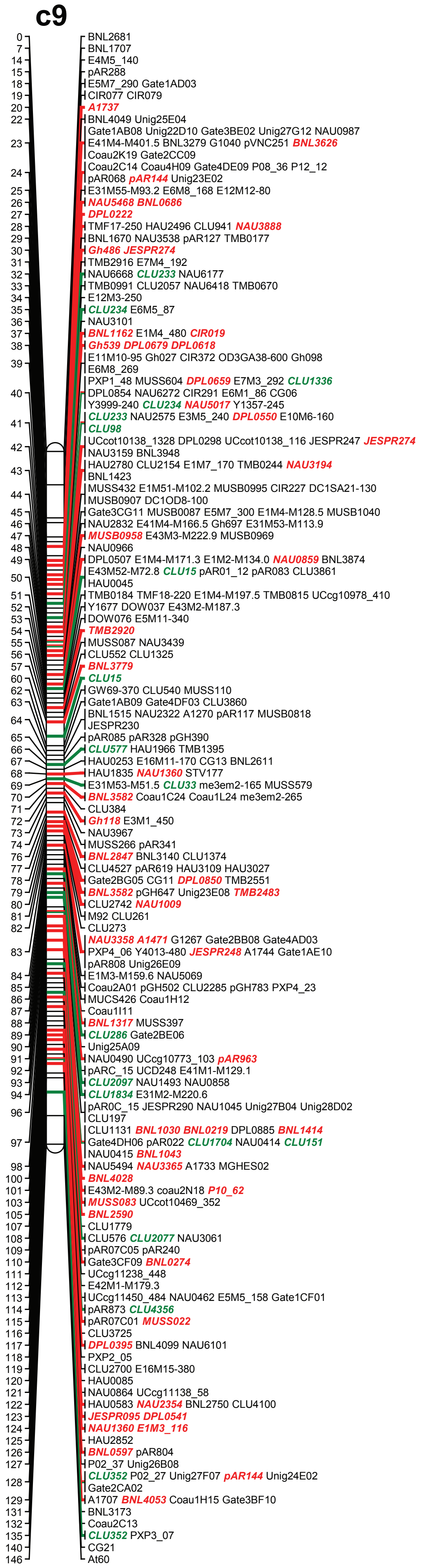
c9
0 7 14 15 18 19 20 22 23 24 25 26 27 28 29 30 31 32 33 34 35 36 37 38 39 40 41 42 43 44 45 46 47 48 49 50 51 52 53 54 55 56 57 60 62 63 64 65 66 67 68 69 70 71 72 73 74 76 77 78 79 80 81 82 83 84 85 86 87 88 89 90 91 92 93 94 96 97 98 100 101 103 105 107 108 109 110 111 112 113 114 115 116 117 118 119 120 121 122 123 124 125 126 127 128 129 131 132 135 140 146
BNL2681
BNL1707
E4M5_140
pAR288
E5M7_290 Gate1AD03
CIR077 CIR079
A1737
BNL4049 Unig25E04
Gate1AB08 Unig22D10 Gate3BE02 Unig27G12 NAU0987
E41M4-M401.5 BNL3279 G1040 pVNC251 BNL3626
Coau2K19 Gate2CC09
Coau2C14 Coau4H09 Gate4DE09 P08_36 P12_12
pAR068 pAR144 Unig23E02
E31M55-M93.2 E6M8_168 E12M12-80
NAU5468 BNL0686
DPL0222
TMF17-250 HAU2496 CLU941 NAU3888
BNL1670 NAU3538 pAR127 TMB0177
Gh486 JESPR274
TMB2916 E7M4_192
NAU6668 CLU233 NAU6177
TMB0991 CLU2057 NAU6418 TMB0670
E12M3-250
CLU234 E6M5_87
NAU3101
BNL1162 E1M4_480 CIR019
Gh539 DPL0679 DPL0618
E11M10-95 Gh027 CIR372 OD3GA38-600 Gh098
E6M8_269
PXP1_48 MUSS604 DPL0659 E7M3_292 CLU1336
DPL0854 NAU6272 CIR291 E6M1_86 CG06
Y3999-240 CLU234 NAU5017 Y1357-245
CLU233 NAU2575 E3M5_240 DPL0550 E10M6-160
CLU98
UCcot10138_1328 DPL0298 UCcot10138_116 JESPR247 JESPR274
NAU3159 BNL3948
HAU2780 CLU2154 E1M7_170 TMB0244 NAU3194
BNL1423
MUSS432 E1M51-M102.2 MUSB0995 CIR227 DC1SA21-130
MUSB0907 DC1OD8-100
Gate3CG11 MUSB0087 E5M7_300 E1M4-M128.5 MUSB1040
NAU2832 E41M4-M166.5 Gh697 E31M53-M113.9
MUSB0958 E43M3-M222.9 MUSB0969
NAU0966
DPL0507 E1M4-M171.3 E1M2-M134.0 NAU0859 BNL3874
E43M52-M72.8 CLU15 pAR01_12 pAR083 CLU3861
HAU0045
TMB0184 TMF18-220 E1M4-M197.5 TMB0815 UCcg10978_410
Y1677 DOW037 E43M2-M187.3
DOW076 E5M11-340
TMB2920
MUSS087 NAU3439
CLU552 CLU1325
BNL3779
CLU15
GW69-370 CLU540 MUSS110
Gate1AB09 Gate4DF03 CLU3860
BNL1515 NAU2322 A1270 pAR117 MUSB0818
JESPR230
pAR085 pAR328 pGH390
CLU577 HAU1966 TMB1395
HAU0253 E16M11-170 CG13 BNL2611
HAU1835 NAU1360 STV177
E31M53-M51.5 CLU33 me3em2-165 MUSS579
BNL3582 Coau1C24 Coau1L24 me3em2-265
CLU384
Gh118 E3M1_450
NAU3967
MUSS266 pAR341
BNL2847 BNL3140 CLU1374
CLU4527 pAR619 HAU3109 HAU3027
Gate2BG05 CG11 DPL0850 TMB2551
BNL3582 pGH647 Unig23E08 TMB2483
CLU2742 NAU1009
M92 CLU261
CLU273
NAU3358 A1471 G1267 Gate2BB08 Gate4AD03
PXP4_06 Y4013-480 JESPR248 A1744 Gate1AE10
pAR808 Unig26E09
E1M3-M159.6 NAU5069
Coau2A01 pGH502 CLU2285 pGH783 PXP4_23
MUCS426 Coau1H12
Coau1I11
BNL1317 MUSS397
CLU286 Gate2BE06
Unig25A09
NAU0490 UCcg10773_103 pAR963
pARC_15 UCD248 E41M1-M129.1
CLU2097 NAU1493 NAU0858
CLU1834 E31M2-M220.6
pAR0C_15 JESPR290 NAU1045 Unig27B04 Unig28D02
CLU197
CLU1131 BNL1030 BNL0219 DPL0885 BNL1414
Gate4DH06 pAR022 CLU1704 NAU0414 CLU151
NAU0415 BNL1043
NAU5494 NAU3365 A1733 MGHES02
BNL4028
E43M2-M89.3 coau2N18 P10_62
MUSS083 UCcot10469_352
BNL2590
CLU1779
CLU576 CLU2077 NAU3061
pAR07C05 pAR240
Gate3CF09 BNL0274
UCcg11238_448
E42M1-M179.3
UCcg11450_484 NAU0462 E5M5_158 Gate1CF01
pAR873 CLU4356
pAR07C01 MUSS022
CLU3725
DPL0395 BNL4099 NAU6101
PXP2_05
CLU2700 E16M15-380
HAU0085
NAU0864 UCcg11138_58
HAU0583 NAU2354 BNL2750 CLU4100
JESPR095 DPL0541
NAU1360 E1M3_116
HAU2852
BNL0597 pAR804
P02_37 Unig26B08
CLU352 P02_27 Unig27F07 pAR144 Unig24E02
Gate2CA02
A1707 BNL4053 Coau1H15 Gate3BF10
BNL3173
Coau2C13
CLU352 PXP3_07
CG21
At60

c10

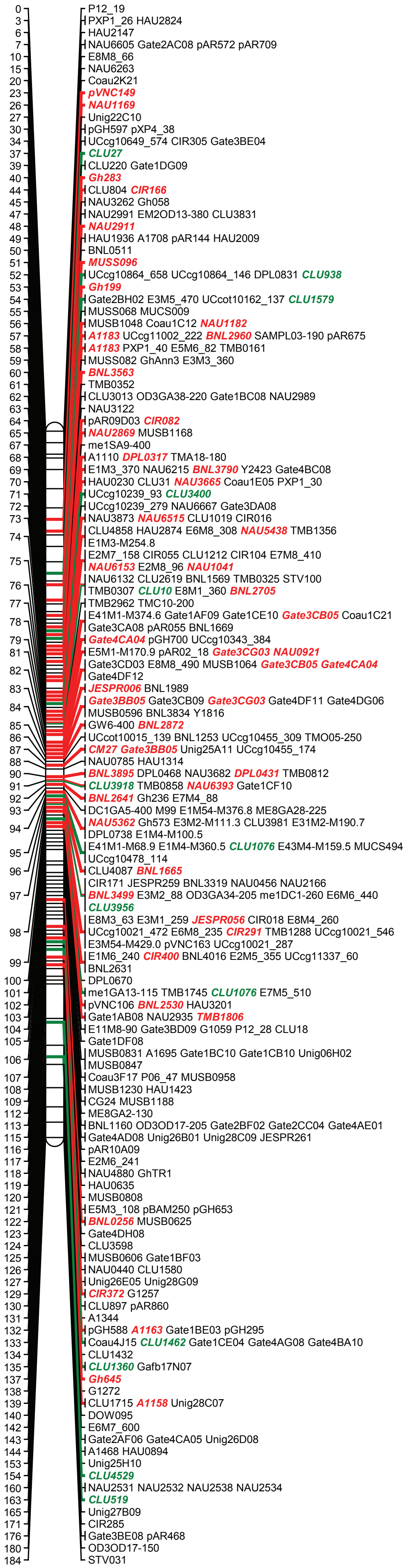

0
3
6
7
10
15
20
23
26
27
30
34
37
39
40
44
45
47
48
49
50
51
52
53
54
55
56
57
58
59
60
61
62
63
64
65
67
68
69
70
71
72
73
74
75
76
77
78
79
81
82
83
84
85
86
87
88
90
91
92
93
94
95
96
97
98
99
100
101
102
103
104
105
106
107
108
109
112
113
115
116
117
118
119
120
121
122
123
124
125
126
127
129
130
131
132
133
134
135
137
138
139
140
142
143
144
153
154
160
163
165
171
176
180
184
P12_19
PXP1_26 HAU2824
HAU2147
NAU6605 Gate2AC08 pAR572 pAR709
E8M8_66
NAU6263
Coau2K21
pVNC149
NAU1169
Unig22C10
pGH597 pXP4_38
UCcg10649_574 CIR305 Gate3BE04
CLU27
CLU220 Gate1DG09
Gh283
CLU804 CIR166
NAU3262 Gh058
NAU2991 EM2OD13-380 CLU3831
NAU2911
HAU1936 A1708 pAR144 HAU2009
BNL0511
MUSS096
UCcg10864_658 UCcg10864_146 DPL0831 CLU938
Gh199
Gate2BH02 E3M5_470 UCcot10162_137 CLU1579
MUSS068 MUCS009
MUSB1048 Coau1C12 NAU1182
A1183 UCcg11002_222 BNL2960 SAMPL03-190 pAR675
A1183 PXP1_40 E5M6_82 TMB0161
MUSS082 GhAnn3 E3M3_360
BNL3563
TMB0352
CLU3013 OD3GA38-220 Gate1BC08 NAU2989
NAU3122
pAR09D03 CIR082
NAU2869 MUSB1168
me1SA9-400
A1110 DPL0317 TMA18-180
E1M3_370 NAU6215 BNL3790 Y2423 Gate4BC08
HAU0230 CLU31 NAU3665 Coau1E05 PXP1_30
UCcg10239_93 CLU3400
UCcg10239_279 NAU6667 Gate3DA08
NAU3873 NAU6515 CLU1019 CIR016
CLU4858 HAU2874 E6M8_308 NAU5438 TMB1356
E1M3-M254.8
E2M7_158 CIR055 CLU1212 CIR104 E7M8_410
NAU6153 E2M8_96 NAU1041
NAU6132 CLU2619 BNL1569 TMB0325 STV100
TMB0307 CLU10 E8M1_360 BNL2705
TMB2962 TMC10-200
E41M1-M374.6 Gate1AF09 Gate1CE10 Gate3CB05 Coau1C21
Gate3CA08 pAR055 BNL1669
Gate4CA04 pGH700 UCcg10343_384
E5M1-M170.9 pAR02_18 Gate3CG03 NAU0921
Gate3CD03 E8M8_490 MUSB1064 Gate3CB05 Gate4CA04
Gate4DF12
JESPR006 BNL1989
Gate3BB05 Gate3CB09 Gate3CG03 Gate4DF11 Gate4DG06
MUSB0596 BNL3834 Y1816
GW6-400 BNL2872
UCcot10015_139 BNL1253 UCcg10455_309 TMO05-250
CM27 Gate3BB05 Unig25A11 UCcg10455_174
NAU0785 HAU1314
BNL3895 DPL0468 NAU3682 DPL0431 TMB0812
CLU3918 TMB0858 NAU6393 Gate1CF10
BNL2641 Gh236 E7M4_88
DC1GA5-400 M99 E1M54-M376.8 ME8GA28-225
NAU5362 Gh573 E3M2-M111.3 CLU3981 E31M2-M190.7
DPL0738 E1M4-M100.5
E41M1-M68.9 E1M4-M360.5 CLU1076 E43M4-M159.5 MUCS494
UCcg10478_114
CLU4087 BNL1665
CIR171 JESPR259 BNL3319 NAU0456 NAU2166
BNL3499 E3M2_88 OD3GA34-205 me1DC1-260 E6M6_440
CLU3956
E8M3_63 E3M1_259 JESPR056 CIR018 E8M4_260
UCcg10021_472 E6M8_235 CIR291 TMB1288 UCcg10021_546
E3M54-M429.0 pVNC163 UCcg10021_287
E1M6_240 CIR400 BNL4016 E2M5_355 UCcg11337_60
BNL2631
DPL0670
me1GA13-115 TMB1745 CLU1076 E7M5_510
pVNC106 BNL2530 HAU3201
Gate1AB08 NAU2935 TMB1806
E11M8-90 Gate3BD09 G1059 P12_28 CLU18
Gate1DF08
MUSB0831 A1695 Gate1BC10 Gate1CB10 Unig06H02
MUSB0847
Coau3F17 P06_47 MUSB0958
MUSB1230 HAU1423
CG24 MUSB1188
ME8GA2-130
BNL1160 OD3OD17-205 Gate2BF02 Gate2CC04 Gate4AE01
Gate4AD08 Unig26B01 Unig28C09 JESPR261
pAR10A09
E2M6_241
NAU4880 GhTR1
HAU0635
MUSB0808
E5M3_108 pBAM250 pGH653
BNL0256 MUSB0625
Gate4DH08
CLU3598
MUSB0606 Gate1BF03
NAU0440 CLU1580
Unig26E05 Unig28G09
CIR372 G1257
CLU897 pAR860
A1344
pGH588 A1163 Gate1BE03 pGH295
Coau4J15 CLU1462 Gate1CE04 Gate4AG08 Gate4BA10
CLU1432
CLU1360 Gafb17N07
Gh645
G1272
CLU1715 A1158 Unig28C07
DOW095
E6M7_600
Gate2AF06 Gate4CA05 Unig26D08
A1468 HAU0894
Unig25H10
CLU4529
NAU2531 NAU2532 NAU2538 NAU2534
CLU519
Unig27B09
CIR285
Gate3BE08 pAR468
OD3OD17-150
STV031

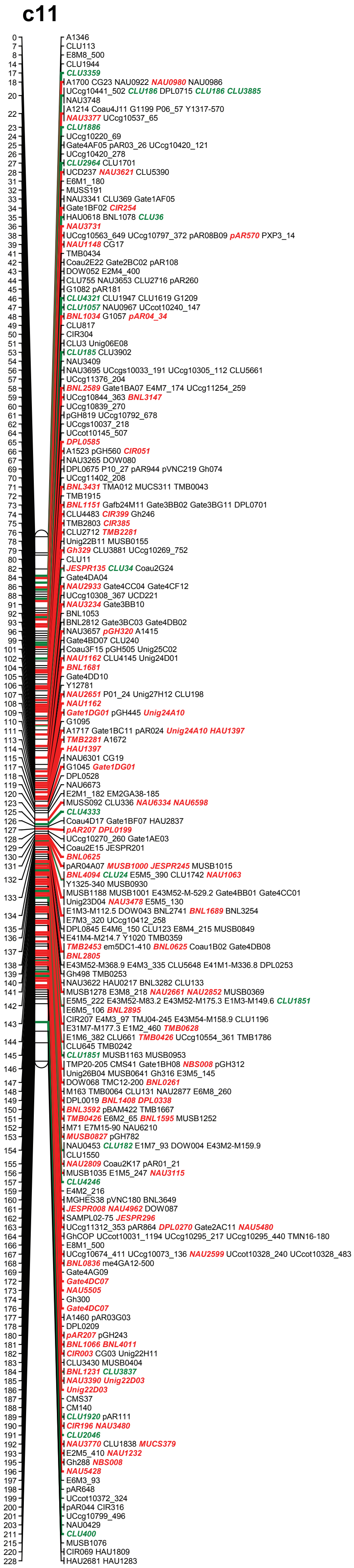
c11
0
7
8
14
17
18
20
22
23
24
25
26
27
28
31
32
33
34
35
36
38
39
41
42
43
44
45
46
47
48
49
50
51
53
54
56
57
58
59
60
61
62
64
65
66
67
69
70
71
72
73
74
75
76
78
79
80
82
84
86
88
91
92
93
96
99
101
102
104
105
106
107
108
109
110
111
113
114
115
117
118
119
120
123
125
126
127
128
129
130
131
132
133
134
135
136
137
138
139
140
141
142
143
144
145
146
147
148
149
150
151
152
153
154
155
156
157
159
160
161
162
163
164
166
167
168
169
172
173
174
176
177
178
180
181
182
183
184
185
186
187
188
189
190
191
192
193
195
196
197
198
199
200
201
203
211
215
220
228
A1346
CLU113
E8M8_500
CLU1944
CLU3359
A1700 CG23 NAU0922 NAU0980 NAU0986
UCcg10441_502 CLU186 DPL0715 CLU186 CLU3885
NAU3748
A1214 Coau4J11 G1199 P06_57 Y1317-570
NAU3377 UCcg10537_65
CLU1886
UCcg10220_69
Gate4AF05 pAR03_26 UCcg10420_121
UCcg10420_278
CLU2964 CLU1701
UCD237 NAU3621 CLU5390
E6M1_180
MUSS191
NAU3341 CLU369 Gate1AF05
Gate1BF02 CIR254
HAU0618 BNL1078 CLU36
NAU3731
UCcg10563_649 UCcg10797_372 pAR08B09 pAR570 PXP3_14
NAU1148 CG17
TMB0434
Coau2E22 Gate2BC02 pAR108
DOW052 E2M4_400
CLU755 NAU3653 CLU2716 pAR260
G1082 pAR181
CLU4321 CLU1947 CLU1619 G1209
CLU1057 NAU0967 UCcot10240_147
BNL1034 G1057 pAR04_34
CLU817
CIR304
CLU3 Unig06E08
CLU185 CLU3902
NAU3409
NAU3695 UCcgs10033_191 UCcg10305_112 CLU5661
UCcg11376_204
BNL2589 Gate1BA07 E4M7_174 UCcg11254_259
UCcg10844_363 BNL3147
UCcg10839_270
pGH819 UCcg10792_678
UCcgs10037_218
UCcot10145_507
DPL0585
A1523 pGH560 CIR051
NAU3265 DOW080
DPL0675 P10_27 pAR944 pVNC219 Gh074
UCcg11402_208
BNL3431 TMA012 MUCS311 TMB0043
TMB1915
BNL1151 Gafb24M11 Gate3BB02 Gate3BG11 DPL0701
CLU4483 CIR399 Gh246
TMB2803 CIR385
CLU2712 TMB2281
Unig22B11 MUSB0155
Gh329 CLU3881 UCcg10269_752
CLU11
JESPR135 CLU34 Coau2G24
Gate4DA04
NAU2933 Gate4CC04 Gate4CF12
UCcg10308_367 UCD221
NAU3234 Gate3BB10
BNL1053
BNL2812 Gate3BC03 Gate4DB02
NAU3657 pGH320 A1415
Gate4BD07 CLU240
Coau3F15 pGH505 Unig25C02
NAU1162 CLU4145 Unig24D01
BNL1681
Gate4DD10
Y12781
NAU2651 P01_24 Unig27H12 CLU198
NAU1162
Gate1DG01 pGH445 Unig24A10
G1095
A1717 Gate1BC11 pAR024 Unig24A10 HAU1397
TMB2281 A1672
HAU1397
NAU6301 CG19
G1045 Gate1DG01
DPL0528
NAU6673
E2M1_182 EM2GA38-185
MUSS092 CLU336 NAU6334 NAU6598
CLU4333
Coau4D17 Gate1BF07 HAU2837
pAR207 DPL0199
UCcg10270_260 Gate1AE03
Coau2E15 JESPR201
BNL0625
pAR04A07 MUSB1000 JESPR245 MUSB1015
BNL4094 CLU24 E5M5_390 CLU1742 NAU1063
Y1325-340 MUSB0930
MUSB1188 MUSB1001 E43M52-M-529.2 Gate4BB01 Gate4CC01
Unig23D04 NAU3478 E5M5_130
E1M3-M112.5 DOW043 BNL2741 BNL1689 BNL3254
E7M3_320 UCcg10412_258
DPL0845 E4M6_150 CLU123 E8M4_215 MUSB0849
E41M4-M214.7 Y1020 TMB0359
TMB2453 em5DC1-410 BNL0625 Coau1B02 Gate4DB08
BNL2805
E43M52-M368.9 E4M3_335 CLU5648 E41M1-M336.8 DPL0253
Gh498 TMB0253
NAU3622 HAU0217 BNL3282 CLU133
MUSB1278 E3M8_218 NAU2661 NAU2852 MUSB0369
E5M5_222 E43M52-M83.2 E43M52-M175.3 E1M3-M149.6 CLU1851
E6M5_106 BNL2895
CIR207 E4M3_97 TMJ04-245 E43M54-M158.9 CLU1196
E31M7-M177.3 E1M2_460 TMB0628
E1M6_382 CLU661 TMB0426 UCcg10554_361 TMB1786
CLU645 TMB0242
CLU1851 MUSB1163 MUSB0953
TMP20-205 CMS41 Gate1BH08 NBS008 pGH312
Unig26B04 MUSB0641 Gh316 E3M5_145
DOW068 TMC12-200 BNL0261
M163 TMB0064 CLU131 NAU2877 E6M8_260
DPL0019 BNL1408 DPL0338
BNL3592 pBAM422 TMB1667
TMB0426 E6M2_65 BNL1595 MUSB1252
M71 E7M15-90 NAU6210
MUSB0827 pGH782
NAU0453 CLU182 E1M7_93 DOW004 E43M2-M159.9
CLU1550
NAU2809 Coau2K17 pAR01_21
MUSB1035 E1M5_247 NAU3115
CLU4246
E4M2_216
MGHES38 pVNC180 BNL3649
JESPR008 NAU4962 DOW087
SAMPL02-75 JESPR296
UCcg11312_353 pAR864 DPL0270 Gate2AC11 NAU5480
GhCOP UCcot10031_1194 UCcg10295_217 UCcg10295_440 TMN16-180
E8M1_500
UCcg10674_411 UCcg10073_136 NAU2599 UCcot10328_240 UCcot10328_483
BNL0836 me4GA12-500
Gate4AG09
Gate4DC07
NAU5505
Gh300
Gate4DC07
A1460 pAR03G03
DPL0209
pAR207 pGH243
BNL1066 BNL4011
CIR003 CG03 Unig22H11
CLU3430 MUSB0404
BNL1231 CLU3837
NAU3390 Unig22D03
Unig22D03
CMS37
CM140
CLU1920 pAR111
CIR196 NAU3480
CLU2046
NAU3770 CLU1838 MUCS379
E2M5_410 NAU1232
Gh288 NBS008
NAU5428
E6M3_93
pAR648
UCcot10372_324
pAR044 CIR316
UCcg10799_496
NAU0429
CLU400
MUSB1076
CIR069 HAU1809
HAU2681 HAU1283

c12

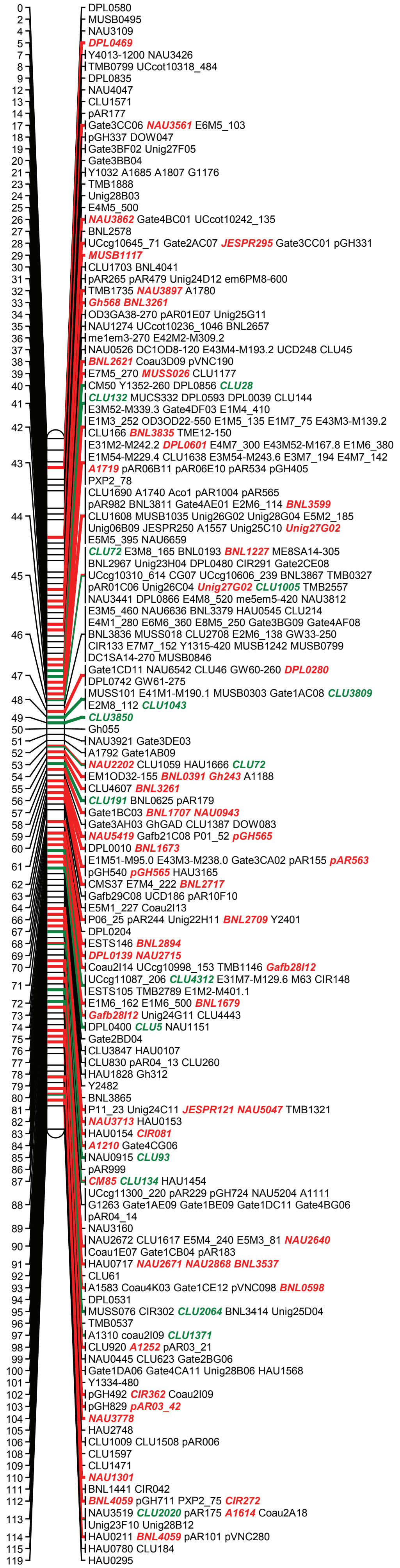

0 2 4 5 7 8 9 12 13 14 17 18 19 20 21 23 24 25 26 27 28 29 30 31 32 33 34 35 36 37 38 39 40 41 42 43 44 45 46 47 48 49 50 51 52 53 54 55 56 57 58 59 60 61 62 63 64 66 67 68 69 70 71 72 73 74 75 76 77 78 79 80 81 82 83 84 85 86 87 88 89 90 91 92 93 94 95 96 97 98 99 100 101 102 103 104 105 106 108 109 110 111 112 113 114 115 119
DPL0580
MUSB0495
NAU3109
DPL0469
Y4013-1200 NAU3426
TMB0799 UCcot10318_484
DPL0835
NAU4047
CLU1571
pAR177
Gate3CC06 NAU3561 E6M5_103
pGH337 DOW047
Gate3BF02 Unig27F05
Gate3BB04
Y1032 A1685 A1807 G1176
TMB1888
Unig28B03
E4M5_500
NAU3862 Gate4BC01 UCcot10242_135
BNL2578
UCcg10645_71 Gate2AC07 JESPR295 Gate3CC01 pGH331
MUSB1117
CLU1703 BNL4041
pAR265 pAR479 Unig24D12 em6PM8-600
TMB1735 NAU3897 A1780
Gh568 BNL3261
OD3GA38-270 pAR01E07 Unig25G11
NAU1274 UCcot10236_1046 BNL2657
me1em3-270 E42M2-M309.2
NAU0526 DC1OD8-120 E43M4-M193.2 UCD248 CLU45
BNL2621 Coau3D09 pVNC190
E7M5_270 MUSS026 CLU1177
CM50 Y1352-260 DPL0856 CLU28
CLU132 MUCS332 DPL0593 DPL0039 CLU144
E3M52-M339.3 Gate4DF03 E1M4_410
E1M3_252 OD3OD22-550 E1M5_135 E1M7_75 E43M3-M139.2
CLU166 BNL3835 TME12-150
E31M2-M242.2 DPL0601 E4M7_300 E43M52-M167.8 E1M6_380
E1M54-M229.4 CLU1638 E3M54-M243.6 E3M7_194 E4M7_142
A1719 pAR06B11 pAR06E10 pAR534 pGH405
PXP2_78
CLU1690 A1740 Aco1 pAR1004 pAR565
pAR982 BNL3811 Gate4AE01 E2M6_114 BNL3599
CLU1608 MUSB1035 Unig26G02 Unig28G04 E5M2_185
Unig06B09 JESPR250 A1557 Unig25C10 Unig27G02
E5M5_395 NAU6659
CLU72 E3M8_165 BNL0193 BNL1227 ME8SA14-305
BNL2967 Unig23H04 DPL0480 CIR291 Gate2CE08
UCcg10310_614 CG07 UCcg10606_239 BNL3867 TMB0327
pAR01C06 Unig26C04 Unig27G02 CLU1005 TMB2557
NAU3441 DPL0866 E4M8_520 me5em5-420 NAU3812
E3M5_460 NAU6636 BNL3379 HAU0545 CLU214
E4M1_280 E6M6_360 E8M5_250 Gate3BG09 Gate4AF08
BNL3836 MUSS018 CLU2708 E2M6_138 GW33-250
CIR133 E7M7_152 Y1315-420 MUSB1242 MUSB0799
DC1SA14-270 MUSB0846
Gate1CD11 NAU6542 CLU46 GW60-260 DPL0280
DPL0742 GW61-275
MUSS101 E41M1-M190.1 MUSB0303 Gate1AC08 CLU3809
E2M8_112 CLU1043
CLU3850
Gh055
NAU3921 Gate3DE03
A1792 Gate1AB09
NAU2202 CLU1059 HAU1666 CLU72
EM1OD32-155 BNL0391 Gh243 A1188
CLU4607 BNL3261
CLU191 BNL0625 pAR179
Gate1BC03 BNL1707 NAU0943
Gate3AH03 GhGAD CLU1387 DOW083
NAU5419 Gafb21C08 P01_52 pGH565
DPL0010 BNL1673
E1M51-M95.0 E43M3-M238.0 Gate3CA02 pAR155 pAR563
pGH540 pGH565 HAU3165
CMS37 E7M4_222 BNL2717
Gafb29C08 UCD186 pAR10F10
E5M1_227 Coau2I13
P06_25 pAR244 Unig22H11 BNL2709 Y2401
DPL0204
ESTS146 BNL2894
DPL0139 NAU2715
Coau2I14 UCcg10998_153 TMB1146 Gafb28I12
UCcg11087_206 CLU4312 E31M7-M129.6 M63 CIR148
ESTS105 TMB2789 E1M2-M401.1
E1M6_162 E1M6_500 BNL1679
Gafb28I12 Unig24G11 CLU4443
DPL0400 CLU5 NAU1151
Gate2BD04
CLU3847 HAU0107
CLU830 pAR04_13 CLU260
HAU1828 Gh312
Y2482
BNL3865
P11_23 Unig24C11 JESPR121 NAU5047 TMB1321
NAU3713 HAU0153
HAU0154 CIR081
A1210 Gate4CG06
NAU0915 CLU93
pAR999
CM85 CLU134 HAU1454
UCcg11300_220 pAR229 pGH724 NAU5204 A1111
G1263 Gate1AE09 Gate1BE09 Gate1DC11 Gate4BG06
pAR04_14
NAU3160
NAU2672 CLU1617 E5M4_240 E5M3_81 NAU2640
Coau1E07 Gate1CB04 pAR183
HAU0717 NAU2671 NAU2868 BNL3537
CLU61
A1583 Coau4K03 Gate1CE12 pVNC098 BNL0598
DPL0531
MUSS076 CIR302 CLU2064 BNL3414 Unig25D04
TMB0537
A1310 coau2I09 CLU1371
CLU920 A1252 pAR03_21
NAU0445 CLU623 Gate2BG06
Gate1DA06 Gate4CA11 Unig28B06 HAU1568
Y1334-480
pGH492 CIR362 Coau2I09
pGH829 pAR03_42
NAU3778
HAU2748
CLU1009 CLU1508 pAR006
CLU1597
CLU1471
NAU1301
BNL1441 CIR042
BNL4059 pGH711 PXP2_75 CIR272
NAU3519 CLU2020 pAR175 A1614 Coau2A18
Unig23F10 Unig28B12
HAU0211 BNL4059 pAR101 pVNC280
HAU0780 CLU184
HAU0295

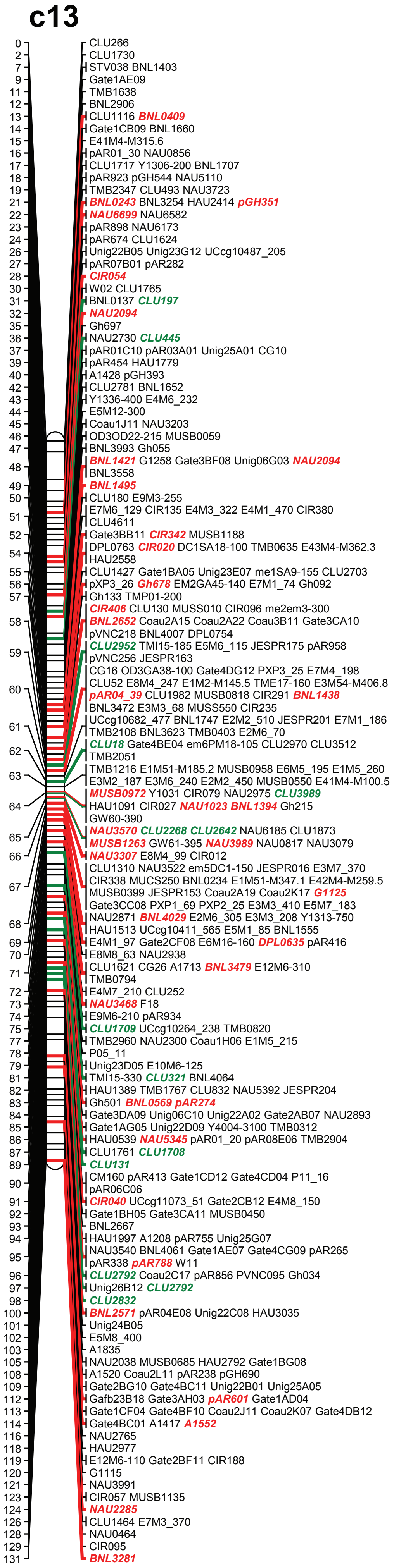
c13
0
2
7
9
11
12
13
14
15
16
17
18
19
21
22
23
24
26
27
28
30
31
32
35
36
37
39
40
42
43
44
45
46
47
48
49
50
51
52
54
55
56
57
58
59
60
61
62
63
64
65
66
67
68
69
70
71
72
73
74
75
77
78
79
81
82
83
84
85
86
87
89
90
91
92
93
94
95
96
97
98
100
101
102
103
105
108
109
112
113
114
116
118
119
120
121
123
124
126
128
129
131
CLU266
CLU1730
STV038 BNL1403
Gate1AE09
TMB1638
BNL2906
CLU1116 BNL0409
Gate1CB09 BNL1660
E41M4-M315.6
pAR01_30 NAU0856
CLU1717 Y1306-200 BNL1707
pAR923 pGH544 NAU5110
TMB2347 CLU493 NAU3723
BNL0243 BNL3254 HAU2414 pGH351
NAU6699 NAU6582
pAR898 NAU6173
pAR674 CLU1624
Unig22B05 Unig23G12 UCcg10487_205
pAR07B01 pAR282
CIR054
W02 CLU1765
BNL0137 CLU197
NAU2094
Gh697
NAU2730 CLU445
pAR01C10 pAR03A01 Unig25A01 CG10
pAR454 HAU1779
A1428 pGH393
CLU2781 BNL1652
Y1336-400 E4M6_232
E5M12-300
Coau1J11 NAU3203
OD3OD22-215 MUSB0059
BNL3993 Gh055
BNL1421 G1258 Gate3BF08 Unig06G03 NAU2094
BNL3558
BNL1495
CLU180 E9M3-255
E7M6_129 CIR135 E4M3_322 E4M1_470 CIR380
CLU4611
Gate3BB11 CIR342 MUSB1188
DPL0763 CIR020 DC1SA18-100 TMB0635 E43M4-M362.3
HAU2558
CLU1427 Gate1BA05 Unig23E07 me1SA9-155 CLU2703
pXP3_26 Gh678 EM2GA45-140 E7M1_74 Gh092
Gh133 TMP01-200
CIR406 CLU130 MUSS010 CIR096 me2em3-300
BNL2652 Coau2A15 Coau2A22 Coau3B11 Gate3CA10
pVNC218 BNL4007 DPL0754
CLU2952 TMI15-185 E5M6_115 JESPR175 pAR958
pVNC256 JESPR163
CG16 OD3GA38-100 Gate4DG12 PXP3_25 E7M4_198
CLU52 E8M4_247 E1M2-M145.5 TME17-160 E3M54-M406.8
pAR04_39 CLU1982 MUSB0818 CIR291 BNL1438
BNL3472 E3M3_68 MUSS550 CIR235
UCcg10682_477 BNL1747 E2M2_510 JESPR201 E7M1_186
TMB2108 BNL3623 TMB0403 E2M6_70
CLU18 Gate4BE04 em6PM18-105 CLU2970 CLU3512
TMB2051
TMB1216 E1M51-M185.2 MUSB0958 E6M5_195 E1M5_260
E3M2_187 E3M6_240 E2M2_450 MUSB0550 E41M4-M100.5
MUSB0972 Y1031 CIR079 NAU2975 CLU3989
HAU1091 CIR027 NAU1023 BNL1394 Gh215
GW60-390
NAU3570 CLU2268 CLU2642 NAU6185 CLU1873
MUSB1263 GW61-395 NAU3989 NAU0817 NAU3079
NAU3307 E8M4_99 CIR012
CLU1310 NAU3522 em5DC1-150 JESPR016 E3M7_370
CIR338 MUCS250 BNL0234 E1M51-M347.1 E42M4-M259.5
MUSB0399 JESPR153 Coau2A19 Coau2K17 G1125
Gate3CC08 PXP1_69 PXP2_25 E3M3_410 E5M7_183
NAU2871 BNL4029 E2M6_305 E3M3_208 Y1313-750
HAU1513 UCcg10411_565 E5M1_85 BNL1555
E4M1_97 Gate2CF08 E6M16-160 DPL0635 pAR416
E8M8_63 NAU2938
CLU1621 CG26 A1713 BNL3479 E12M6-310
TMB0794
E4M7_210 CLU252
NAU3468 F18
E9M6-210 pAR934
CLU1709 UCcg10264_238 TMB0820
TMB2960 NAU2300 Coau1H06 E1M5_215
P05_11
Unig23D05 E10M6-125
TMI15-330 CLU321 BNL4064
HAU1389 TMB1767 CLU832 NAU5392 JESPR204
Gh501 BNL0569 pAR274
Gate3DA09 Unig06C10 Unig22A02 Gate2AB07 NAU2893
Gate1AG05 Unig22D09 Y4004-3100 TMB0312
HAU0539 NAU5345 pAR01_20 pAR08E06 TMB2904
CLU1761 CLU1708
CLU131
CM160 pAR413 Gate1CD12 Gate4CD04 P11_16
pAR06C06
CIR040 UCcg11073_51 Gate2CB12 E4M8_150
Gate1BH05 Gate3CA11 MUSB0450
BNL2667
HAU1997 A1208 pAR755 Unig25G07
NAU3540 BNL4061 Gate1AE07 Gate4CG09 pAR265
pAR338 pAR788 W11
CLU2792 Coau2C17 pAR856 PVNC095 Gh034
Unig26B12 CLU2792
CLU2832
BNL2571 pAR04E08 Unig22C08 HAU3035
Unig24B05
E5M8_400
A1835
NAU2038 MUSB0685 HAU2792 Gate1BG08
A1520 Coau2L11 pAR238 pGH690
Gate2BG10 Gate4BC11 Unig22B01 Unig25A05
Gafb23B18 Gate3AH03 pAR601 Gate1AD04
Gate1CF04 Gate4BF10 Coau2J11 Coau2K07 Gate4DB12
Gate4BC01 A1417 A1552
NAU2765
HAU2977
E12M6-110 Gate2BF11 CIR188
G1115
NAU3991
CIR057 MUSB1135
NAU2285
CLU1464 E7M3_370
NAU0464
CIR095
BNL3281

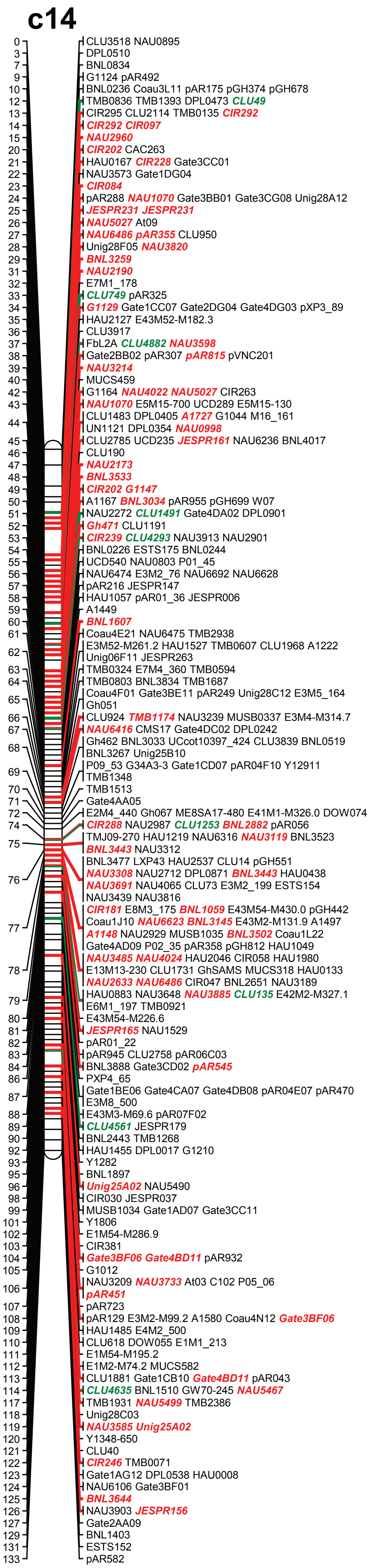
c14
0 3 7 9 10 12 13 14 15 20 21 22 23 24 25 26 27 28 29 31 32 33 34 35 36 37 38 39 40 42 43 44 45 46 47 48 49 50 51 52 53 54 55 56 57 58 59 60 61 62 63 64 65 66 67 68 69 70 71 72 74 75 76 77 78 79 80 81 82 83 84 86 87 88 89 90 92 93 95 96 98 99 101 102 103 104 105 106 107 108 109 110 111 112 113 114 117 118 119 120 121 122 123 124 125 126 127 129 131 133
CLU3518 NAU0895
DPL0510
BNL0834
G1124 pAR492
BNL0236 Coau3L11 pAR175 pGH374 pGH678
TMB0836 TMB1393 DPL0473 CLU49
CIR295 CLU2114 TMB0135 CIR292
CIR292 CIR097
NAU2960
CIR202 CAC263
HAU0167 CIR228 Gate3CC01
NAU3573 Gate1DG04
CIR084
pAR288 NAU1070 Gate3BB01 Gate3CG08 Unig28A12
JESPR231 JESPR231
NAU5027 At09
NAU6486 pAR355 CLU950
Unig28F05 NAU3820
BNL3259
NAU2190
E7M1_178
CLU749 pAR325
G1129 Gate1CC07 Gate2DG04 Gate4DG03 pXP3_89
HAU2127 E43M52-M182.3
CLU3917
FbL2A CLU4882 NAU3598
Gate2BB02 pAR307 pAR815 pVNC201
NAU3214
MUCS459
G1164 NAU4022 NAU5027 CIR263
NAU1070 E5M15-700 UCD289 E5M15-130
CLU1483 DPL0405 A1727 G1044 M16_161
UN1121 DPL0354 NAU0998
CLU2785 UCD235 JESPR161 NAU6236 BNL4017
CLU190
NAU2173
BNL3533
CIR202 G1147
A1167 BNL3034 pAR955 pGH699 W07
NAU2272 CLU1491 Gate4DA02 DPL0901
Gh471 CLU1191
CIR239 CLU4293 NAU3913 NAU2901
BNL0226 ESTS175 BNL0244
UCD540 NAU0803 P01_45
NAU6474 E3M2_76 NAU6692 NAU6628
pAR216 JESPR147
HAU1057 pAR01_36 JESPR006
A1449
BNL1607
Coau4E21 NAU6475 TMB2938
E3M52-M261.2 HAU1527 TMB0607 CLU1968 A1222
Unig06F11 JESPR263
TMB0324 E7M4_360 TMB0594
TMB0803 BNL3834 TMB1687
Coau4F01 Gate3BE11 pAR249 Unig28C12 E3M5_164
Gh051
CLU924 TMB1174 NAU3239 MUSB0337 E3M4-M314.7
NAU6416 CMS17 Gate4DC02 DPL0242
Gh462 BNL3033 UCcot10397_424 CLU3839 BNL0519
BNL3267 Unig25B10
P09_53 G34A3-3 Gate1CD07 pAR04F10 Y12911
TMB1348
TMB1513
Gate4AA05
E2M4_440 Gh067 ME8SA17-480 E41M1-M326.0 DOW074
CIR288 NAU2987 CLU1253 BNL2882 pAR056
TMJ09-270 HAU1219 NAU6316 NAU3119 BNL3523
BNL3443 NAU3312
BNL3477 LXP43 HAU2537 CLU14 pGH551
NAU3308 NAU2712 DPL0871 BNL3443 HAU0438
NAU3691 NAU4065 CLU73 E3M2_199 ESTS154
NAU3439 NAU3816
CIR181 E8M3_175 BNL1059 E43M54-M430.0 pGH442
Coau1J10 NAU6623 BNL3145 E43M2-M131.9 A1497
A1148 NAU2929 MUSB1035 BNL3502 Coau1L22
Gate4AD09 P02_35 pAR358 pGH812 HAU1049
NAU3485 NAU4024 HAU2046 CIR058 HAU1980
E13M13-230 CLU1731 GhSAMS MUCS318 HAU0133
NAU2633 NAU6486 CIR047 BNL2651 NAU3189
HAU0883 NAU3648 NAU3885 CLU135 E42M2-M327.1
E6M1_197 TMB0921
E43M54-M226.6
JESPR165 NAU1529
pAR01_22
pAR945 CLU2758 pAR06C03
BNL3888 Gate3CD02 pAR545
PXP4_65
Gate1BE06 Gate4CA07 Gate4DB08 pAR04E07 pAR470
E3M8_500
E43M3-M69.6 pAR07F02
CLU4561 JESPR179
BNL2443 TMB1268
HAU1455 DPL0017 G1210
Y1282
BNL1897
Unig25A02 NAU5490
CIR030 JESPR037
MUSB1034 Gate1AD07 Gate3CC11
Y1806
E1M54-M286.9
CIR381
Gate3BF06 Gate4BD11 pAR932
G1012
NAU3209 NAU3733 At03 C102 P05_06
pAR451
pAR723
pAR129 E3M2-M99.2 A1580 Coau4N12 Gate3BF06
HAU1485 E4M2_500
CLU618 DOW055 E1M1_213
E1M54-M195.2
E1M2-M74.2 MUCS582
CLU1881 Gate1CB10 Gate4BD11 pAR043
CLU4635 BNL1510 GW70-245 NAU5467
TMB1931 NAU5499 TMB2386
Unig28C03
NAU3585 Unig25A02
Y1348-650
CLU40
CIR246 TMB0071
Gate1AG12 DPL0538 HAU0008
NAU6106 Gate3BF01
BNL3644
NAU3903 JESPR156
Gate2AA09
BNL1403
ESTS152
pAR582

c15

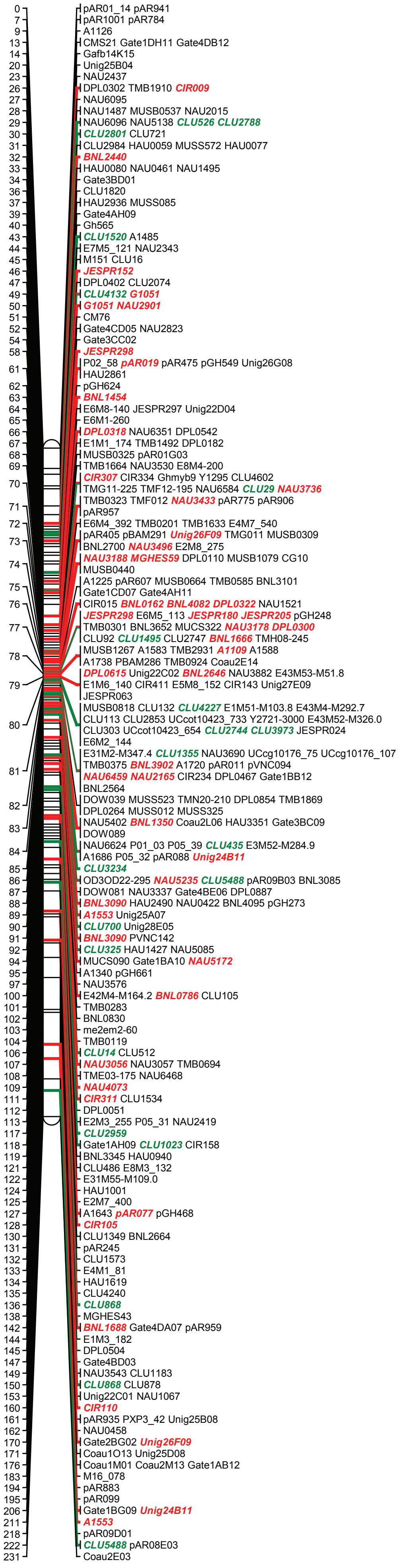
0 pAR01_14 pAR941
7 pAR1001 pAR784
9 A1126
13 CMS21 Gate1DH11 Gate4DB12
14 Gafb14K15
20 Unig25B04
23 NAU2437
26 DPL0302 TMB1910 CIR009
27 NAU6095
28 NAU1487 MUSB0537 NAU2015
29 NAU6096 NAU5138 CLU526 CLU2788
30 CLU2801 CLU721
31 CLU2984 HAU0059 MUSS572 HAU0077
32 BNL2440
33 HAU0080 NAU0461 NAU1495
34 Gate3BD01
36 CLU1820
37 HAU2936 MUSS085
39 Gate4AH09
40 Gh565
43 CLU1520 A1485
44 E7M5_121 NAU2343
45 M151 CLU16
46 JESPR152
47 DPL0402 CLU2074
49 CLU4132 G1051
50 G1051 NAU2901
51 CM76
52 Gate4CD05 NAU2823
54 Gate3CC02
58 JESPR298
61 P02_58 pAR019 pAR475 pGH549 Unig26G08
HAU2861
62 pGH624
63 BNL1454
64 E6M8-140 JESPR297 Unig22D04
65 E6M1-260
66 DPL0318 NAU6351 DPL0542
67 E1M1_174 TMB1492 DPL0182
68 MUSB0325 pAR01G03
69 TMB1664 NAU3530 E8M4-200
70 CIR307 CIR334 Ghmyb9 Y1295 CLU4602
TMG11-225 TMF12-195 NAU6584 CLU29 NAU3736
71 TMB0323 TMF012 NAU3433 pAR775 pAR906
pAR957
72 E6M4_392 TMB0201 TMB1633 E4M7_540
pAR405 pBAM291 Unig26F09 TMG011 MUSB0309
73 BNL2700 NAU3496 E2M8_275
NAU3188 MGHES59 DPL0110 MUSB1079 CG10
74 MUSB0440
A1225 pAR607 MUSB0664 TMB0585 BNL3101
75 Gate1CD07 Gate4AH11
76 CIR015 BNL0162 BNL4082 DPL0322 NAU1521
JESPR298 E6M5_113 JESPR180 JESPR205 pGH248
77 TMB0301 BNL3652 MUCS322 NAU3178 DPL0300
CLU92 CLU1495 CLU2747 BNL1666 TMH08-245
MUSB1267 A1583 TMB2931 A1109 A1588
78 A1738 PBAM286 TMB0924 Coau2E14
DPL0615 Unig22C02 BNL2646 NAU3882 E43M53-M51.8
79 E1M6_140 CIR411 E5M8_152 CIR143 Unig27E09
JESPR063
MUSB0818 CLU132 CLU4227 E1M51-M103.8 E43M4-M292.7
80 CLU113 CLU2853 UCcot10423_733 Y2721-3000 E43M52-M326.0
CLU303 UCcot10423_654 CLU2744 CLU3973 JESPR024
E6M2_144
E31M2-M347.4 CLU1355 NAU3690 UCcg10176_75 UCcg10176_107
81 TMB0375 BNL3902 A1720 pAR011 pVNC094
NAU6459 NAU2165 CIR234 DPL0467 Gate1BB12
BNL2564
82 DOW039 MUSS523 TMN20-210 DPL0854 TMB1869
DPL0264 MUSS012 MUSS325
83 NAU5402 BNL1350 Coau2L06 HAU3351 Gate3BC09
DOW089
84 NAU6624 P01_03 P05_39 CLU435 E3M52-M284.9
A1686 P05_32 pAR088 Unig24B11
85 CLU3234
86 OD3OD22-295 NAU5235 CLU5488 pAR09B03 BNL3085
87 DOW081 NAU3337 Gate4BE06 DPL0887
88 BNL3090 HAU2490 NAU0422 BNL4095 pGH273
89 A1553 Unig25A07
90 CLU700 Unig28E05
91 BNL3090 PVNC142
92 CLU325 HAU1427 NAU5085
94 MUCS090 Gate1BA10 NAU5172
95 A1340 pGH661
97 NAU3576
100 E42M4-M164.2 BNL0786 CLU105
101 TMB0283
102 BNL0830
103 me2em2-60
104 TMB0119
106 CLU14 CLU512
107 NAU3056 NAU3057 TMB0694
108 TME03-175 NAU6468
109 NAU4073
111 CIR311 CLU1534
112 DPL0051
113 E2M3_255 P05_31 NAU2419
117 CLU2959
118 Gate1AH09 CLU1023 CIR158
119 BNL3345 HAU0940
121 CLU486 E8M3_132
122 E31M55-M109.0
124 HAU1001
125 E2M7_400
127 A1643 pAR077 pGH468
128 CIR105
130 CLU1349 BNL2664
131 pAR245
132 CLU1573
133 E4M1_81
134 HAU1619
135 CLU4240
136 CLU868
138 MGHES43
142 BNL1688 Gate4DA07 pAR959
144 E1M3_182
145 DPL0504
147 Gate4BD03
149 NAU3543 CLU1183
150 CLU868 CLU878
153 Unig22C01 NAU1067
160 CIR110
161 pAR935 PXP3_42 Unig25B08
162 NAU0458
170 Gate2BG02 Unig26F09
171 Coau1O13 Unig25D08
176 Coau1M01 Coau2M13 Gate1AB12
183 M16_078
194 pAR883
195 pAR099
206 Gate1BG09 Unig24B11
211 A1553
218 pAR09D01
222 CLU5488 pAR08E03
231 Coau2E03

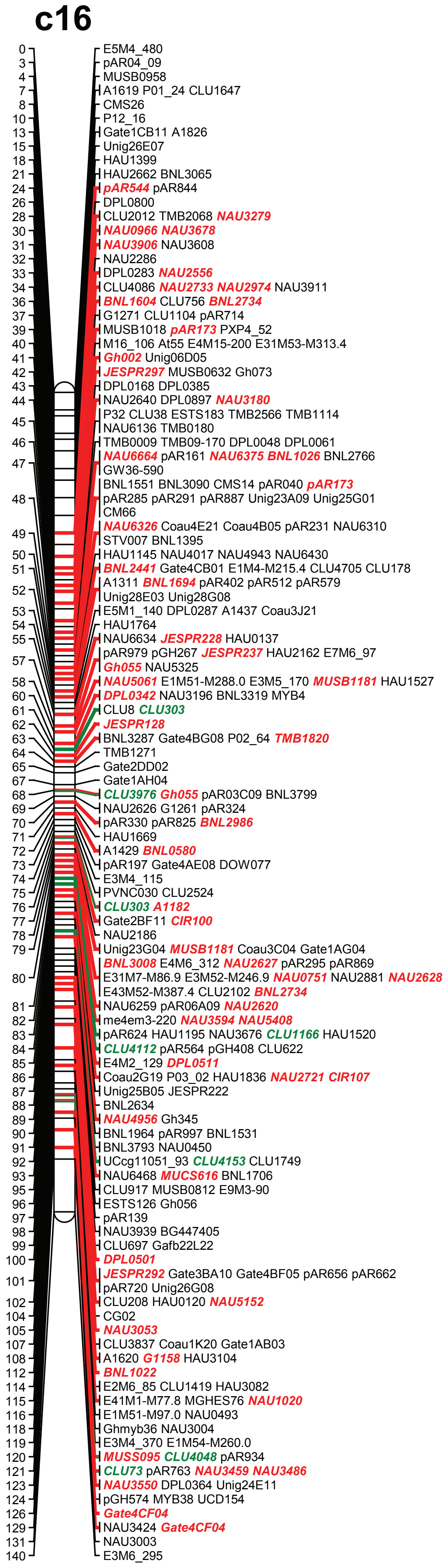
c16
0
3
4
7
8
10
13
15
18
21
24
26
28
30
31
32
33
34
36
37
39
40
41
42
43
44
45
46
47
48
49
50
51
52
53
54
55
57
58
60
61
62
63
64
65
67
68
69
70
71
72
73
74
75
76
77
78
79
80
81
82
83
84
85
86
87
88
89
90
91
92
93
95
96
97
98
99
100
101
102
104
105
107
108
112
114
115
116
118
119
120
121
123
124
126
129
131
140
E5M4_480
pAR04_09
MUSB0958
A1619 P01_24 CLU1647
CMS26
P12_16
Gate1CB11 A1826
Unig26E07
HAU1399
HAU2662 BNL3065
pAR544 pAR844
DPL0800
CLU2012 TMB2068 NAU3279
NAU0966 NAU3678
NAU3906 NAU3608
NAU2286
DPL0283 NAU2556
CLU4086 NAU2733 NAU2974 NAU3911
BNL1604 CLU756 BNL2734
G1271 CLU1104 pAR714
MUSB1018 pAR173 PXP4_52
M16_106 At55 E4M15-200 E31M53-M313.4
Gh002 Unig06D05
JESPR297 MUSB0632 Gh073
DPL0168 DPL0385
NAU2640 DPL0897 NAU3180
P32 CLU38 ESTS183 TMB2566 TMB1114
NAU6136 TMB0180
TMB0009 TMB09-170 DPL0048 DPL0061
NAU6664 pAR161 NAU6375 BNL1026 BNL2766
GW36-590
BNL1551 BNL3090 CMS14 pAR040 pAR173
pAR285 pAR291 pAR887 Unig23A09 Unig25G01
CM66
NAU6326 Coau4E21 Coau4B05 pAR231 NAU6310
STV007 BNL1395
HAU1145 NAU4017 NAU4943 NAU6430
BNL2441 Gate4CB01 E1M4-M215.4 CLU4705 CLU178
A1311 BNL1694 pAR402 pAR512 pAR579
Unig28E03 Unig28G08
E5M1_140 DPL0287 A1437 Coau3J21
HAU1764
NAU6634 JESPR228 HAU0137
pAR979 pGH267 JESPR237 HAU2162 E7M6_97
Gh055 NAU5325
NAU5061 E1M51-M288.0 E3M5_170 MUSB1181 HAU1527
DPL0342 NAU3196 BNL3319 MYB4
CLU8 CLU303
JESPR128
BNL3287 Gate4BG08 P02_64 TMB1820
TMB1271
Gate2DD02
Gate1AH04
CLU3976 Gh055 pAR03C09 BNL3799
NAU2626 G1261 pAR324
pAR330 pAR825 BNL2986
HAU1669
A1429 BNL0580
pAR197 Gate4AE08 DOW077
E3M4_115
PVNC030 CLU2524
CLU303 A1182
Gate2BF11 CIR100
NAU2186
Unig23G04 MUSB1181 Coau3C04 Gate1AG04
BNL3008 E4M6_312 NAU2627 pAR295 pAR869
E31M7-M86.9 E3M52-M246.9 NAU0751 NAU2881 NAU2628
E43M52-M387.4 CLU2102 BNL2734
NAU6259 pAR06A09 NAU2620
me4em3-220 NAU3594 NAU5408
pAR624 HAU1195 NAU3676 CLU1166 HAU1520
CLU4112 pAR564 pGH408 CLU622
E4M2_129 DPL0511
Coau2G19 P03_02 HAU1836 NAU2721 CIR107
Unig25B05 JESPR222
BNL2634
NAU4956 Gh345
BNL1964 pAR997 BNL1531
BNL3793 NAU0450
UCcg11051_93 CLU4153 CLU1749
NAU6468 MUCS616 BNL1706
CLU917 MUSB0812 E9M3-90
ESTS126 Gh056
pAR139
NAU3939 BG447405
CLU697 Gafb22L22
DPL0501
JESPR292 Gate3BA10 Gate4BF05 pAR656 pAR662
pAR720 Unig26G08
CLU208 HAU0120 NAU5152
CG02
NAU3053
CLU3837 Coau1K20 Gate1AB03
A1620 G1158 HAU3104
BNL1022
E2M6_85 CLU1419 HAU3082
E41M1-M77.8 MGHES76 NAU1020
E1M51-M97.0 NAU0493
Ghmyb36 NAU3004
E3M4_370 E1M54-M260.0
MUSS095 CLU4048 pAR934
CLU73 pAR763 NAU3459 NAU3486
NAU3550 DPL0364 Unig24E11
pGH574 MYB38 UCD154
Gate4CF04
NAU3424 Gate4CF04
NAU3003
E3M6_295

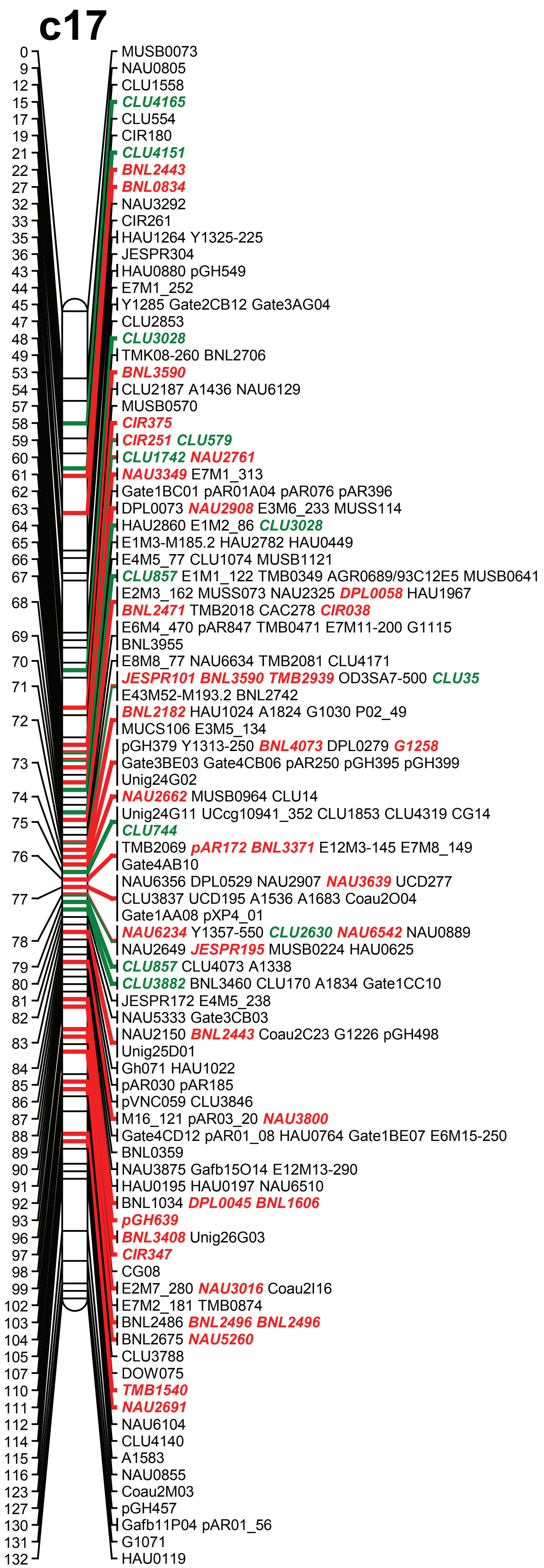
c17
0 MUSB0073
9 NAU0805
12 CLU1558
15 CLU4165
17 CLU554
19 CIR180
21 CLU4151
22 BNL2443
27 BNL0834
32 NAU3292
33 CIR261
35 HAU1264 Y1325-225
36 JESPR304
43 HAU0880 pGH549
44 E7M1_252
45 Y1285 Gate2CB12 Gate3AG04
47 CLU2853
48 CLU3028
49 TMK08-260 BNL2706
53 BNL3590
54 CLU2187 A1436 NAU6129
57 MUSB0570
58 CIR375
59 CIR251 CLU579
60 CLU1742 NAU2761
61 NAU3349 E7M1_313
62 Gate1BC01 pAR01A04 pAR076 pAR396
63 DPL0073 NAU2908 E3M6_233 MUSS114
64 HAU2860 E1M2_86 CLU3028
65 E1M3-M185.2 HAU2782 HAU0449
66 E4M5_77 CLU1074 MUSB1121
67 CLU857 E1M1_122 TMB0349 AGR0689/93C12E5 MUSB0641
68 E2M3_162 MUSS073 NAU2325 DPL0058 HAU1967
BNL2471 TMB2018 CAC278 CIR038
69 E6M4_470 pAR847 TMB0471 E7M11-200 G1115
BNL3955
70 E8M8_77 NAU6634 TMB2081 CLU4171
71 JESPR101 BNL3590 TMB2939 OD3SA7-500 CLU35
E43M52-M193.2 BNL2742
72 BNL2182 HAU1024 A1824 G1030 P02_49
MUCS106 E3M5_134
pGH379 Y1313-250 BNL4073 DPL0279 G1258
73 Gate3BE03 Gate4CB06 pAR250 pGH395 pGH399
Unig24G02
74 NAU2662 MUSB0964 CLU14
75 Unig24G11 UCcg10941_352 CLU1853 CLU4319 CG14
CLU744
76 TMB2069 pAR172 BNL3371 E12M3-145 E7M8_149
Gate4AB10
77 NAU6356 DPL0529 NAU2907 NAU3639 UCD277
CLU3837 UCD195 A1536 A1683 Coau2O04
Gate1AA08 pXP4_01
78 NAU6234 Y1357-550 CLU2630 NAU6542 NAU0889
NAU2649 JESPR195 MUSB0224 HAU0625
79 CLU857 CLU4073 A1338
80 CLU3882 BNL3460 CLU170 A1834 Gate1CC10
81 JESPR172 E4M5_238
82 NAU5333 Gate3CB03
83 NAU2150 BNL2443 Coau2C23 G1226 pGH498
Unig25D01
84 Gh071 HAU1022
85 pAR030 pAR185
86 pVNC059 CLU3846
87 M16_121 pAR03_20 NAU3800
88 Gate4CD12 pAR01_08 HAU0764 Gate1BE07 E6M15-250
89 BNL0359
90 NAU3875 Gafb15O14 E12M13-290
91 HAU0195 HAU0197 NAU6510
92 BNL1034 DPL0045 BNL1606
93 pGH639
96 BNL3408 Unig26G03
97 CIR347
98 CG08
99 E2M7_280 NAU3016 Coau2I16
102 E7M2_181 TMB0874
103 BNL2486 BNL2496 BNL2496
104 BNL2675 NAU5260
105 CLU3788
107 DOW075
110 TMB1540
111 NAU2691
112 NAU6104
114 CLU4140
115 A1583
116 NAU0855
123 Coau2M03
127 pGH457
130 Gafb11P04 pAR01_56
131 G1071
132 HAU0119

c18

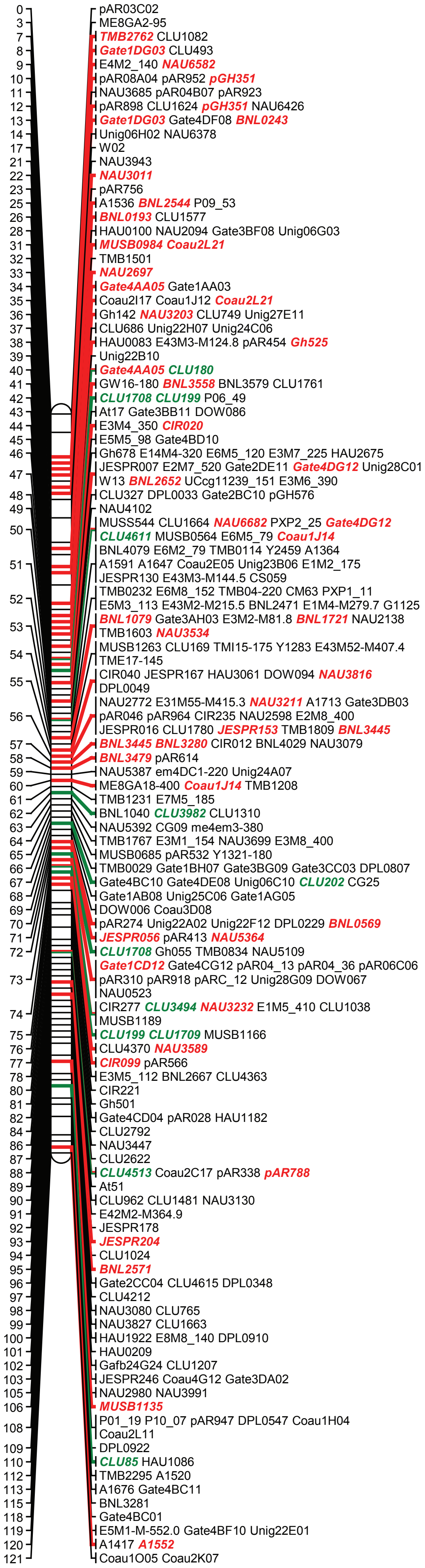
0
3
7
8
9
10
11
12
13
14
17
21
22
23
25
26
28
31
32
33
34
35
36
37
38
39
40
41
42
43
44
45
46
47
48
49
50
51
52
53
54
55
56
57
58
59
60
61
62
63
64
65
66
67
68
69
70
71
72
73
74
75
76
77
78
80
81
82
84
86
87
88
89
90
91
92
93
94
95
96
97
98
99
100
101
102
103
104
105
106
108
109
110
112
113
115
118
119
120
121
pAR03C02
ME8GA2-95
TMB2762 CLU1082
Gate1DG03 CLU493
E4M2_140 NAU6582
pAR08A04 pAR952 pGH351
NAU3685 pAR04B07 pAR923
pAR898 CLU1624 pGH351 NAU6426
Gate1DG03 Gate4DF08 BNL0243
Unig06H02 NAU6378
W02
NAU3943
NAU3011
pAR756
A1536 BNL2544 P09_53
BNL0193 CLU1577
HAU0100 NAU2094 Gate3BF08 Unig06G03
MUSB0984 Coau2L21
TMB1501
NAU2697
Gate4AA05 Gate1AA03
Coau2I17 Coau1J12 Coau2L21
Gh142 NAU3203 CLU749 Unig27E11
CLU686 Unig22H07 Unig24C06
HAU0083 E43M3-M124.8 pAR454 Gh525
Unig22B10
Gate4AA05 CLU180
GW16-180 BNL3558 BNL3579 CLU1761
CLU1708 CLU199 P06_49
At17 Gate3BB11 DOW086
E3M4_350 CIR020
E5M5_98 Gate4BD10
Gh678 E14M4-320 E6M5_120 E3M7_225 HAU2675
JESPR007 E2M7_520 Gate2DE11 Gate4DG12 Unig28C01
W13 BNL2652 UCcg11239_151 E3M6_390
CLU327 DPL0033 Gate2BC10 pGH576
NAU4102
MUSS544 CLU1664 NAU6682 PXP2_25 Gate4DG12
CLU4611 MUSB0564 E6M5_79 Coau1J14
BNL4079 E6M2_79 TMB0114 Y2459 A1364
A1591 A1647 Coau2E05 Unig23B06 E1M2_175
JESPR130 E43M3-M144.5 CS059
TMB0232 E6M8_152 TMB04-220 CM63 PXP1_11
E5M3_113 E43M2-M215.5 BNL2471 E1M4-M279.7 G1125
BNL1079 Gate3AH03 E3M2-M81.8 BNL1721 NAU2138
TMB1603 NAU3534
MUSB1263 CLU169 TMI15-175 Y1283 E43M52-M407.4
TME17-145
CIR040 JESPR167 HAU3061 DOW094 NAU3816
DPL0049
NAU2772 E31M55-M415.3 NAU3211 A1713 Gate3DB03
pAR046 pAR964 CIR235 NAU2598 E2M8_400
JESPR016 CLU1780 JESPR153 TMB1809 BNL3445
BNL3445 BNL3280 CIR012 BNL4029 NAU3079
BNL3479 pAR614
NAU5387 em4DC1-220 Unig24A07
ME8GA18-400 Coau1J14 TMB1208
TMB1231 E7M5_185
BNL1040 CLU3982 CLU1310
NAU5392 CG09 me4em3-380
TMB1767 E3M1_154 NAU3699 E3M8_400
MUSB0685 pAR532 Y1321-180
TMB0029 Gate1BH07 Gate3BG09 Gate3CC03 DPL0807
Gate4BC10 Gate4DE08 Unig06C10 CLU202 CG25
Gate1AB08 Unig25C06 Gate1AG05
DOW006 Coau3D08
pAR274 Unig22A02 Unig22F12 DPL0229 BNL0569
JESPR056 pAR413 NAU5364
CLU1708 Gh055 TMB0834 NAU5109
Gate1CD12 Gate4CG12 pAR04_13 pAR04_36 pAR06C06
pAR310 pAR918 pARC_12 Unig28G09 DOW067
NAU0523
CIR277 CLU3494 NAU3232 E1M5_410 CLU1038
MUSB1189
CLU199 CLU1709 MUSB1166
CLU4370 NAU3589
CIR099 pAR566
E3M5_112 BNL2667 CLU4363
CIR221
Gh501
Gate4CD04 pAR028 HAU1182
CLU2792
NAU3447
CLU2622
CLU4513 Coau2C17 pAR338 pAR788
At51
CLU962 CLU1481 NAU3130
E42M2-M364.9
JESPR178
JESPR204
CLU1024
BNL2571
Gate2CC04 CLU4615 DPL0348
CLU4212
NAU3080 CLU765
NAU3827 CLU1663
HAU1922 E8M8_140 DPL0910
HAU0209
Gafb24G24 CLU1207
JESPR246 Coau4G12 Gate3DA02
NAU2980 NAU3991
MUSB1135
P01_19 P10_07 pAR947 DPL0547 Coau1H04
Coau2L11
DPL0922
CLU85 HAU1086
TMB2295 A1520
A1676 Gate4BC11
BNL3281
Gate4BC01
E5M1-M-552.0 Gate4BF10 Unig22E01
A1417 A1552
Coau1O05 Coau2K07

c19

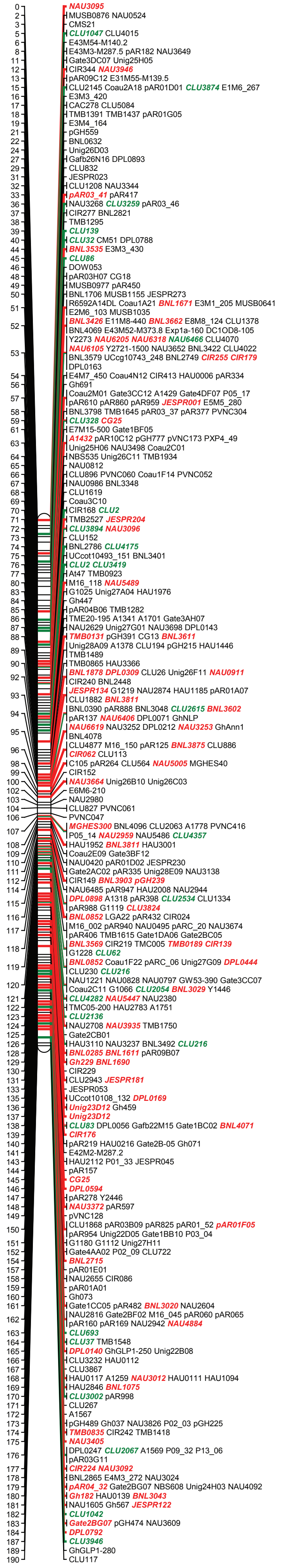
NAU3095
MUSB0876 NAU0524
CMS21
CLU1047 CLU4015
E43M54-M140.2
E43M3-M287.5 pAR182 NAU3649
Gate3DC07 Unig25H05
CIR344 NAU3946
pAR09C12 E31M55-M139.5
CLU2145 Coau2A18 pAR01D01 CLU3874 E1M6_267
E3M3_420
CAC278 CLU5084
TMB1391 TMB1437 pAR01G05
E3M4_164
pGH559
BNL0632
Unig26D03
Gafb26N16 DPL0893
CLU832
JESPR023
CLU1208 NAU3344
pAR03_41 pAR417
NAU3268 CLU3259 pAR03_46
CIR277 BNL2821
TMB1295
CLU139
CLU32 CM51 DPL0788
BNL3535 E3M3_430
CLU86
DOW053
pAR03H07 CG18
MUSB0977 pAR450
BNL1706 MUSB1155 JESPR273
R6592A14DL Coau1A21 BNL1671 E3M1_205 MUSB0641
E2M6_103 MUSB1035
BNL3426 E11M8-440 BNL3662 E8M8_124 CLU1378
BNL4069 E43M52-M373.8 Exp1a-160 DC1OD8-105
Y2273 NAU6205 NAU6318 NAU6466 CLU4070
NAU6105 Y2721-1500 NAU3652 BNL3422 CLU4022
BNL3579 UCcg10743_248 BNL2749 CIR255 CIR179
DPL0163
E4M7_450 Coau4N12 CIR413 HAU0006 pAR334
Gh691
Coau2M01 Gate3CC12 A1429 Gate4DF07 P05_17
pAR610 pAR860 pAR959 JESPR001 E5M5_280
BNL3798 TMB1645 pAR03_37 pAR377 PVNC304
CLU328 CG25
E7M15-500 Gate1BF05
A1432 pAR10C12 pGH777 pVNC173 PXP4_49
Unig25H06 NAU3498 Coau2C01
NBS535 Unig26C11 TMB1934
NAU0812
CLU896 PVNC060 Coau1F14 PVNC052
NAU0986 BNL3348
CLU1619
Coau3C10
CIR168 CLU2
TMB2527 JESPR204
CLU3894 NAU3096
CLU152
BNL2786 CLU4175
UCcot10493_151 BNL3401
CLU2 CLU3419
At47 TMB0923
M16_118 NAU5489
G1025 Unig27A04 HAU1976
Gh447
pAR04B06 TMB1282
TME20-195 A1341 A1701 Gate3AH07
NAU2629 Unig27G01 NAU3698 DPL0143
TMB0131 pGH391 CG13 BNL3611
Unig28A09 A1378 CLU194 pGH215 HAU1446
TMB1489
TMB0865 HAU3366
BNL1878 DPL0309 CLU26 Unig26F11 NAU0911
CIR240 BNL2448
JESPR134 G1219 NAU2874 HAU1185 pAR01A07
CLU1882 BNL3811
BNL0390 pAR888 BNL3048 CLU2615 BNL3602
pAR137 NAU6406 DPL0071 GhNLP
NAU6619 NAU3252 DPL0212 NAU3253 GhAnn1
BNL4078
CLU4877 M16_150 pAR125 BNL3875 CLU886
CIR062 CLU113
C105 pAR264 CLU564 NAU5005 MGHES40
CIR152
NAU3664 Unig26B10 Unig26C03
E6M6-210
NAU2980
CLU827 PVNC061
PVNC047
MGHES300 BNL4096 CLU2063 A1778 PVNC416
P05_14 NAU2959 NAU5486 CLU4357
HAU1952 BNL3811 HAU3001
Coau2E09 Gate3BF12
NAU0420 pAR01D02 JESPR230
Gate2AC02 pAR335 Unig28E09 NAU3138
CIR149 BNL3903 pGH239
NAU6485 pAR947 HAU2008 NAU2944
DPL0898 A1318 pAR398 CLU2534 CLU1334
pAR988 G1119 CLU3824
BNL0852 LGA22 pAR432 CIR024
M16_002 pAR940 NAU0495 pARC_20 NAU3674
pAR406 TMB1615 Gate1DA06 Gate2BC05
BNL3569 CIR219 TMC005 TMB0189 CIR139
G1228 CLU62
BNL0852 Coau1F22 pARC_06 Unig27G09 DPL0444
CLU230 CLU216
NAU1221 NAU0828 NAU0797 GW53-390 Gate3CC07
Coau2C11 G1066 CLU2054 BNL3029 Y1446
CLU4282 NAU5447 NAU2380
TMC05-200 HAU2783 A1751
CLU2136
NAU2708 NAU3935 TMB1750
Gate2CB01
HAU3110 NAU3237 BNL3492 CLU216
BNL0285 BNL1611 pAR09B07
Gh229 BNL1690
CIR229
CLU2943 JESPR181
JESPR053
UCcot10108_132 DPL0169
Unig23D12 Gh459
Unig23D12
CLU83 DPL0056 Gafb22M15 Gate1BC02 BNL4071
CIR176
pAR219 HAU0216 Gate2B-05 Gh071
E42M2-M287.2
HAU2112 P01_33 JESPR045
pAR157
CG25
DPL0594
pAR278 Y2446
NAU3372 pAR597
pVNC128
CLU1868 pAR03B09 pAR825 pAR01_52 pAR01F05
pAR954 Unig22D05 Gate1BB10 P03_04
G1180 G1112 Unig27H11
Gate4AA02 P02_09 CLU722
BNL2715
pAR01E01
NAU2655 CIR086
pAR01A01
Gh073
Gate1CC05 pAR482 BNL3020 NAU2604
NAU2816 Gate2BF02 M16_045 pAR060 pAR065
pAR160 pAR169 NAU2942 NAU4884
CLU693
CLU37 TMB1548
DPL0140 GhGLP1-250 Unig22B08
CLU3232 HAU0112
CLU3867
HAU0117 A1259 NAU3012 HAU0111 HAU1094
HAU2846 BNL1075
CLU3002 pAR998
CLU267
A1567
pGH489 Gh037 NAU3826 P02_03 pGH225
TMB0835 CIR242 TMB1418
NAU3405
DPL0247 CLU2067 A1569 P09_32 P13_06
pAR03G11
CIR224 NAU3092
BNL2865 E4M3_272 NAU3024
pAR04_32 Gate2BG07 NBS608 Unig24H03 NAU4092
Gh182 HAU0139 BNL3043
NAU1605 Gh567 JESPR122
CLU1042
Gate2BG07 pGH474 NAU3609
DPL0792
CLU3946
GhGLP1-280
CLU117

c20

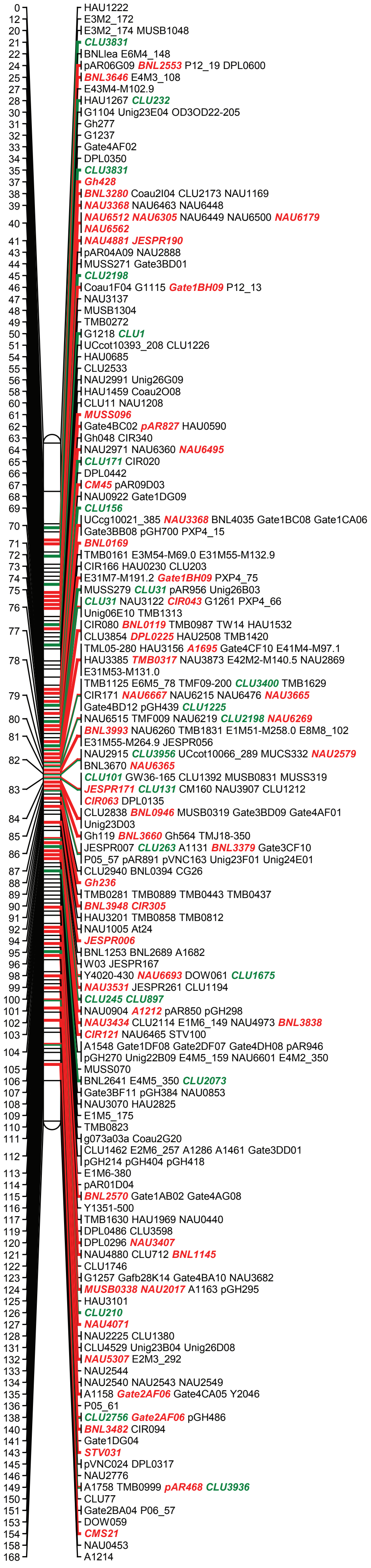
0
12
20
21
22
24
25
27
28
30
31
32
33
34
35
37
38
39
40
41
43
44
45
46
47
48
49
50
51
54
55
56
58
60
61
62
63
64
65
66
67
68
69
70
71
72
73
74
75
76
77
78
79
80
81
82
83
84
85
86
87
88
89
90
91
92
94
95
96
98
99
100
101
102
103
104
105
106
107
108
109
110
111
112
113
114
115
116
117
119
120
121
122
123
124
125
126
127
128
131
132
133
134
135
136
138
140
141
143
145
146
149
150
151
153
154
158
168
HAU1222
E3M2_172
E3M2_174 MUSB1048
CLU3831
BNLlea E6M4_148
pAR06G09 BNL2553 P12_19 DPL0600
BNL3646 E4M3_108
E43M4-M102.9
HAU1267 CLU232
G1104 Unig23E04 OD3OD22-205
Gh277
G1237
Gate4AF02
DPL0350
CLU3831
Gh428
BNL3280 Coau2I04 CLU2173 NAU1169
NAU3368 NAU6463 NAU6448
NAU6512 NAU6305 NAU6449 NAU6500 NAU6179
NAU6562
NAU4881 JESPR190
pAR04A09 NAU2888
MUSS271 Gate3BD01
CLU2198
Coau1F04 G1115 Gate1BH09 P12_13
NAU3137
MUSB1304
TMB0272
G1218 CLU1
UCcot10393_208 CLU1226
HAU0685
CLU2533
NAU2991 Unig26G09
HAU1459 Coau2O08
CLU11 NAU1208
MUSS096
Gate4BC02 pAR827 HAU0590
Gh048 CIR340
NAU2971 NAU6360 NAU6495
CLU171 CIR020
DPL0442
CM45 pAR09D03
NAU0922 Gate1DG09
CLU156
UCcg10021_385 NAU3368 BNL4035 Gate1BC08 Gate1CA06
Gate3BB08 pGH700 PXP4_15
BNL0169
TMB0161 E3M54-M69.0 E31M55-M132.9
CIR166 HAU0230 CLU203
E31M7-M191.2 Gate1BH09 PXP4_75
MUSS279 CLU31 pAR956 Unig26B03
CLU31 NAU3122 CIR043 G1261 PXP4_66
Unig06E10 TMB1313
CIR080 BNL0119 TMB0987 TW14 HAU1532
CLU3854 DPL0225 HAU2508 TMB1420
TML05-280 HAU3156 A1695 Gate4CF10 E41M4-M97.1
HAU3385 TMB0317 NAU3873 E42M2-M140.5 NAU2869
E31M53-M131.0
TMB1125 E6M5_78 TMF09-200 CLU3400 TMB1629
CIR171 NAU6667 NAU6215 NAU6476 NAU3665
Gate4BD12 pGH439 CLU1225
NAU6515 TMF009 NAU6219 CLU2198 NAU6269
BNL3993 NAU6260 TMB1831 E1M51-M258.0 E8M8_102
E31M55-M264.9 JESPR056
NAU2915 CLU3956 UCcot10066_289 MUCS332 NAU2579
BNL3670 NAU6365
CLU101 GW36-165 CLU1392 MUSB0831 MUSS319
JESPR171 CLU131 CM160 NAU3907 CLU1212
CIR063 DPL0135
CLU2838 BNL0946 MUSB0319 Gate3BD09 Gate4AF01
Unig23D03
Gh119 BNL3660 Gh564 TMJ18-350
JESPR007 CLU263 A1131 BNL3379 Gate3CF10
P05_57 pAR891 pVNC163 Unig23F01 Unig24E01
CLU2940 BNL0394 CG26
Gh236
TMB0281 TMB0889 TMB0443 TMB0437
BNL3948 CIR305
HAU3201 TMB0858 TMB0812
NAU1005 At24
JESPR006
BNL1253 BNL2689 A1682
W03 JESPR167
Y4020-430 NAU6693 DOW061 CLU1675
NAU3531 JESPR261 CLU1194
CLU245 CLU897
NAU0904 A1212 pAR850 pGH298
NAU3434 CLU2114 E1M6_149 NAU4973 BNL3838
CIR121 NAU6465 STV100
A1548 Gate1DF08 Gate2DF07 Gate4DH08 pAR946
pGH270 Unig22B09 E4M5_159 NAU6601 E4M2_350
MUSS070
BNL2641 E4M5_350 CLU2073
Gate3BF11 pGH384 NAU0853
NAU3070 HAU2825
E1M5_175
TMB0823
g073a03a Coau2G20
CLU1462 E2M6_257 A1286 A1461 Gate3DD01
pGH214 pGH404 pGH418
E1M6-380
pAR01D04
BNL2570 Gate1AB02 Gate4AG08
Y1351-500
TMB1630 HAU1969 NAU0440
DPL0486 CLU3598
DPL0296 NAU3407
NAU4880 CLU712 BNL1145
CLU1746
G1257 Gafb28K14 Gate4BA10 NAU3682
MUSB0338 NAU2017 A1163 pGH295
HAU3101
CLU210
NAU4071
NAU2225 CLU1380
CLU4529 Unig23B04 Unig26D08
NAU5307 E2M3_292
NAU2544
NAU2540 NAU2543 NAU2549
A1158 Gate2AF06 Gate4CA05 Y2046
P05_61
CLU2756 Gate2AF06 pGH486
BNL3482 CIR094
Gate1DG04
STV031
pVNC024 DPL0317
NAU2776
A1758 TMB0999 pAR468 CLU3936
CLU77
Gate2BA04 P06_57
DOW059
CMS21
NAU0453
A1214

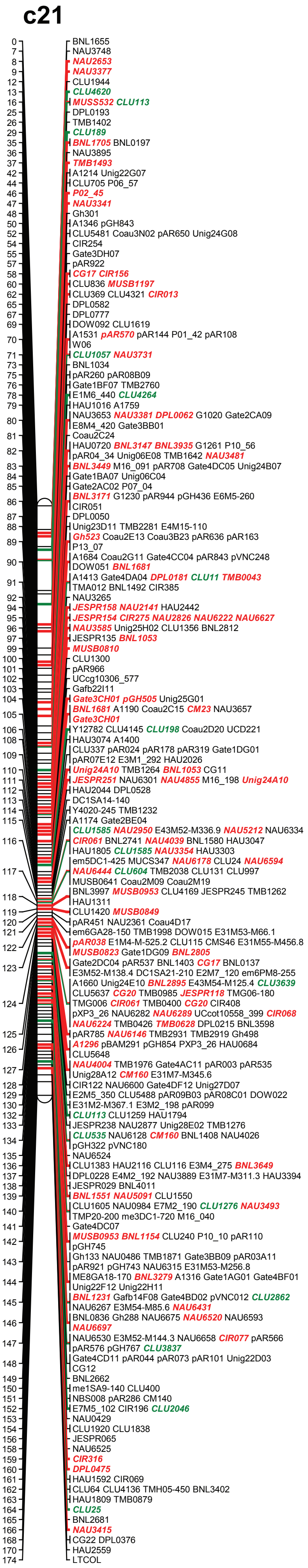
c21
0 7 8 9 12 13 16 25 26 29 35 36 37 42 44 46 47 48 50 52 54 55 57 58 60 62 65 67 69 70 71 73 75 76 78 79 80 81 82 83 84 85 86 87 88 89 90 91 92 94 95 96 97 99 100 101 102 103 104 105 106 108 109 110 111 112 113 114 115 116 117 118 119 120 121 122 123 124 125 126 127 128 129 130 132 133 134 135 136 137 138 139 140 141 142 143 144 145 146 147 148 149 150 151 152 153 154 156 158 159 160 161 162 163 164 165 166 168 170 174
BNL1655
NAU3748
NAU2653
NAU3377
CLU1944
CLU4620
MUSS532 CLU113
DPL0193
TMB1402
CLU189
BNL1705 BNL0197
NAU3895
TMB1493
A1214 Unig22G07
CLU705 P06_57
P02_45
NAU3341
Gh301
A1346 pGH843
CLU5481 Coau3N02 pAR650 Unig24G08
CIR254
Gate3DH07
pAR922
CG17 CIR156
CLU836 MUSB1197
CLU369 CLU4321 CIR013
DPL0582
DPL0777
DOW092 CLU1619
A1531 pAR570 pAR144 P01_42 pAR108
W06
CLU1057 NAU3731
BNL1034
pAR260 pAR08B09
Gate1BF07 TMB2760
E1M6_440 CLU4264
HAU1016 A1759
NAU3653 NAU3381 DPL0062 G1020 Gate2CA09
E8M4_420 Gate3BB01
Coau2C24
HAU0720 BNL3147 BNL3935 G1261 P10_56
pAR04_34 Unig06E08 TMB1642 NAU3481
BNL3449 M16_091 pAR708 Gate4DC05 Unig24B07
Gate1BA07 Unig06C04
Gate2AC02 P07_04
BNL3171 G1230 pAR944 pGH436 E6M5-260
CIR051
DPL0050
Unig23D11 TMB2281 E4M15-110
Gh523 Coau2E13 Coau3B23 pAR636 pAR163
P13_07
A1684 Coau2G11 Gate4CC04 pAR843 pVNC248
DOW051 BNL1681
A1413 Gate4DA04 DPL0181 CLU11 TMB0043
TMA012 BNL1492 CIR385
NAU3265
JESPR158 NAU2141 HAU2442
JESPR154 CIR275 NAU2826 NAU6222 NAU6627
NAU3585 Unig25H02 CLU1356 BNL2812
JESPR135 BNL1053
MUSB0810
CLU1300
pAR966
UCcg10306_577
Gafb22I11
Gate3CH01 pGH505 Unig25G01
BNL1681 A1190 Coau2C15 CM23 NAU3657
Gate3CH01
Y12782 CLU4145 CLU198 Coau2D20 UCD221
HAU3074 A1400
CLU337 pAR024 pAR178 pAR319 Gate1DG01
pAR07E12 E3M1_292 HAU2026
Unig24A10 TMB1264 BNL1053 CG11
JESPR251 NAU6301 NAU4855 M16_198 Unig24A10
HAU2044 DPL0528
DC1SA14-140
Y4020-245 TMB1232
A1174 Gate2BE04
CLU1585 NAU2950 E43M52-M336.9 NAU5212 NAU6334
CIR061 BNL2741 NAU4039 BNL1580 HAU3047
HAU1805 CLU1585 NAU3354 HAU3303
em5DC1-425 MUCS347 NAU6178 CLU24 NAU6594
NAU6444 CLU604 TMB2038 CLU131 CLU997
MUSB0641 Coau2M09 Coau2M19
BNL3997 MUSB0953 CLU4169 JESPR245 TMB1262
HAU1311
CLU1420 MUSB0849
pAR451 NAU2361 Coau4D17
em6GA28-150 TMB1998 DOW015 E31M53-M66.1
pAR038 E1M4-M-525.2 CLU115 CMS46 E31M55-M456.8
MUSB0823 Gate1DG09 BNL2805
Gate2DC04 pAR537 BNL1403 CG17 BNL0137
E3M52-M138.4 DC1SA21-210 E2M7_120 em6PM8-255
A1660 Unig24E10 BNL2895 E43M54-M125.4 CLU3639
CLU5637 CG20 TMB0985 JESPR118 TMG06-180
TMG006 CIR061 TMB0400 CG20 CIR408
pXP3_26 NAU6282 NAU6289 UCcot10558_399 CIR068
NAU6224 TMB0426 TMB0628 DPL0215 BNL3598
pAR785 NAU6146 TMB2931 TMB2919 Gh498
A1296 pBAM291 pGH854 PXP3_26 HAU0684
CLU5648
NAU4004 TMB1976 Gate4AC11 pAR003 pAR535
Unig28A12 CM160 E31M7-M345.6
CIR122 NAU6600 Gate4DF12 Unig27D07
E2M5_350 CLU5488 pAR09B03 pAR08C01 DOW022
E31M2-M367.1 E3M2_198 pAR099
CLU113 CLU1259 HAU1794
JESPR238 NAU2877 Unig28E02 TMB1276
CLU535 NAU6128 CM160 BNL1408 NAU4026
pGH322 pVNC180
NAU6524
CLU1383 HAU2116 CLU116 E3M4_275 BNL3649
DPL0228 E4M2_192 NAU3889 E31M7-M311.3 HAU3394
JESPR029 BNL4011
BNL1551 NAU5091 CLU1550
CLU1605 NAU0984 E7M2_190 CLU1276 NAU3493
TMP20-200 me3DC1-720 M16_040
Gate4DC07
MUSB0953 BNL1154 CLU240 P10_10 pAR110
pGH745
Gh133 NAU0486 TMB1871 Gate3BB09 pAR03A11
pAR921 pGH743 NAU6315 E31M53-M256.8
ME8GA18-170 BNL3279 A1316 Gate1AG01 Gate4BF01
Unig22F12 Unig22H11
BNL1231 Gafb14F08 Gate4BD02 pVNC012 CLU2862
NAU6267 E3M54-M85.6 NAU6431
BNL0836 Gh288 NAU6675 NAU6520 NAU6593
NAU6697
NAU6530 E3M52-M144.3 NAU6658 CIR077 pAR566
pAR576 pGH767 CLU3837
Gate4CD11 pAR044 pAR073 pAR101 Unig22D03
CG12
BNL2662
me1SA9-140 CLU400
NBS008 pAR286 CM140
E7M5_102 CIR196 CLU2046
NAU0429
CLU1920 CLU1838
JESPR065
NAU6525
CIR316
DPL0475
HAU1592 CIR069
CLU64 CLU4136 TMH05-450 BNL3402
HAU1809 TMB0879
CLU25
BNL2681
NAU3415
CG22 DPL0376
HAU2559
LTCOL

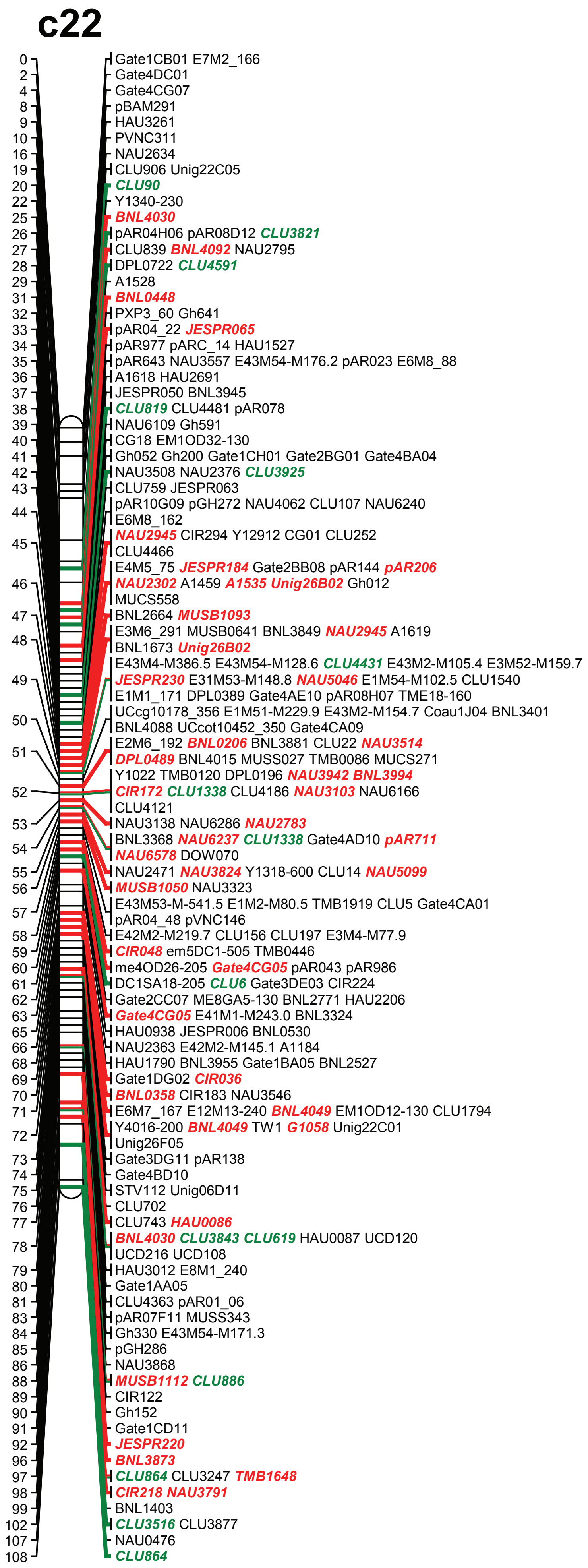
c22
0
2
4
8
9
10
16
19
20
22
25
26
27
28
29
31
32
33
34
35
36
37
38
39
40
41
42
43
44
45
46
47
48
49
50
51
52
53
54
55
56
57
58
59
60
61
62
63
65
66
68
69
70
71
72
73
74
75
76
77
78
79
80
81
83
84
85
86
88
89
90
91
92
96
97
98
99
102
107
108
Gate1CB01 E7M2_166
Gate4DC01
Gate4CG07
pBAM291
HAU3261
PVNC311
NAU2634
CLU906 Unig22C05
CLU90
Y1340-230
BNL4030
pAR04H06 pAR08D12 CLU3821
CLU839 BNL4092 NAU2795
DPL0722 CLU4591
A1528
BNL0448
PXP3_60 Gh641
pAR04_22 JESPR065
pAR977 pARC_14 HAU1527
pAR643 NAU3557 E43M54-M176.2 pAR023 E6M8_88
A1618 HAU2691
JESPR050 BNL3945
CLU819 CLU4481 pAR078
NAU6109 Gh591
CG18 EM1OD32-130
Gh052 Gh200 Gate1CH01 Gate2BG01 Gate4BA04
NAU3508 NAU2376 CLU3925
CLU759 JESPR063
pAR10G09 pGH272 NAU4062 CLU107 NAU6240
E6M8_162
NAU2945 CIR294 Y12912 CG01 CLU252
CLU4466
E4M5_75 JESPR184 Gate2BB08 pAR144 pAR206
NAU2302 A1459 A1535 Unig26B02 Gh012
MUCS558
BNL2664 MUSB1093
E3M6_291 MUSB0641 BNL3849 NAU2945 A1619
BNL1673 Unig26B02
E43M4-M386.5 E43M54-M128.6 CLU4431 E43M2-M105.4 E3M52-M159.7
JESPR230 E31M53-M148.8 NAU5046 E1M54-M102.5 CLU1540
E1M1_171 DPL0389 Gate4AE10 pAR08H07 TME18-160
UCcg10178_356 E1M51-M229.9 E43M2-M154.7 Coau1J04 BNL3401
BNL4088 UCcot10452_350 Gate4CA09
E2M6_192 BNL0206 BNL3881 CLU22 NAU3514
DPL0489 BNL4015 MUSS027 TMB0086 MUCS271
Y1022 TMB0120 DPL0196 NAU3942 BNL3994
CIR172 CLU1338 CLU4186 NAU3103 NAU6166
CLU4121
NAU3138 NAU6286 NAU2783
BNL3368 NAU6237 CLU1338 Gate4AD10 pAR711
NAU6578 DOW070
NAU2471 NAU3824 Y1318-600 CLU14 NAU5099
MUSB1050 NAU3323
E43M53-M-541.5 E1M2-M80.5 TMB1919 CLU5 Gate4CA01
pAR04_48 pVNC146
E42M2-M219.7 CLU156 CLU197 E3M4-M77.9
CIR048 em5DC1-505 TMB0446
me4OD26-205 Gate4CG05 pAR043 pAR986
DC1SA18-205 CLU6 Gate3DE03 CIR224
Gate2CC07 ME8GA5-130 BNL2771 HAU2206
Gate4CG05 E41M1-M243.0 BNL3324
HAU0938 JESPR006 BNL0530
NAU2363 E42M2-M145.1 A1184
HAU1790 BNL3955 Gate1BA05 BNL2527
Gate1DG02 CIR036
BNL0358 CIR183 NAU3546
E6M7_167 E12M13-240 BNL4049 EM1OD12-130 CLU1794
Y4016-200 BNL4049 TW1 G1058 Unig22C01
Unig26F05
Gate3DG11 pAR138
Gate4BD10
STV112 Unig06D11
CLU702
CLU743 HAU0086
BNL4030 CLU3843 CLU619 HAU0087 UCD120
UCD216 UCD108
HAU3012 E8M1_240
Gate1AA05
CLU4363 pAR01_06
pAR07F11 MUSS343
Gh330 E43M54-M171.3
pGH286
NAU3868
MUSB1112 CLU886
CIR122
Gh152
Gate1CD11
JESPR220
BNL3873
CLU864 CLU3247 TMB1648
CIR218 NAU3791
BNL1403
CLU3516 CLU3877
NAU0476
CLU864

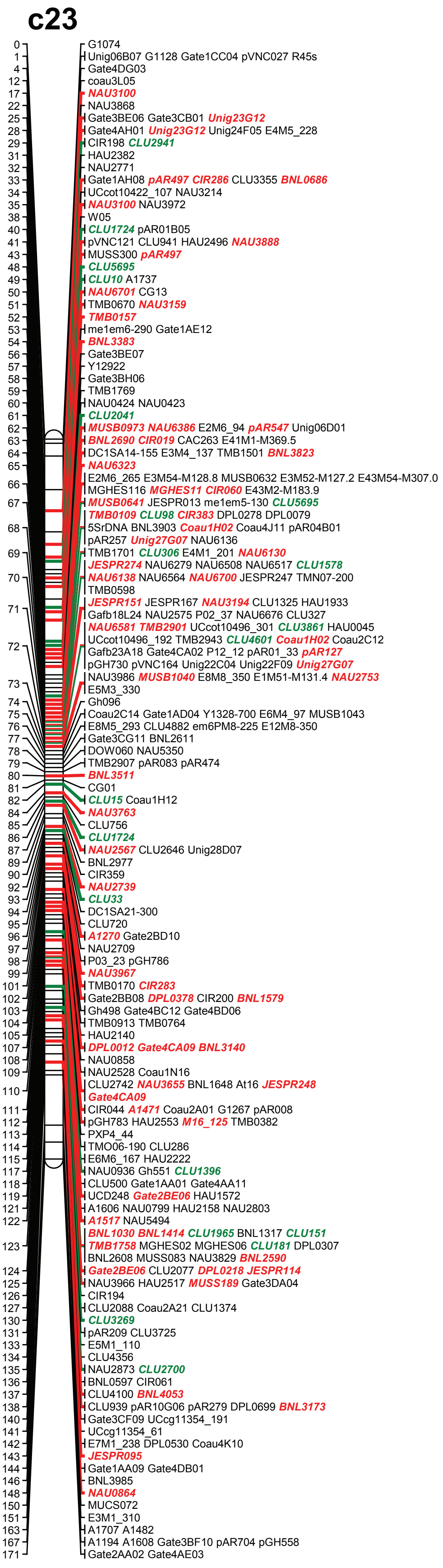
c23
0 1 4 12 17 22 25 28 29 31 32 33 34 35 38 40 41 43 48 49 50 51 52 53 54 56 57 58 59 60 61 62 63 64 65 66 67 68 69 70 71 72 73 74 75 76 77 78 79 80 81 82 84 85 86 87 89 90 92 93 94 95 96 97 98 99 101 102 103 104 105 107 108 109 110 111 112 113 114 115 117 118 119 121 122 123 124 125 126 127 130 131 133 134 135 136 137 138 140 141 142 143 144 146 148 150 151 163 167 171
G1074
Unig06B07 G1128 Gate1CC04 pVNC027 R45s
Gate4DG03
coau3L05
NAU3100
NAU3868
Gate3BE06 Gate3CB01 Unig23G12
Gate4AH01 Unig23G12 Unig24F05 E4M5_228
CIR198 CLU2941
HAU2382
NAU2771
Gate1AH08 pAR497 CIR286 CLU3355 BNL0686
UCcot10422_107 NAU3214
NAU3100 NAU3972
W05
CLU1724 pAR01B05
pVNC121 CLU941 HAU2496 NAU3888
MUSS300 pAR497
CLU5695
CLU10 A1737
NAU6701 CG13
TMB0670 NAU3159
TMB0157
me1em6-290 Gate1AE12
BNL3383
Gate3BE07
Y12922
Gate3BH06
TMB1769
NAU0424 NAU0423
CLU2041
MUSB0973 NAU6386 E2M6_94 pAR547 Unig06D01
BNL2690 CIR019 CAC263 E41M1-M369.5
DC1SA14-155 E3M4_137 TMB1501 BNL3823
NAU6323
E2M6_265 E3M54-M128.8 MUSB0632 E3M52-M127.2 E43M54-M307.0
MGHES116 MGHES11 CIR060 E43M2-M183.9
MUSB0641 JESPR013 me1em5-130 CLU5695
TMB0109 CLU98 CIR383 DPL0278 DPL0079
5SrDNA BNL3903 Coau1H02 Coau4J11 pAR04B01
pAR257 Unig27G07 NAU6136
TMB1701 CLU306 E4M1_201 NAU6130
JESPR274 NAU6279 NAU6508 NAU6517 CLU1578
NAU6138 NAU6564 NAU6700 JESPR247 TMN07-200
TMB0598
JESPR151 JESPR167 NAU3194 CLU1325 HAU1933
Gafb18L24 NAU2575 P02_37 NAU6676 CLU327
NAU6581 TMB2901 UCcot10496_301 CLU3861 HAU0045
UCcot10496_192 TMB2943 CLU4601 Coau1H02 Coau2C12
Gafb23A18 Gate4CA02 P12_12 pAR01_33 pAR127
pGH730 pVNC164 Unig22C04 Unig22F09 Unig27G07
NAU3986 MUSB1040 E8M8_350 E1M51-M131.4 NAU2753
E5M3_330
Gh096
Coau2C14 Gate1AD04 Y1328-700 E6M4_97 MUSB1043
E8M5_293 CLU4882 em6PM8-225 E12M8-350
Gate3CG11 BNL2611
DOW060 NAU5350
TMB2907 pAR083 pAR474
BNL3511
CG01
CLU15 Coau1H12
NAU3763
CLU756
CLU1724
NAU2567 CLU2646 Unig28D07
BNL2977
CIR359
NAU2739
CLU33
DC1SA21-300
CLU720
A1270 Gate2BD10
NAU2709
P03_23 pGH786
NAU3967
TMB0170 CIR283
Gate2BB08 DPL0378 CIR200 BNL1579
Gh498 Gate4BC12 Gate4BD06
TMB0913 TMB0764
HAU2140
DPL0012 Gate4CA09 BNL3140
NAU0858
NAU2528 Coau1N16
CLU2742 NAU3655 BNL1648 At16 JESPR248
Gate4CA09
CIR044 A1471 Coau2A01 G1267 pAR008
pGH783 HAU2553 M16_125 TMB0382
PXP4_44
TMO06-190 CLU286
E6M6_167 HAU2222
NAU0936 Gh551 CLU1396
CLU500 Gate1AA01 Gate4AA11
UCD248 Gate2BE06 HAU1572
A1606 NAU0799 HAU2158 NAU2803
A1517 NAU5494
BNL1030 BNL1414 CLU1965 BNL1317 CLU151
TMB1758 MGHES02 MGHES06 CLU181 DPL0307
BNL2608 MUSS083 NAU3829 BNL2590
Gate2BE06 CLU2077 DPL0218 JESPR114
NAU3966 HAU2517 MUSS189 Gate3DA04
CIR194
CLU2088 Coau2A21 CLU1374
CLU3269
pAR209 CLU3725
E5M1_110
CLU4356
NAU2873 CLU2700
BNL0597 CIR061
CLU4100 BNL4053
CLU939 pAR10G06 pAR279 DPL0699 BNL3173
Gate3CF09 UCcg11354_191
UCcg11354_61
E7M1_238 DPL0530 Coau4K10
JESPR095
Gate1AA09 Gate4DB01
BNL3985
NAU0864
MUCS072
E3M1_310
A1707 A1482
A1194 A1608 Gate3BF10 pAR704 pGH558
Gate2AA02 Gate4AE03

c24

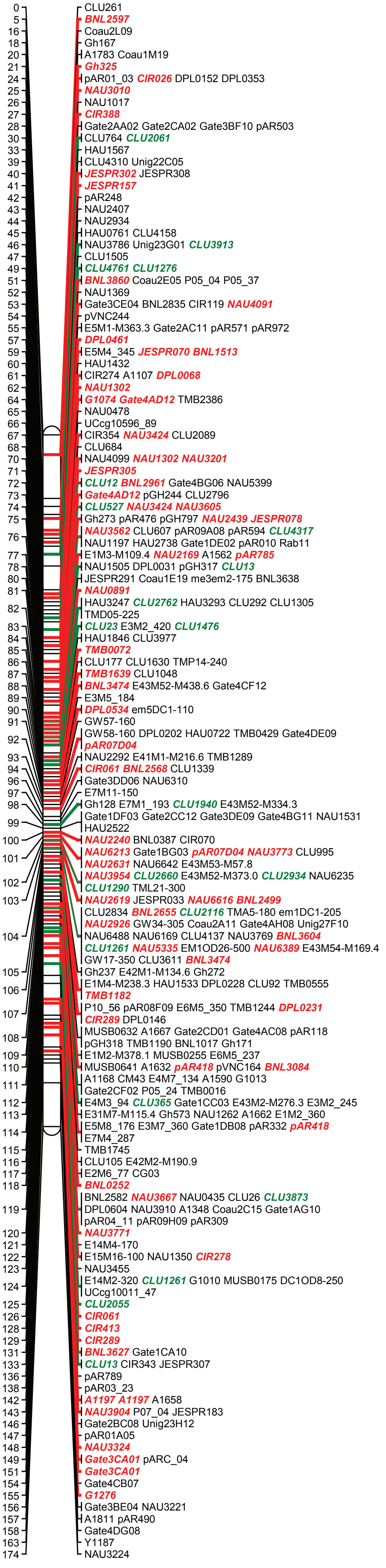
0 5 16 18 20 21 24 25 26 27 28 30 33 39 40 41 42 43 44 45 46 47 49 51 52 53 54 55 57 59 60 61 62 64 65 66 67 68 70 71 72 73 74 75 76 77 78 80 81 82 83 84 85 86 87 88 89 90 91 92 93 94 96 97 98 99 100 101 102 103 104 105 106 107 108 109 110 111 112 113 114 115 116 117 118 119 120 121 122 123 124 125 126 128 129 131 133 136 138 142 143 146 147 148 149 151 154 155 156 157 158 163 174
CLU261
BNL2597
Coau2L09
Gh167
A1783 Coau1M19
Gh325
pAR01_03 CIR026 DPL0152 DPL0353
NAU3010
NAU1017
CIR388
Gate2AA02 Gate2CA02 Gate3BF10 pAR503
CLU764 CLU2061
HAU1567
CLU4310 Unig22C05
JESPR302 JESPR308
JESPR157
pAR248
NAU2407
NAU2934
HAU0761 CLU4158
NAU3786 Unig23G01 CLU3913
CLU1505
CLU4761 CLU1276
BNL3860 Coau2E05 P05_04 P05_37
NAU1369
Gate3CE04 BNL2835 CIR119 NAU4091
pVNC244
E5M1-M363.3 Gate2AC11 pAR571 pAR972
DPL0461
E5M4_345 JESPR070 BNL1513
HAU1432
CIR274 A1107 DPL0068
NAU1302
G1074 Gate4AD12 TMB2386
NAU0478
UCcg10596_89
CIR354 NAU3424 CLU2089
CLU684
NAU4099 NAU1302 NAU3201
JESPR305
CLU12 BNL2961 Gate4BG06 NAU5399
Gate4AD12 pGH244 CLU2796
CLU527 NAU3424 NAU3605
Gh273 pAR476 pGH797 NAU2439 JESPR078
NAU3562 CLU607 pAR09A08 pAR594 CLU4317
NAU1197 HAU2738 Gate1DE02 pAR010 Rab11
E1M3-M109.4 NAU2169 A1562 pAR785
NAU1505 DPL0031 pGH317 CLU13
JESPR291 Coau1E19 me3em2-175 BNL3638
NAU0891
HAU3247 CLU2762 HAU3293 CLU292 CLU1305
TMD05-225
CLU23 E3M2_420 CLU1476
HAU1846 CLU3977
TMB0072
CLU177 CLU1630 TMP14-240
TMB1639 CLU1048
BNL3474 E43M52-M438.6 Gate4CF12
E3M5_184
DPL0534 em5DC1-110
GW57-160
GW58-160 DPL0202 HAU0722 TMB0429 Gate4DE09
pAR07D04
NAU2292 E41M1-M216.6 TMB1289
CIR061 BNL2568 CLU1339
Gate3DD06 NAU6310
E7M11-150
Gh128 E7M1_193 CLU1940 E43M52-M334.3
Gate1DF03 Gate2CC12 Gate3DE09 Gate4BG11 NAU1531
HAU2522
NAU2240 BNL0387 CIR070
NAU6213 Gate1BG03 pAR07D04 NAU3773 CLU995
NAU2631 NAU6642 E43M53-M57.8
NAU3954 CLU2660 E43M52-M373.0 CLU2934 NAU6235
CLU1290 TML21-300
NAU2619 JESPR033 NAU6616 BNL2499
CLU2834 BNL2655 CLU2116 TMA5-180 em1DC1-205
NAU2926 GW34-305 Coau2A11 Gate4AH08 Unig27F10
NAU6488 NAU6169 CLU4137 NAU3769 BNL3604
CLU1261 NAU5335 EM1OD26-500 NAU6389 E43M54-M169.4
GW17-350 CLU3611 BNL3474
Gh237 E42M1-M134.6 Gh272
E1M4-M238.3 HAU1533 DPL0228 CLU92 TMB0555
TMB1182
P10_56 pAR08F09 E6M5_350 TMB1244 DPL0231
CIR289 DPL0146
MUSB0632 A1667 Gate2CD01 Gate4AC08 pAR118
pGH318 TMB1190 BNL1017 Gh171
E1M2-M378.1 MUSB0255 E6M5_237
MUSB0641 A1632 pAR418 pVNC164 BNL3084
A1168 CM43 E4M7_134 A1590 G1013
Gate2CF02 P05_24 TMB0016
E4M3_94 CLU365 Gate1CC03 E43M2-M276.3 E3M2_245
E31M7-M115.4 Gh573 NAU1262 A1662 E1M2_360
E5M8_176 E3M7_360 Gate1DB08 pAR332 pAR418
E7M4_287
TMB1745
CLU105 E42M2-M190.9
E2M6_77 CG03
BNL0252
BNL2582 NAU3667 NAU0435 CLU26 CLU3873
DPL0604 NAU3910 A1348 Coau2C15 Gate1AG10
pAR04_11 pAR09H09 pAR309
NAU3771
E14M4-170
E15M16-100 NAU1350 CIR278
NAU3455
E14M2-320 CLU1261 G1010 MUSB0175 DC1OD8-250
UCcg10011_47
CLU2055
CIR061
CIR413
CIR289
BNL3627 Gate1CA10
CLU13 CIR343 JESPR307
pAR789
pAR03_23
A1197 A1197 A1658
NAU3904 P07_04 JESPR183
Gate2BC08 Unig23H12
pAR01A05
NAU3324
Gate3CA01 pARC_04
Gate3CA01
Gate4CB07
G1276
Gate3BE04 NAU3221
A1811 pAR490
Gate4DG08
Y1187
NAU3224

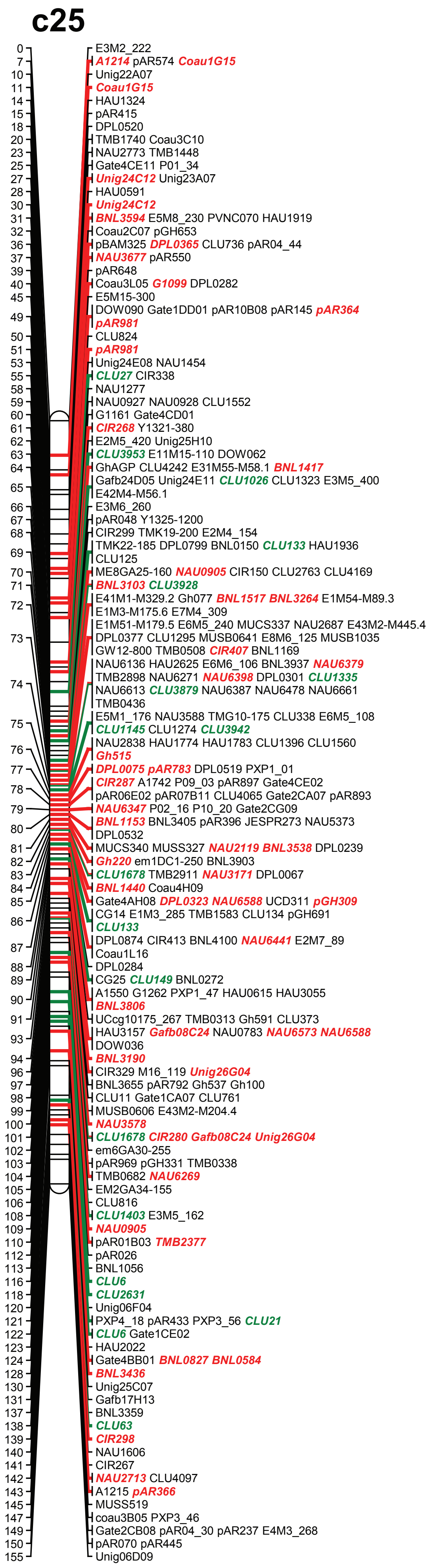
c25
0
7
10
11
14
15
18
20
23
25
27
28
30
31
32
36
37
39
40
45
49
50
51
53
55
58
59
60
61
62
63
64
65
66
67
68
69
70
71
72
73
74
75
76
77
78
79
80
81
82
83
84
85
86
87
88
89
90
91
93
94
96
97
98
99
100
101
102
103
104
105
106
108
109
110
112
113
116
118
120
121
122
123
124
128
130
131
137
138
139
140
141
142
143
145
147
149
150
155
E3M2_222
A1214 pAR574 Coau1G15
Unig22A07
Coau1G15
HAU1324
pAR415
DPL0520
TMB1740 Coau3C10
NAU2773 TMB1448
Gate4CE11 P01_34
Unig24C12 Unig23A07
HAU0591
Unig24C12
BNL3594 E5M8_230 PVNC070 HAU1919
Coau2C07 pGH653
pBAM325 DPL0365 CLU736 pAR04_44
NAU3677 pAR550
pAR648
Coau3L05 G1099 DPL0282
E5M15-300
DOW090 Gate1DD01 pAR10B08 pAR145 pAR364
pAR981
CLU824
pAR981
Unig24E08 NAU1454
CLU27 CIR338
NAU1277
NAU0927 NAU0928 CLU1552
G1161 Gate4CD01
CIR268 Y1321-380
E2M5_420 Unig25H10
CLU3953 E11M15-110 DOW062
GhAGP CLU4242 E31M55-M58.1 BNL1417
Gafb24D05 Unig24E11 CLU1026 CLU1323 E3M5_400
E42M4-M56.1
E3M6_260
pAR048 Y1325-1200
CIR299 TMK19-200 E2M4_154
TMK22-185 DPL0799 BNL0150 CLU133 HAU1936
CLU125
ME8GA25-160 NAU0905 CIR150 CLU2763 CLU4169
BNL3103 CLU3928
E41M1-M329.2 Gh077 BNL1517 BNL3264 E1M54-M89.3
E1M3-M175.6 E7M4_309
E1M51-M179.5 E6M5_240 MUCS337 NAU2687 E43M2-M445.4
DPL0377 CLU1295 MUSB0641 E8M6_125 MUSB1035
GW12-800 TMB0508 CIR407 BNL1169
NAU6136 HAU2625 E6M6_106 BNL3937 NAU6379
TMB2898 NAU6271 NAU6398 DPL0301 CLU1335
NAU6613 CLU3879 NAU6387 NAU6478 NAU6661
TMB0436
E5M1_176 NAU3588 TMG10-175 CLU338 E6M5_108
CLU1145 CLU1274 CLU3942
NAU2838 HAU1774 HAU1783 CLU1396 CLU1560
Gh515
DPL0075 pAR783 DPL0519 PXP1_01
CIR287 A1742 P09_03 pAR897 Gate4CE02
pAR06E02 pAR07B11 CLU4065 Gate2CA07 pAR893
NAU6347 P02_16 P10_20 Gate2CG09
BNL1153 BNL3405 pAR396 JESPR273 NAU5373
DPL0532
MUCS340 MUSS327 NAU2119 BNL3538 DPL0239
Gh220 em1DC1-250 BNL3903
CLU1678 TMB2911 NAU3171 DPL0067
BNL1440 Coau4H09
Gate4AH08 DPL0323 NAU6588 UCD311 pGH309
CG14 E1M3_285 TMB1583 CLU134 pGH691
CLU133
DPL0874 CIR413 BNL4100 NAU6441 E2M7_89
Coau1L16
DPL0284
CG25 CLU149 BNL0272
A1550 G1262 PXP1_47 HAU0615 HAU3055
BNL3806
UCcg10175_267 TMB0313 Gh591 CLU373
HAU3157 Gafb08C24 NAU0783 NAU6573 NAU6588
DOW036
BNL3190
CIR329 M16_119 Unig26G04
BNL3655 pAR792 Gh537 Gh100
CLU11 Gate1CA07 CLU761
MUSB0606 E43M2-M204.4
NAU3578
CLU1678 CIR280 Gafb08C24 Unig26G04
em6GA30-255
pAR969 pGH331 TMB0338
TMB0682 NAU6269
EM2GA34-155
CLU816
CLU1403 E3M5_162
NAU0905
pAR01B03 TMB2377
pAR026
BNL1056
CLU6
CLU2631
Unig06F04
PXP4_18 pAR433 PXP3_56 CLU21
CLU6 Gate1CE02
HAU2022
Gate4BB01 BNL0827 BNL0584
BNL3436
Unig25C07
Gafb17H13
BNL3359
CLU63
CIR298
NAU1606
CIR267
NAU2713 CLU4097
A1215 pAR366
MUSS519
coau3B05 PXP3_46
Gate2CB08 pAR04_30 pAR237 E4M3_268
pAR070 pAR445
Unig06D09

c26

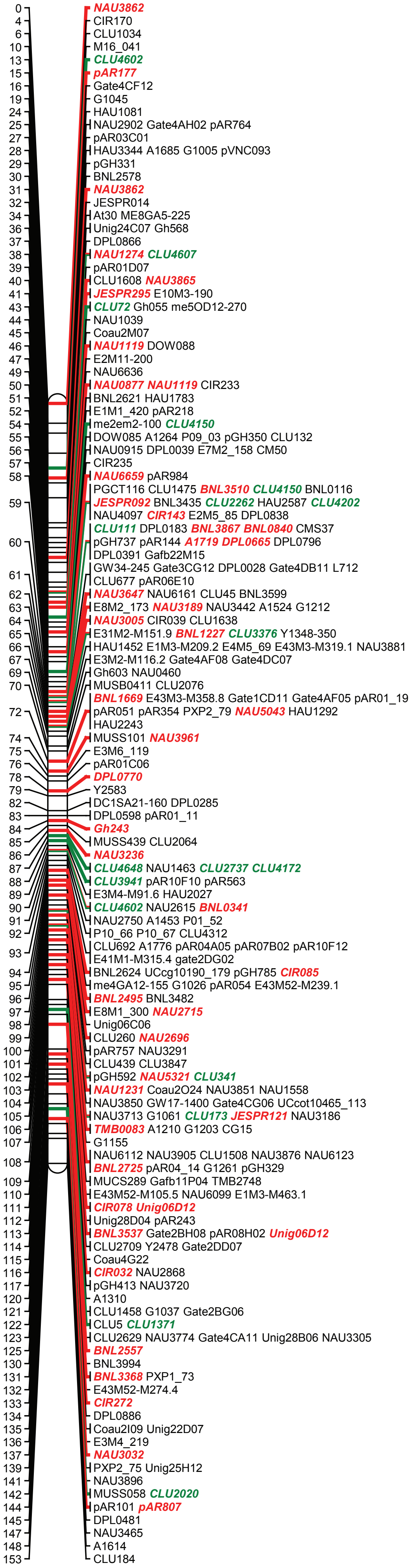

0
4
6
10
13
15
16
19
24
25
27
28
29
30
31
32
34
36
37
38
39
40
41
43
44
45
46
47
49
50
51
52
54
55
56
57
58
59
60
61
62
63
64
65
66
67
69
70
72
74
75
76
78
79
82
83
84
85
86
87
88
89
90
91
92
93
94
95
96
97
98
99
100
101
102
103
104
105
106
107
108
109
110
111
112
113
114
115
116
117
120
121
122
123
125
130
131
132
133
134
135
136
137
139
141
142
144
145
147
148
153
NAU3862
CIR170
CLU1034
M16_041
CLU4602
pAR177
Gate4CF12
G1045
HAU1081
NAU2902 Gate4AH02 pAR764
pAR03C01
HAU3344 A1685 G1005 pVNC093
pGH331
BNL2578
NAU3862
JESPR014
At30 ME8GA5-225
Unig24C07 Gh568
DPL0866
NAU1274 CLU4607
pAR01D07
CLU1608 NAU3865
JESPR295 E10M3-190
CLU72 Gh055 me5OD12-270
NAU1039
Coau2M07
NAU1119 DOW088
E2M11-200
NAU6636
NAU0877 NAU1119 CIR233
BNL2621 HAU1783
E1M1_420 pAR218
me2em2-100 CLU4150
DOW085 A1264 P09_03 pGH350 CLU132
NAU0915 DPL0039 E7M2_158 CM50
CIR235
NAU6659 pAR984
PGCT116 CLU1475 BNL3510 CLU4150 BNL0116
JESPR092 BNL3435 CLU2262 HAU2587 CLU4202
NAU4097 CIR143 E2M5_85 DPL0838
CLU111 DPL0183 BNL3867 BNL0840 CMS37
pGH737 pAR144 A1719 DPL0665 DPL0796
DPL0391 Gafb22M15
GW34-245 Gate3CG12 DPL0028 Gate4DB11 L712
CLU677 pAR06E10
NAU3647 NAU6161 CLU45 BNL3599
E8M2_173 NAU3189 NAU3442 A1524 G1212
NAU3005 CIR039 CLU1638
E31M2-M151.9 BNL1227 CLU3376 Y1348-350
HAU1452 E1M3-M209.2 E4M5_69 E43M3-M319.1 NAU3881
E3M2-M116.2 Gate4AF08 Gate4DC07
Gh603 NAU0460
MUSB0411 CLU2076
BNL1669 E43M3-M358.8 Gate1CD11 Gate4AF05 pAR01_19
pAR051 pAR354 PXP2_79 NAU5043 HAU1292
HAU2243
MUSS101 NAU3961
E3M6_119
pAR01C06
DPL0770
Y2583
DC1SA21-160 DPL0285
DPL0598 pAR01_11
Gh243
MUSS439 CLU2064
NAU3236
CLU4648 NAU1463 CLU2737 CLU4172
CLU3941 pAR10F10 pAR563
E3M4-M91.6 HAU2027
CLU4602 NAU2615 BNL0341
NAU2750 A1453 P01_52
P10_66 P10_67 CLU4312
CLU692 A1776 pAR04A05 pAR07B02 pAR10F12
E41M1-M315.4 gate2DG02
BNL2624 UCcg10190_179 pGH785 CIR085
me4GA12-155 G1026 pAR054 E43M52-M239.1
BNL2495 BNL3482
E8M1_300 NAU2715
Unig06C06
CLU260 NAU2696
pAR757 NAU3291
CLU439 CLU3847
pGH592 NAU5321 CLU341
NAU1231 Coau2O24 NAU3851 NAU1558
NAU3850 GW17-1400 Gate4CG06 UCcot10465_113
NAU3713 G1061 CLU173 JESPR121 NAU3186
TMB0083 A1210 G1203 CG15
G1155
NAU6112 NAU3905 CLU1508 NAU3876 NAU6123
BNL2725 pAR04_14 G1261 pGH329
MUCS289 Gafb11P04 TMB2748
E43M52-M105.5 NAU6099 E1M3-M463.1
CIR078 Unig06D12
Unig28D04 pAR243
BNL3537 Gate2BH08 pAR08H02 Unig06D12
CLU2709 Y2478 Gate2DD07
Coau4G22
CIR032 NAU2868
pGH413 NAU3720
A1310
CLU1458 G1037 Gate2BG06
CLU5 CLU1371
CLU2629 NAU3774 Gate4CA11 Unig28B06 NAU3305
BNL2557
BNL3994
BNL3368 PXP1_73
E43M52-M274.4
CIR272
DPL0886
Coau2I09 Unig22D07
E3M4_219
NAU3032
PXP2_75 Unig25H12
NAU3896
MUSS058 CLU2020
pAR101 pAR807
DPL0481
NAU3465
A1614
CLU184
