# Supplementary material for: A High Density Consensus Genetic Map of Tetraploid Cotton That Integrates Multiple Component Maps through Molecular Marker Redundancy Check
Source: PLoS One. 2012 Sep 24;7(9):e45739. doi: 10.1371/journal.pone.0045739 (PMC3454346; doi:10.1371/journal.pone.0045739)
Supplement: Supplementary file 3 [file pone.0045739.s005.doc]

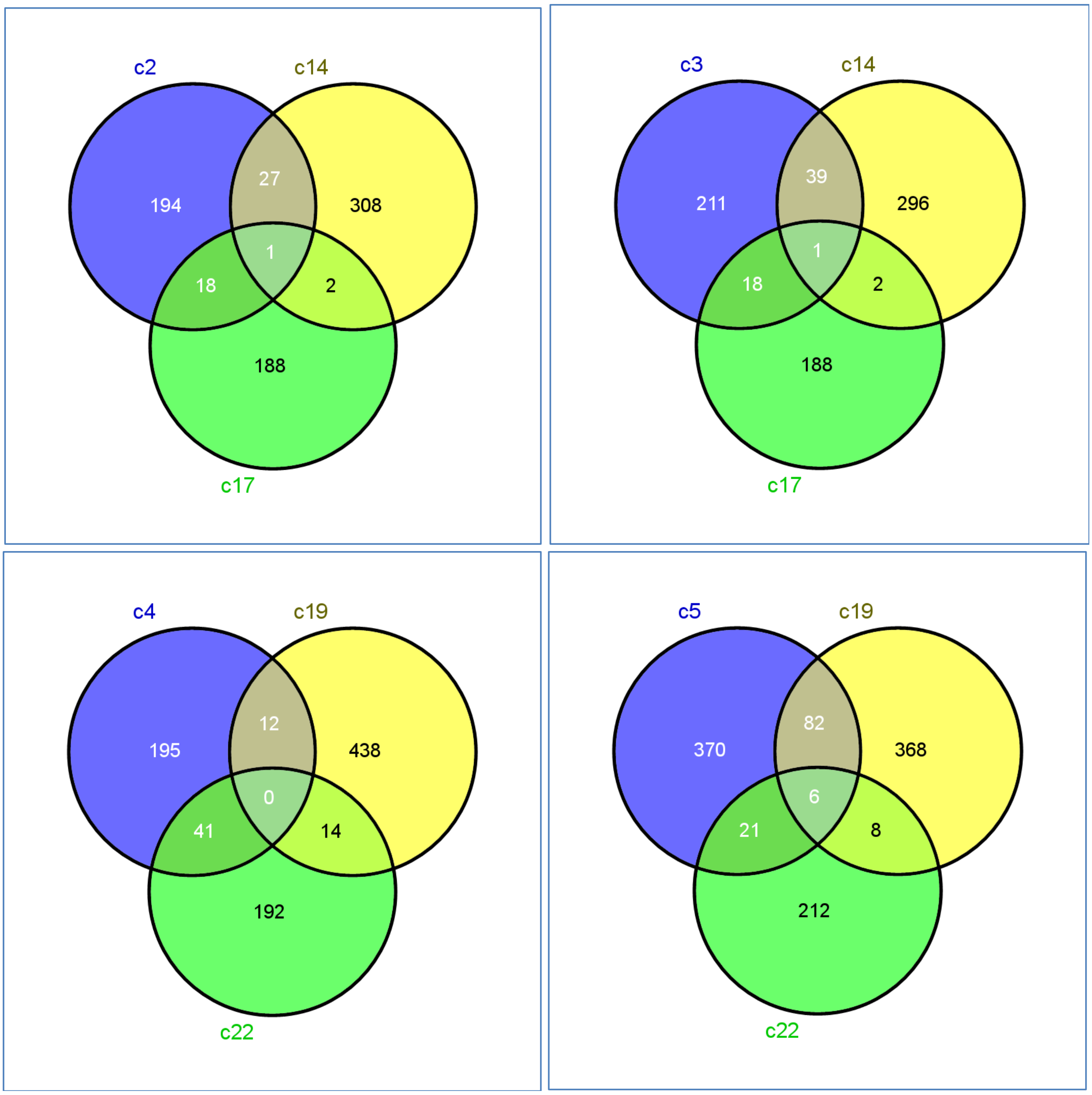
c2
c14
c17
194
27
308
1
18
2
188
c3
c14
c17
211
39
296
1
18
2
188
c4
c19
c22
195
12
438
0
41
14
192
c5
c19
c22
370
82
368
6
21
8
212
